# Heteroaryl-Capped Hydroxamic Acid Derivatives with Varied Linkers: Synthesis and Anticancer Evaluation with Various Apoptosis Analyses in Breast Cancer Cells, Including Docking, Simulation, DFT, and ADMET Studies

**DOI:** 10.3390/ph18081148

**Published:** 2025-08-01

**Authors:** Ekta Shirbhate, Biplob Koch, Vaibhav Singh, Akanksha Dubey, Haya Khader Ahmad Yasin, Harish Rajak

**Affiliations:** 1 Medicinal Chemistry Research Laboratory, Department of Pharmacy, Guru Ghasidas University, Bilaspur 495009, CG, India; ekta.shirbhate@gmail.com (E.S.); singhvaibhav11pharma@gmail.com (V.S.); 2 Department of Zoology, Banaras Hindu University, Varanasi 221005, UP, India; biplob@bhu.ac.in (B.K.); dubeyakanksha1023@gmail.com (A.D.); 3 Department of Pharmaceutical Sciences, College of Pharmacy and Health Sciences, Ajman University, Ajman P.O. Box 346, United Arab Emirates; 4 Center of Medical and Bio-Allied Health Sciences Research, Ajman University, Ajman P.O. Box 346, United Arab Emirates

**Keywords:** anticancer, apoptosis, cell cycle arrest, hydroxamide, HDAC inhibitors, docking, simulation, toxicity

## Abstract

**Background/Objectives:** Cancer suffers from unresolved therapeutic challenges owing to the lack of targeted therapies and heightened recurrence risk. This study aimed to investigate the new series of hydroxamate by structurally modifying the pharmacophore of vorinostat. **Methods:** The present work involves the synthesis of 15 differently substituted 2*H*-1,2,3-triazole-based hydroxamide analogs by employing triazole ring as a cap with varied linker fragments. The compounds were evaluated for their anticancer effect, especially their anti-breast cancer response. Molecular docking and molecular dynamics simulations were conducted to examine binding interactions. **Results:** Results indicated that among all synthesized hybrids, the molecule **VI(i)** inhibits the growth of MCF-7 and A-549 cells (GI_50_ < 10 μg/mL) in an antiproliferative assay. Compound **VI(i)** was also tested for cytotoxic activity by employing an MTT assay against A549, MCF-7, and MDA-MB-231 cell lines, and the findings indicate its potent anticancer response, especially against MCF-7 cells with IC_50_ of 60 µg/mL. However, it experiences minimal toxicity towards the normal cell line (HEK-293). Mechanistic studies revealed a dual-pathway activation: first, apoptosis (17.18% of early and 10.22% of late apoptotic cells by annexin V/PI analysis); second, cell cycle arrest at the S and G2/M phases. It also promotes ROS generation in a concentration-dependent manner. The HDAC–inhibitory assay, extended in silico molecular docking, and MD simulation experiments further validated its significant binding affinity towards HDAC 1 and 6 isoforms. DFT and ADMET screening further support the biological proclivity of the title compounds. The notable biological contribution of **VI(i)** highlights it as a potential candidate, especially against breast cancer cells.

## 1. Introduction

Despite substantial breakthroughs in cancer therapy, persistent problems such as drug resistance, tumor heterogeneity, and poor therapeutic selectivity continue to impede successful cancer treatment. Current anticancer drugs usually fail due to innate and acquired resistance mechanisms, with multidrug resistance accounting for more than 90% of cancer-related deaths [1,2]. Many biological activities, such as cell survival, death, and proliferation, depend on the precise regulation of gene expression [3,4]. It is also well-established that any alterations to the genome and epigenome contribute to the onset of cancer [5]. Epigenetic alterations like post-translational modification of histone protein, DNA methylation, etc., alter the structural makeup of chromatin. Histone acetylation and deacetylation are two post-translational changes that have been identified as crucial for controlling the expression of genes implicated in cancer pathophysiology [6,7]. The acetylation and deacetylation status of histone is regulated by two functionally antagonistic enzymes called histone acetyltransferases (HATs) and histone deacetylases (HDACs), which, together, manage gene transcription [8,9]. The enzyme HAT catalyzes the acetylation of the ε-amino assemblage of a lysine residue on the N-terminal of the histone tail, thus leading to chromatin relaxation, which permits transcription factors to bind to DNA, and, eventually, the expression of genes [10]. In contrast, HDACs cause chromatins to shut by removing the acetyl clutches from the histone lysine amino terminals. Condensed chromatin prevents transcription factors from accessing DNA, which suppresses the expression of genes—particularly the expression of tumor suppressor oncogenes [11,12]. An imbalance in the activity of HATs and HDACs frequently causes abnormal gene expression, resulting in various epigenetic diseases, including cancer [13]. Importantly, unlike variations in the DNA sequence, the epigenetic modifications are reversible, highlighting their immense potential in targeting disrupted epigenetic pathways for developing novel anticancer drugs [14].

The known HDACs, based on their sequence homology, are classified into different categories, namely, class I (HDAC 1, 2, 3, 8); class II, which is again split into IIa (HDAC 4, 5, 7, 9) and IIb (HDAC 6, 10); class III, sirtuins (SIRT 1-7); and finally, class IV (HDAC 11) [15]. Different biological processes, like meiosis, chromatic decondensation, cellular development, cellular differentiation, cell death, and angiogenesis, are prone to be affected by silencing or inhibiting HDACs in various cancer cell types [16]. HDACs have therefore become crucial therapeutic targets in the fight against cancer. The discovery of HDAC inhibitors, together with an illumination of the roles and mechanisms of HDAC in carcinogenesis, has offered a potent new avenue for cancer treatment [17].

The US-FDA, in October 2006, legalized the first hydroxamate-based HDAC inhibitor, vorinostat (SAHA), which finds application in curing rare cutaneous T-cell lymphoma. Later, romidepsin, belinostat, and panobinostat were also legalized by the FDA for the cure of cutaneous T-cell lymphoma (CTCL), peripheral T-cell lymphoma (PTCL), and multiple myeloma (MM), respectively [18]. The CFDA approved a benzamide-grounded class I HDAC-selective inhibitor chidamide (Epidaza) for treating relapsed or refractory PTCL [19]. Numerous promising HDAC inhibitors are under various phases of clinical investigations against different malignancies [20]. Hence, the advance of new HDAC inhibitors as anticancer representatives has turned out to be the most intense and appealing area in drug discovery.

Generally, a canonical structure of a typical HDAC inhibitor comprises a three-motif pharmacophoric feature: a capping group, such as hydrophobic/bulky moieties (such as aromatic or heteroaromatic rings), establishing interaction with the rim at the active pocket of HDAC enzyme; a zinc-binding group (ZBG) for chelating Zn^2+^ in the HDAC active site; and a linker, mediating assembly between the cap and ZBG and setting up communication with the hydrophobic passage of the active site [21,22]. The selectivity of HDAC subtypes seems to be influenced by contact with residues at the ingress of the binding pocket of the enzyme. Also, every pharmacophore-including the cap, the linker and the ZBG-of the HDAC inhibitor contributes to the potency of the compound [23]. Designing more potent inhibitors involves exploring new cap groups and suitable linkers to optimize their interaction with HDACs, which could enhance their therapeutic efficacy against cancer [24]. Triazole derivatives represent a vital category of heterocyclic compounds with significant roles in medicinal chemistry, particularly as therapeutic agents [25]. Many triazole analogs find widespread use in clinical therapy [25,26]. The burgeoning interest in 1,2,3-triazoles among the medicinal chemistry community stemmed from advancements in the Huisgen 1,3-dipolar cycloaddition method, facilitating the straightforward synthesis of these heterocyclic compounds [27,28,29]. The 1,2,3-triazole moiety performs a crucial structural role in the design of HDAC inhibitors by associating with amino acid residues situated on the rim of HDAC catalytic pocket, thereby improving both potency and selectivity [30,31,32]. This ring structure is highly stable under redox conditions and resistant to metabolic degradation and hydrolysis [27]. Chen et al., in their study, reported that the triazole-linked SAHA-like hydroxamates were up to four times more potent than SAHA, with optimal activity observed when five- or six-membered methylene spacers were present between the triazole and zinc-binding hydroxamate groups. Beyond their favorable binding characteristics, triazoles can serve as bioisosters for pharmacokinetically challenging molecules like amides and ketones [32]. Kalinin et al., through their study of smHDAC8, demonstrated that triazole-based inhibitors can selectively block specific HDAC isoforms [33]. Furthermore, Sun et al. discovered that these inhibitors not only exert direct anticancer effects but also enhance immune responses, particularly when used in combination with immune checkpoint inhibitors [34]. The versatility of the triazole group, its ease of synthesis via click chemistry, and its likely resistance to intracellular peptidases contribute to the development of more potent and selective hydroxamic acid derivatives.

### Rationale of This Research

**Figure 1:**
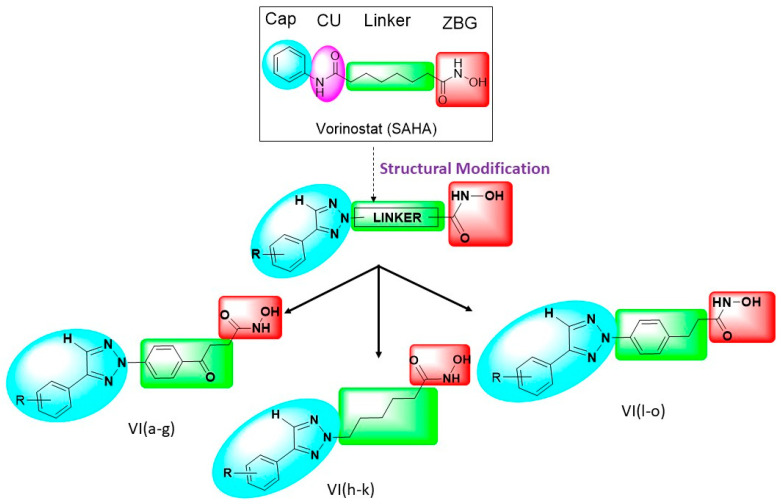
Rational design of 1,2,3-triazole-based hydroxamides as HDAC inhibitors. The different colors illustrate different pharmacophore of HDAC inhibitor. The cap group is highlighted in blue, connecting unit in pink, linker in green and ZBG in red.

In the current research, a sequence of compounds was outlined and synthesized based on the structural framework of vorinostat (SAHA). It is widely recognized that the triazole heterocycle functions as a common scaffold incorporated into the architectures of many biologically active substances [35]. The triazole ring functions as a bioisostere of the amide group due to similarities in H-bond acceptor capability, the distance between the substituents, and the dipolar character [36]. The rationale diagram presented in Figure 1 illustrates the possible systematic structural modifications that can be made to vorinostat (SAHA), a well-established HDAC inhibitor, through the incorporation of a triazole-based cap group. The unique properties of the triazole moiety, like its electronic charge distribution, stability, and ability to interact with biological targets through hydrogen bonding and π-π stacking interactions, have caused it to gain significant attention in medicinal chemistry. Because of above-listed characteristics, triazole derivatives seem to be potential candidates for HDAC inhibition. Triazoles, in addition, have shown promising anticancer potential through the modulation of epigenetic mechanisms, which further supports their applicability in our design approach. In our study, we investigated several alterations in the ZBG, linker, and cap region to maximize interactions with HDAC isoforms. A triazole-based scaffold was introduced as a cap group to improve potency and selectivity while preserving favorable pharmacokinetic properties. The linker region was modified by integrating different acids to increase the flexibility and binding affinity of these inhibitory molecules. However, the ZBG was undisturbed and kept in place to guarantee efficient chelation of the zinc ion at the active site of the HDAC enzymes. Our goal was to create new HDAC inhibitors with improved efficacy against particular isoforms linked to cancer by methodically implementing these changes.

Our study introduces the development, synthesis, and assessment of hydroxamic acid derivatives containing a 1,2,3-triazole ring as an HDAC inhibitor. The synthesized compounds were subjected to SRB assay, MTT assay, ROS determination, cell cycle analysis, and apoptotic study to evaluate their biological activities. Furthermore, molecular docking, MD simulation, binding free energy, and pharmacokinetic studies were conducted to study the HDAC–inhibitory activity at an atomic level.

## 2. Results and Discussion

### 2.1. Chemistry of Synthesized Compounds

**Scheme 1:**
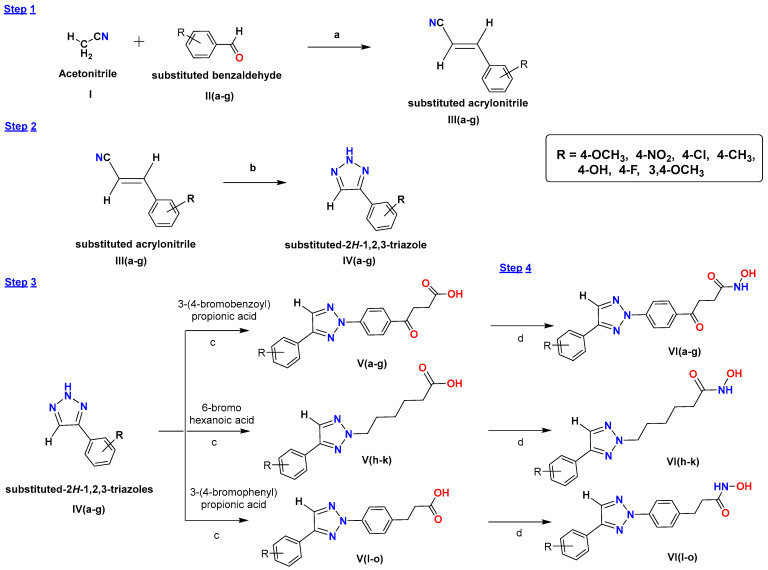
Synthesis of compounds **VI(a–o)**. Reagents and conditions: (a) 5% NaOMe, MeOH, stir, 4–6 h, 40–45 °C; (b) NaN_3_, NH_4_Cl, DMF/H_2_O, reflux, 12–14 h; (c) NaHCO_3_, DMF; stir, 6–7 h, 65–80 °C; (d) ClCOO-*i*Bu, Et_3_N, NH_2_OH.HCl, THF, stir, 20–24 h, RT.

The procedure for the synthesis is illustrated in Scheme 1. It consisted of four steps. The first step consisted of the addition reaction between acetonitrile **I** with substituted benzaldehyde **[II(a–g)]** in the presence of 5% sodium methoxide solution in methanol, resulting in the formation of substituted acrylonitrile analogs **[III(a–g)]** [37]. The **[III(a–g)]** underwent 1,3-dipolar cycloaddition reaction when refluxed for 5–12 h with NaN_3_ and NH_4_Cl at a ratio of 1:3:3, causing the formation of substituted-2*H*-1,2,3-triazole analogs **[IV(a–g)]** [37]. The third step involved the formation of substituted 1,2,3-triazole-based hydroxamic acid analogs **[V(a–o)]** from the reaction of compound **[IV(a–o)]** with three different acids: 3-(4-bromobenzoyl)propionic acid, 6-bromohexanoic acid, and 3-(4-bromophenyl)propionic acid. Finally, in the last step, **[V(a–o)]**, possessing aliphatic/aromatic linkers, was reacted with hydroxylamine hydrochloride to form the final compound: substituted 1,2,3-triazole-based hydroxamide analogs **[VI(a–o)]**. The intermediates formed at different steps were monitored by TLC and purified by repeated washing and recrystallization via suitable solvents. The structure and purity of the synthesized derivatives were confirmed by IR, ^1^H-NMR, ^13^C-NMR, and mass spectrometry.

### 2.2. Description of FTIR Spectra

FTIR spectroscopy provides an initial structural validation of the synthesized compound. The characteristic peaks were found at 3650–3300 cm^−1^ (O-H, N h stretching), 3100–3000 cm^−1^ (aromatic C-H), 2950–2850 cm^−1^ (aliphatic C-H), 2220–2260 cm^−1^ (nitrile), 1750–1650 cm^−1^ (C=O stretching), and 1620–1450 cm^−1^ (aromatic C=C). The acrylonitrile intermediate III(a) had indicative nitrile stretching at 2563.40 cm^−1^ and aromatic C=C peaks at 1610.53 and 1521.10 cm^−1^. The triazole **IV(a)** had aromatic =C h stretching at 3099.69 cm^−1^, which confirmed cyclization in that step of the reaction. The acid derivatives **V(a–o)** had broad O h stretching at 3365–3463 cm^−1^. Additionally, halogen-substituted compounds had specific C-Cl and C-F stretching bands in their respective compounds. The hydroxamide series **VI(a–o)** consistently showed broad O h stretching at 3150–3523 cm^−1^, secondary amide N h at 3286–3390 cm^−1^, and dual carbonyl stretches for ketone (1697–1741 cm^−1^) and amide (1642–1683 cm^−1^) functionalities. It also had peaks that were specific to each substituent, such as N=O stretching for nitro compounds, C-Cl stretching for chloro compounds, and multiple C-O ether bands for methoxy-containing compounds. All of these peaks confirmed the synthesis of hydroxamide analogs from acrylonitrile and substituted benzaldehydes.

### 2.3. Description of NMR Spectra

The synthesized compounds were characterized through ^1^H NMR and ^13^C NMR spectroscopy. Their spectrum showed that the spectral signatures were different for every compound, which demonstrated that a successful structural change happened throughout the synthetic pathway. The acrylonitrile intermediate **III(a)** had methoxy protons at δ 3.78 ppm and methylene protons at δ 4.88 ppm. Aromatic protons were in the anticipated δ 6.98–7.88 range with coupling constants of *J* = 8.4–13.4 Hz. The triazole compound **IV(a)** showed successful cyclization with a diagnostic triazole C h proton at δ 8.10 ppm, and the acid series **V(a–o)** exhibited characteristic proton signals at δ 2.69–3.90 ppm with methylene triplets showing *J* = 7.8 Hz coupling. The substituted 1,2,3-triazole-based hydroxamide analogs **VI(a–o)** showed N h and O h proton signals at δ 9.20–11.52 ppm.

The ^13^C NMR data also confirmed the structure with following observations. The carbonyl carbons were in the right downfield positions: ketones at δ 197–199 ppm, carboxylic acids at δ 174–175 ppm, and amides at δ 170–171 ppm. The triazole ring carbons were at δ 144–150 ppm, and the nitrile carbons were at δ 118.80 ppm, which showed that the synthetic transformations worked.

### 2.4. Description of Physical and Chemical Properties

The molecular weights of the synthesized compounds ranged from 289.165 to 397.150. The molecular weight, R_f_ value, and melting points of the synthesized compounds are shown along with their spectral data. All compounds were obtained in high yields, demonstrating the effectiveness of the synthetic procedures. In comparison to other compounds in the series, some compounds, such as **VI(d)**, **VI(h)**, and **VI(i)**, were obtained in high yields (~58%).

### 2.5. Antiproliferative Assay

**Figure 2:**
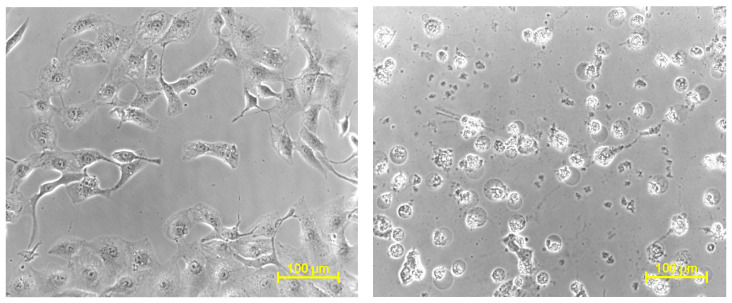
Photographs of **VI(i)** showing good antiproliferative activity in an SRB assay when tested on A-549 and MCF-7 cell lines.

The in vitro anticancer assessment of synthesized compounds was performed against A-549 and MCF-7 human cancer cell lines through an SRB assay [38,39] (Table 1). The compounds **VI(b)**, **VI(c)**, **VI(e)**, and **VI(i)** had remarkable GI_50_ values of <10 g/mL against both the selected cell lines. Figure 2 shows an inhibition in both A-549 and MCF-7 cell lines upon treatment with **VI(i)**. The compound **VI(a)** also exhibited good anticancer action with GI_50_ value of 19.35 and 15.13 μg/mL against A-549 and MCF-7, respectively. The compounds **VI(d)**, **VI(g)**, and **VI(j)** also diminished the multiplication of breast cancer cell with the GI_50_ values of 12.91, 15.0, and 17.0 μg/mL, respectively. The growth curves representing the GI_50_ values of synthesized 1,2,3-triazole-based hydroxamide analogs against the two tested cell lines are shown in Figure S1 (Supplementary Materials).

### 2.6. MTT Assay

**Figure 3:**
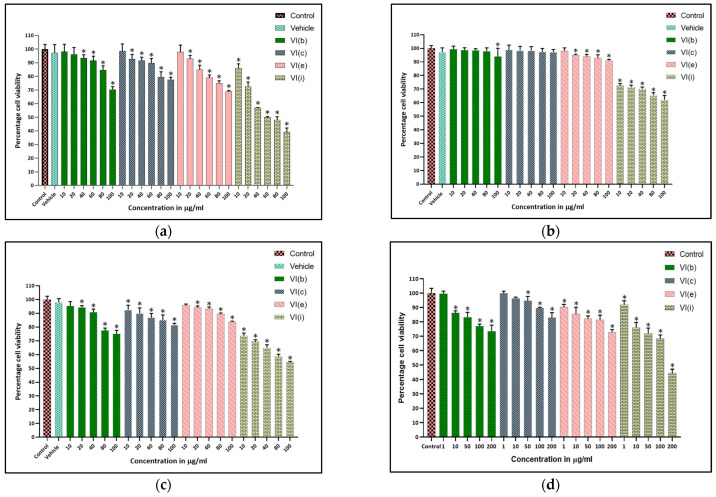
Graphical representation of cytotoxicity assay of selected compounds—**VI(b)**, **VI(c)**, **VI(e)**, and **VI(i)**—treated against human breast cancer [(**a**) MCF-7; (**b**) MDA-MB-231], human lung carcinoma [(**c**) A549], and normal [(**d**) HEK-293] cells at different concentrations in µg/mL. Significant difference was considered at * *p* < 0.05 with respect to control.

Cytotoxicity assay (MTT assay) was performed to measure cellular viability [40] after exposing selected compounds **[VI(b)**, **VI(c)**, **VI(e)**, and **VI(i)]** against a panel of cell lines. Compound **VI(i)** exhibited potent anticancer activity; the obtained IC_50_ value was at 60 µg/mL. Figure 3 also indicates that compound **VI(i)** displayed stronger anticancer activity against MCF-7 than MDA-MB-231 and A549 cell lines, as the obtained IC_50_ data (in MDA-MB-231 and A549) were found at a higher concentration (>100 µg/mL). Further, the cytotoxicity of VI series was also determined against normal cell line (HEK-293), and the obtained result revealed that the compounds were non-toxic to normal cellular functions. Several studies suggest that hydroxamic acid derivatives possess high anticancer potential. Thus, the present study demonstrates similar results, i.e., that compound **VI(i)** showed strong antiproliferative efficacy against MCF-7 breast cancer cells. It worth mentioning that since compound **VI(i)** displayed remarkable anticancer activity in MCF-7 cells; hence, all further biological experiments were carried out with the above cell line.

**VI(i)** showed moderate cytotoxic activity in the MTT assay against MCF-7 cells while demonstrating pronounced antiproliferative effects in the SRB assay. It exhibited relatively lower toxicity to normal cells compared to cancer cells, demonstrating a moderate degree of selectivity that could be further optimized. However, there are various reasons for the discrepancy of the results between the two assay methods. First, the MTT assay assessed metabolic activity by mitochondrial dehydrogenase activity, whereas the SRB assay counted the cells based on total protein content. These fundamentally distinct testing approaches possibly produced disparate results, particularly for substances that alter cellular metabolism rather than cell proliferation. Second, the two methods differed in their test exposure periods. The SRB assay was performed under a 48 h exposure period, and the MTT test was done in 24 h drug exposure. The metabolic changes often precede change in total protein content during cellular response, possibly contributing to the discrepancy in result. Additionally, following advised storage conditions, like suitable temperature, shielding from light and moisture, using airtight and inert containers, and ensuring a contamination-free environment, are quite important.

### 2.7. Apoptosis Study Using Acridine Orange/Ethidium Bromide Dual Staining Method

**Figure 4:**
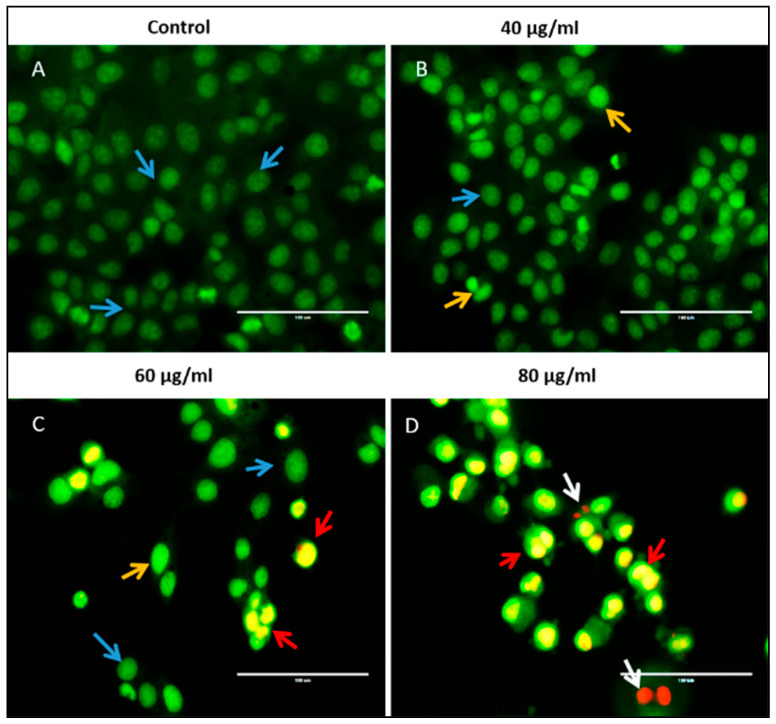
Morphological changes of MCF-7 cells following exposure to compound **VI(i)** within the range of its IC_50_ value (40, 60, and 80 µg/mL) are depicted under the inverted fluorescence microscope through the acridine orange/ethidium bromide (AO/EtBr) dual-staining technique. Where, (**A**) Control MCF-7 cells, and (**B**) lower concentration (40 µg/mL), (**C**) IC_50_ (60 µg/mL), (**D**) higher concentration (80 µg/mL) of compound **VI(i)** treated cells. Live cells are shown by blue arrows, early apoptotic cells by orange arrows, and late apoptotic cells by red arrows, while necrotic cells are indicated by white arrows. Scare bar = 100 µm.

Apoptosis is a type of genetically determined cell death that occurs to maintain the normal physiological process of healthy organisms. Acridine orange/ethidium bromide staining procedure was executed to distinguish live cells and cells undergoing different stages of apoptosis like early apoptotic cells, late apoptotic cells, and deceased cells [41,42]. The morphological alterations, such as the shrinkage of nucleus, DNA condensation, DNA fragmentation, and formed apoptotic bodies of MCF-7 cells, were analyzed under fluorescence microscope following treatment with compound **VI(i)**. As shown in Figure 4, the green fluorescence was emitted by control cells, which possess healthy nuclei and cytoplasm. Early apoptotic cells exhibited condensed nuclei, which exhibits extra bright green fluorescence, and chartreuse fluorescence was radiated from the late apoptotic cells. At a lower concentration (40 µg/mL), both the live and early apoptotic cells were observed, whereas in the case of IC_50_ (60 µg/mL), all cell populations (live, early, and late phase of apoptotic cells) were detected. However, in case of higher concentrations (80 µg/mL) of compound **VI(i)**, apoptosis induction was enhanced, and a greater number of cells were distinguished in the early and late apoptotic phase. The apoptosis-inducing potential of compound **VI(i)** is evidenced by nuclear condensation, DNA fragmentation, and apoptotic body formation, ultimately disturbing cellular integrity of MCF-7 cells. Thus, the current study suggests that compound **VI(i)** can effectively trigger the apoptotic mode of cell death in breast cancer cells in a concentration-dependent manner.

### 2.8. Enhanced Reactive Oxygen Species (ROS) Analysis

**Figure 5:**
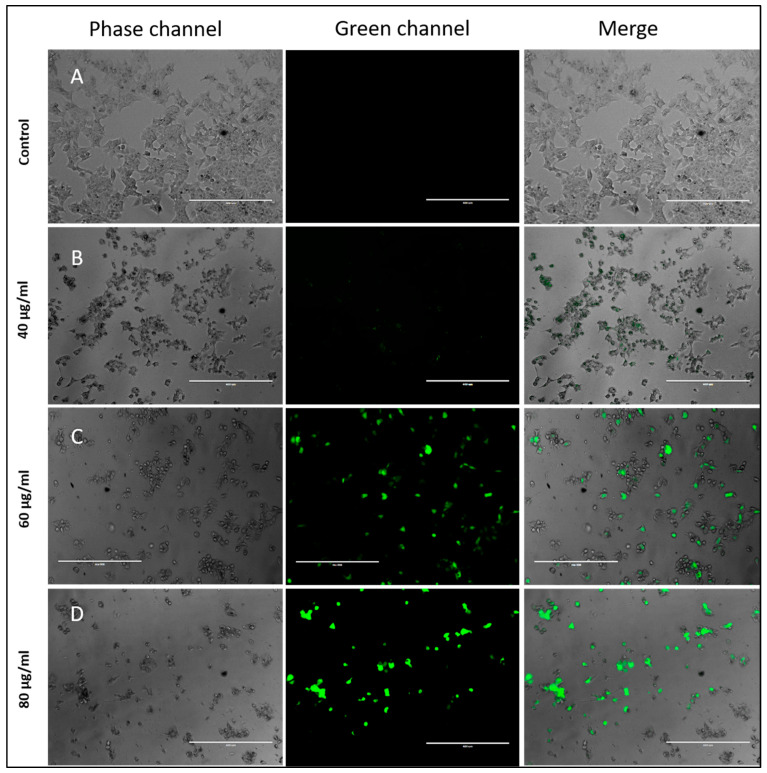
DCFH-DA, a fluorescent dye was utilized to determine the enhanced oxidative stress following treatment with compound **VI(i)**. Phase contrast, green, and merged images of both untreated and compound VI(i) treated MCF-7 cells were shown where, (**A**) depicts control group and (**B**) lower (40 µg/mL), (**C**) IC_50_ (60 µg/mL), and (**D**) higher (80 µg/mL) concentrations treated cells. Scare bar = 400 µm.

Oxidative stress is extensively known for inducing apoptosis via damage to nucleotides, proteins, and lipids. Depending on the concentration of ROS, it can play a binary role. At a lower concentration, it is beneficial for normal physiology, while when its concentration increases, it becomes detrimental to the healthy survival of the organisms due to several consequences [43,44]. In the current study, DCFH-DA dye was utilized to detect the level of ROS after treating MCF-7 cells with compound **VI(i).** An inverted fluorescence microscope was used to observe the result. Figure 5 indicates that after treating MCF-7 cells with compound **VI(i)**, the concentration of ROS was significantly increased in concentration-dependent manner with respect to control cells. The significant elevation in the ROS production possibly causes oxidative stress, leading to cellular damage and triggering programmed cell death. Our findings demonstrate the potential of compound **VI(i)** as a promising anticancer agent against MCF-7 breast cancer cells.

### 2.9. Cell Cycle Analysis

**Figure 6:**
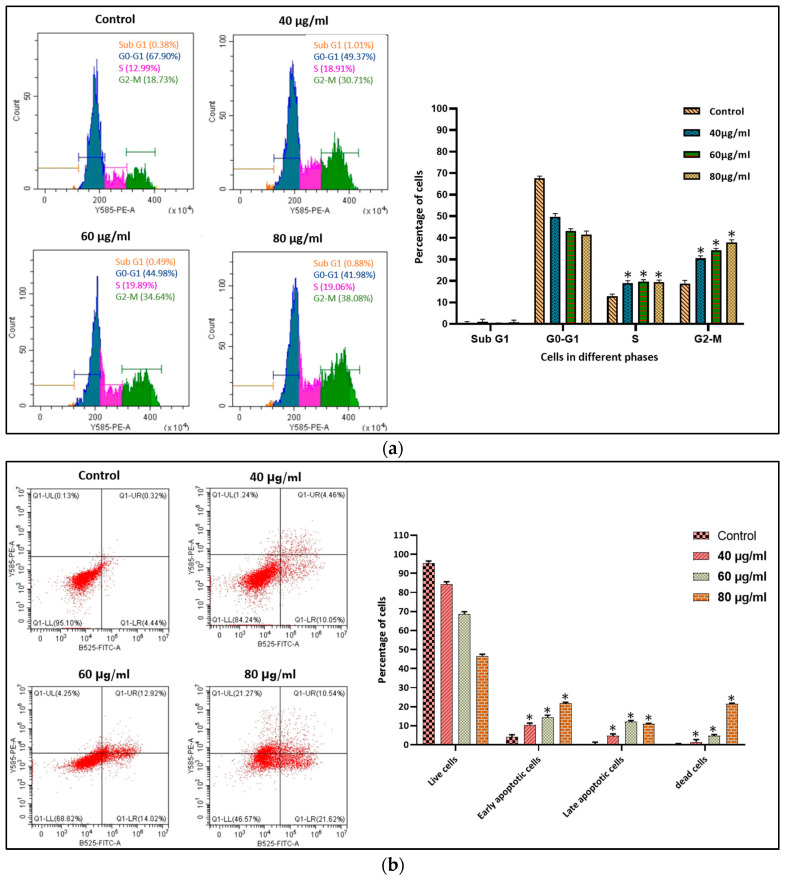
(**a**) Cell cycle exploration of control- and compound **VI(i)**-treated human adenocarcinoma (MCF-7) cells performed via flow cytometry. Propidium iodide dye was used to determine the percentage of cells in different phases of the cell cycle (Sub G1, G1, S and G2/M). Significant differences were considered at *p < 0.05 with respect to control. (**b**) A flow cytometer was used to distinguish different apoptotic cell stages (early apoptotic, late apoptotic, and dead cells) from normal healthy cells in control- and compound **VI(i)**-treated MCF-7 cells. Annexin V/PI was used to quantitatively validate cells in different phases of apoptosis as a percentage. With respect to control, significant differences were considered at * *p* < 0.05.

The effectiveness of the **VI(i)** in inducing cell cycle arrest has been explored using a propidium iodide labeling technique through flow cytometry. Figure 6a illustrates the four different regulatory stages (Sub G1, G1, S, G2/M) of the cell cycle. The MCF-7 cells exposed to the various concentrations (lower, IC_50_, and higher) showed a significant delay at the S and G2/M stages of the cell cycle. The increment in the percentages of arrested cells was seen to be concentration-dependent. Compared to control, the upsurge in cells arrest at the lower concentration was observed to be 5.92% and 11.98% higher in the S and G2/M phases, respectively. Similarly, cells treated with the IC_50_ and higher concentrations show increments of cells arrest of 6.9% and 6.07% in the S phase and 15.91% and 19.35% in G2/M phase, respectively. Therefore, the data obtained indicates that **VI(i)** has the capacity to induce cell cycle arrest at both the S and G2/M stages, and that may have sufficient potency to influence apoptosis.

### 2.10. Apoptosis Assay Through AnnexinV/PI

To validate the apoptotic type of cell death under the influence of compound **VI(i),** quantitatively, annexin V/propidium iodide analysis was carried out using a flow cytometer [45]. Figure 6b demonstrates the obtained data at various concentrations (40 µg/mL, 60 µg/mL, 80 µg/mL) of compound **VI(i)**, and it shows an increment in the percentages of apoptosis in the different treated groups with respect to control. Flow cytometry data revealed that the bottom left quadrant includes the live cells, the upper left quadrant contains the dead/necrotic cells, and the lower quadrant and upper right quadrant contain the early and late phases of the apoptotic cells, respectively. The increment in the cell’s population at the early apoptotic phase at lower, IC_50_, and higher concentrations were observed to be 5.61%, 9.58%, and 17.18%, respectively. Similarly, the increment in the late apoptotic phase of different treated groups (lower, IC_50_, and higher) were observed to be 4.14%, 12.6%, and 10.22%, respectively. The concentration-dependent increase in early and late apoptotic cell populations upon treatment with compound **VI(i)** highlights its ability to trigger programmed cell death, which aligns with one of the key mechanisms of HDAC inhibitors. Therefore, from the present data, it can be concluded that compound **VI(i)** has the potential to affect apoptosis in breast adenocarcinoma (MCF-7) cells.

### 2.11. HDAC–Inhibitory Assay

In vitro cell line studies of some selected compounds with potential anticancer activity were exposed to HDAC1 and HDAC6 isoform inhibition assays [46,47] (Table 2). Among them, **VI(i)** showed exceptional inhibitory efficacy against HDAC1 and HDAC6, serving IC_50_ values of 3.06 and 4.08 µg/mL, respectively. Although the activity of **VI(i)** was smaller than that of SAHA, the compound, like **VI(e)**, also demonstrated notable HDAC1- and HDAC6-inhibitory action. The present study is an effort to examine the impact of 1,2,3-triazole as a cap group in hydroxamic acid-based derivatives with respect to their HDAC1- and HDAC6-inhibitory activity. In a similar study, Kalinin et al. investigated 1,2,3-triazole-capped HDAC inhibitors and found that their IC_50_ values against HDAC1 and HDAC6 isoforms ranged from 1.7 to 42.50 µM, which is higher than what we found [33]. These findings suggest that the triazole cap is a promising pharmacophoric component for HDAC inhibition and prompt additional investigation into isoform specificity and potency enhancement. A similar study, Chen et al., used HeLa nuclear extracts to assess HDAC inhibition and found IC_50_ values ranging from 9.6 to 110 nM. However, direct comparisons of IC_50_ values are complicated due to differences in assay conditions, cell lines, and compound structures. Nonetheless, aforementioned studies clearly indicate that the incorporation of a 1,2,3-triazole moiety as a cap contributes favorably to HDAC–inhibitory activity, particularly for HDAC1 and HDAC6 [32].

### 2.12. Structure-Activity Relationship (SAR) (Figure 7)

The detailed SAR analysis of the synthesized 1,2,3-triazole-based hydroxamide analogs revealed critical structural parameters impacting their anticancer efficacy and HDAC–inhibitory activity. The substitution arrangement on the phenyl ring adjacent to the triazole moiety (cap group) had a significant impact on biological activity. Electron-donating substituents were more effective than electron-withdrawing groups. An optimal modification in compound **VI(i)** was made through *p*-methoxy substitution in a cap region, which demonstrated exceptional activity against MCF-7 cells (IC_50_ = 60 μg/mL and GI_50_ < 10 μg/mL) and remarkable HDAC1/HDAC6 inhibitory potency (IC_50_ = 3.06 and 4.08 μg/mL, respectively). The trend was followed by *p*-hydroxy and *p*-chloro substituents, which also contributed to significant antiproliferative activity. Additionally, *p*-nitro substitution, on the other hand, had modest activity due to its strong electron-drawing character, whereas *p*-methyl and unsubstituted phenyl rings resulted in significantly reduced anticancer activity. The linker optimization revealed that incorporating 6-bromohexanoic acid as the connecting moiety between the triazole ring and hydroxamide provided an optimal anticancer activity. Its six-carbon aliphatic chain offers an ideal flexibility and spatial positioning for effective HDAC binding. However, the presence of aromatic linkers such as 3-(4-bromophenyl)propionic acid significantly reduces the activity due to increased rigidity, which compromises optimal binding with the HDAC enzyme. Hence, **VI(i)**, being the most effective among all hybrids, possessed a *p*-methoxyphenyl clubbed 1,2,3-triazole as a cap and a 6-carbon straight chain as a linker in its pharmacophore. It presented remarkable selectivity for MCF-7 cells over other cancer cell lines, with somewhat minimal toxicity toward normal HEK-293 cells but still indicating a favorable therapeutic window. Also, the electron-donating methoxy group favors binding affinity through improved π-π stacking interactions, with aromatic residues present on the rim of the HDAC binding pocket. At the same time, an appropriate linker length (6-carbon straight chain) smoothly enables the hydroxamide group of the molecule to reach inside the enzyme and interact with Zn^2+^ for the purposes of inhibition. **VI(i)** emerged as a lead candidate for dual HDAC1/HDAC6 inhibition. These findings establish an unambiguous design criterion for the development of next-generation HDAC inhibitors.

### 2.13. Molecular Docking

**Figure 8:**
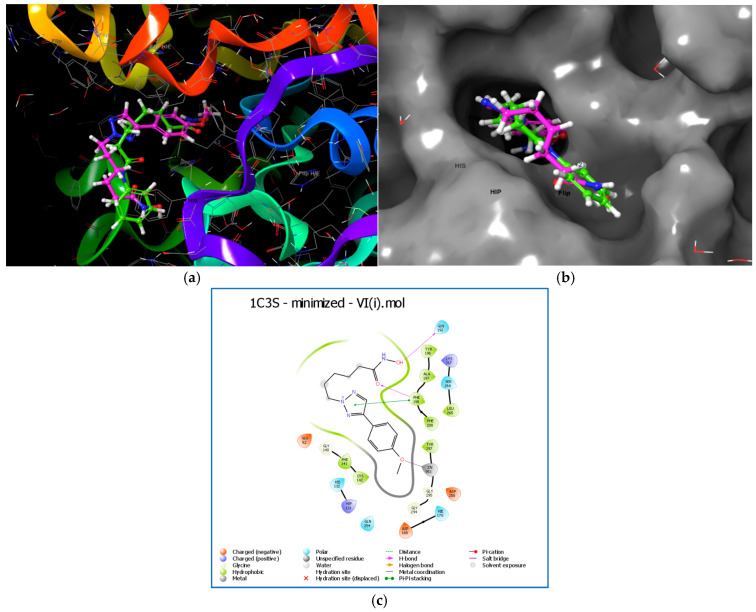
(**a**) XP docking pose of compound **VI(i)** (pink), superimposed with reference HDAC inhibitor **SAHA** (green). (**b**) **VI(i)** and SAHA entering into the catalytic pocket of HDAC1 protein (1C3S). (**c**) Two-dimensional ligand interaction diagram of **VI(i)**, interacting with different amino acids of HDAC1 protein (1C3S).

**Figure 9:**
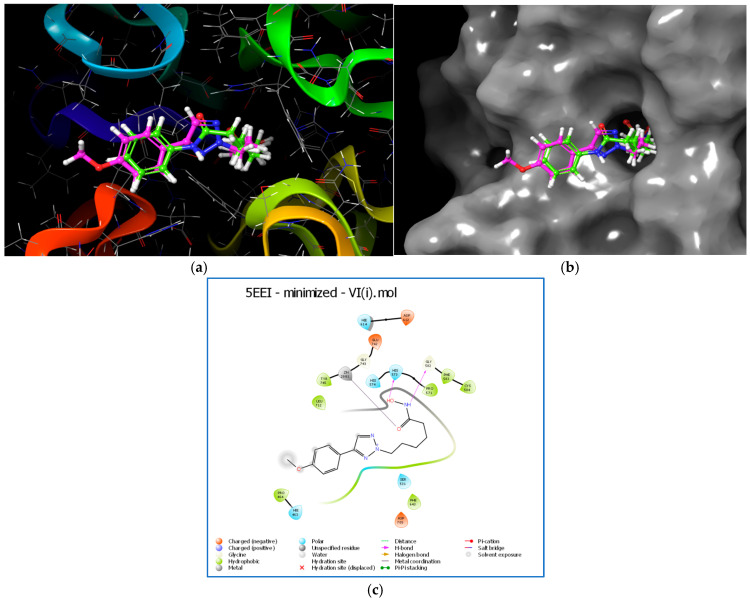
(**a**) XP docking pose of compound **VI(i)** (pink), superimposed with reference HDAC inhibitor **SAHA** (green). (**b**) **VI(i)** and SAHA entering into the catalytic pocket of HDAC protein (4LXZ). (**c**) Two-dimensional ligand interaction diagram of **VI(i)**, interacting with different amino acids of HDAC6 protein (5EEI).

The extra precision (XP) docking scores (in kcal/mol) of synthesized compounds (Table 3) were assessed against HDAC1 (PDB: 1C3S) and HDAC6 (PDB: 5EEI) proteins. The obtained docking scores provided insights into binding affinity, with more negative values corresponding to a more stable binding interaction [48,49,50,51,52]. **VI(i)**, **VI(j)**, and **VI(m)** showed the most favorable docking scores against HDAC1, all being below −10 kcal/mol (−10.691, −10.482, and −10.047 kcal/mol, respectively). Interestingly, several compounds also exhibited preferable binding with HDAC6 like **VI(i)**, **VI(j)**, **VI(o)**, **VI(m)**, and **VI(n)**, with docking scores of −11.373, −10.358, −10.349, −10.201, and −10.073 kcal/mol, respectively. This data indicated that these synthesized derivatives, especially **VI(i)** and **VI(j)**, could possibly exhibit dual HDAC1/HDAC6 binding affinities. The docking pose visualization of compound **VI(i)** with HDAC proteins are illustrated in Figure 8 and Figure 9.

### 2.14. MD Simulation

Molecular docking has a significant limitation by considering the receptor (protein) as a non-flexible, rigid body, which can be overcome through the MD simulation feature. MD simulation efficiently assesses the flexibility and stability of the protein–ligand complexes, thus offering a greater understanding of the ligand’s binding mechanism and behavior in a dynamic environment [53,54,55,56,57,58]. The docking posture of **VI(i)** in the optimized HDAC 1 [1C3S] and HDAC6 [5EEI] proteins were subjected to simulation for 100 ns [59].

### 2.15. Trajectory Analysis for **VI(i)**-1C3S Complex (Figure 10)

The protein chain A was retained during the simulation, with a total number of residues as 372 (5968 as total atoms and 2994 as heavy atoms) for 1C3S.

RMSD: The protein’s backbone (Cα) exhibits relatively stable RMSD values around 1.5–2.0 Å, indicating minimal structural deviation. The ligand’s RMSD fluctuates more, suggesting flexible binding behavior. Initially, the ligand stabilizes around 2 Å but later oscillates between 6 and 8 Å, possibly indicating transient binding or multiple conformational states. The fluctuations suggest dynamic interactions between the ligand and the protein. Overall, the trajectory implies that while the protein remains stable, the ligand undergoes significant movement, hinting at possible conformational rearrangements.

RMSF of protein (1C3S): The RMSF values range from approximately 0.345 Å to 3.246 Å

RMSF of ligand **[VI(i)]**: **VI(i)** displayed RMSF in the range of 1.080–3.356 Å with regard to protein.

Ligand properties: The RMSD of **VI(i)** ranged between 0.777 to 3.256 Å with regard to its reference (t = 0) conformation. The radius of gyration (rGyr) was within the range of 3.907 to 5.554. It ensured an alteration in compactness of **VI(i)** with 1C3S. Intra-molecular H-bonding was present with three different residues. The Mol SA, SASA, and PSA ranged from 304.92 to 326.542 Å, from 53.884 to 171.116 Å, and from 132.153 to 183.972 Å, respectively.

1C3S-**[VI(i)]** contacts: The compound **VI(i)** interacted with ASN20 (∼52%), TYR91 (∼10%), GLU92 (∼9%), LYS19 (∼8%), and HIS21, GLY90, VAL95, TYR196, TYR264, LEU265, LYS267, and TYR297 (<5%) through hydrogen bonds. It also formed strong hydrophobic interaction with PHE141 (∼35%), PHE198 (∼15%), TYR297 (∼20%), and PRO18, HIS21, PRO22, LEU23, TYR91, PRO94, PHE200, and LEU265 (<5%). However, **VI(i)** failed to established strong ionic interactions with amino residues, but the water bridge bonding was mainly formed with ASN20 (∼15%), GLY90 (∼10%), GLU92 (∼10%), VAL95 (∼15%), and many other amino acids (<5%).

### 2.16. Trajectory Analysis for **VI(i)-**5EEI Complex (Figure 11)

The A and B chains of protein 5EEI were kept for simulation. It carried 714 total residues, 11,010 total atoms, and 5580 heavy atoms.

RMSD: The RMSD trajectory of protein 5EEI and ligand VI(i) over 100 ns reveals significant structural deviations. The protein’s RMSD gradually increases, stabilizing around 6–7 Å after 60 ns, indicating notable conformational changes. The ligand’s RMSD remains below 5 Å for most of the simulation but exhibits a sharp increase beyond 15 Å near 90 ns, suggesting partial or complete dissociation. This fluctuation indicates weak ligand stability and possible unbinding from the protein. The overall trend suggests that while the protein undergoes structural adaptation, the ligand initially interacts stably but later loses its binding conformation, leading to high fluctuations.

RMSF of protein (5EEI): The RMSF values range from approximately 0.538 Å to 5.475 Å, indicating varying levels of motion across residues. Most residues exhibit moderate fluctuations (~1.5–3.5 Å), suggesting overall stability. However, specific regions, particularly around residues 74, 685, and 686, show peaks above 5.0 Å, indicating high flexibility, likely corresponding to loop regions or terminal ends. These fluctuations suggest dynamic regions that may influence ligand binding or structural adaptability. Conversely, stable regions with lower RMSF indicate well-folded, rigid domains critical for function.

RMSF of ligand **[VI(i)]**: **VI(i)** displayed RMSF in the range of 5.033–8.747 Å with regard to protein.

Ligand properties: The RMSD of **VI(i)** ranged between 0.668 to 2.955 Å with regard to its reference (t = 0) conformation. The radius of gyration (rGyr) was within the range of 3.582 to 5.793. This ensured an alteration in compactness of **VI(i)** with 5EEI. Intra-molecular H-bonding was present. The Mol SA, SASA, and PSA ranged from 293.058 to 327.952 Å, from 115.733 to 606.414 Å, and from 115.497 to 179.435 Å, respectively.

5EEI-[VI(i)] contacts: Compound **VI(i)** showed prominent hydrogen bonding with ASP612 (∼70%), HIS574 (∼60%), HIS614 (∼40%), and TYR745 (∼20%) residues of 5EEI. It also participated in hydrophobic interaction with PHE642 (∼40%), PHE643 (∼70%), PHE583 (∼20%), HIS614 (∼20%), TYR745 (∼20%), TYR745 (∼15%), and other residues. In addition, **VI(i)** established strong ionic interactions with ASP612 (∼60%), ASP705 (∼50%), and TYR745 (<20%). Water bridging was formed with TYR745 (∼70%), HIS614 (∼10%), and ASP612 and HIS574 amino acids (<10%).

### 2.17. MM-GBSA Analysis

The Prime program of the Schrodinger suite calculated the MM-GBSA for **VI(i)** with 1C3S and 5EEI proteins. Post simulation, the MM/GBSA binding free energy of docked **1C3S-VI(i)** and **5EEI-VI(i)** complexes were determined (Table 4). The binding free energy is a key indicator of ligand stability within the binding pocket. The binding energy decreased during the span of simulation; this suggested an increase in the binding affinity over time. The stronger electrostatic stabilization and improved solvation energy in the 1C3S complex suggested that it forms more favorable interactions with the surrounding environment compared to 5EEI. Additionally, the 1C3S complex benefited from stronger vdW and hydrophobic interactions, contributing to its improved stability. The hydrogen bonding decreased slightly for both complexes, which may indicate minor fluctuations, but it does not significantly impact the overall stability. Packing interaction decreased for 1C3S but remained negligible for 5EEI. The total complex energy slightly decreased for 1C3S-**VI(i)** (−11,968.88 to −11,986.90 kcal/mol), suggesting stabilization. But for 5EEI-**VI(i)**, it increased (−23,399.1 to −23,378.5 kcal/mol), indicating a destabilization trend.

The result revealed that the 1C3S-**VI(i)** complex showed increased stability over the 100 ns simulation, with enhanced binding affinity, stronger vdW and hydrophobic interactions, and better solvation energy. Conversely, the 5EEI-**VI(i)** complex lost stability, with increased columbic repulsion and weakened van der Waals and hydrophobic interactions, leading to weaker ligand binding. Both appeared to be promising candidates for further drug development; nevertheless, 5EEI-**VI(i)** might require certain structural modifications to improve binding stability compared to 1C3S-**VI(i)**.

### 2.18. ADME and Toxicity Evaluation

The ADME properties of effective clinical candidates make up their pharmacokinetic profile. These properties are essential to evaluate the pharmacodynamic effect of the molecules; hence, fulfilling the criteria’s of ADME test is a requirement for these candidates to be classified as drugs. Solubility, lipophilicity, permeability, integrity, stability, etc., are some essential descriptors that determine the physicochemical properties. The ADME analysis was performed on the synthesized molecules using the QikProp module of Maestro (Table S1). These compounds demonstrated drug-like behavior consistent with their ADME characteristics by passing “the rule of 5” and other pharmacokinetic investigations with values within acceptable limits.

The toxicological activity of compounds showed a relationship with their chemical framework, which can be predicted with the help of in silico toxicity prediction software “ProTox 3.0”. Various quantitative (LD_50_) and qualitative (toxic or non-toxic) parameters were considered, as shown in Table S1 (Supplementary Materials).

### 2.19. Energy Calculation of Molecular Orbitals Through DFT

**Figure 12:**
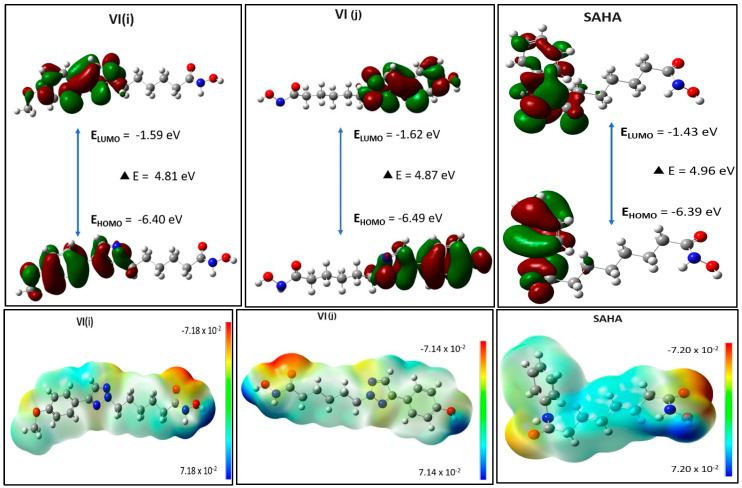
The representation of HOMO and LUMO, along with Electrostatic Potential (ESP) maps of the top two best docked compounds, **VI(i)** and **VI(j)**, and the marketed drug, SAHA, are presented here. The molecular orbital wave function displays positive (red) and negative (green) phase distributions. The HOMO, which represents electron donor regions, determines the ionization potentials, while the LUMO, representing electron acceptor regions, determines the electron affinity. The ESP illustrates the electron-rich (red) and electron-poor (blue) regions.

The DFT technique finds its basis in quantum mechanics, which accurately describes the electronic and structural attributes of any given compound. The orbital energy calculations were employed for the top two best-docked compounds, **VI(i)** and **VI(j)**, as well as the marketed drug **SAHA**, to understand their electronic distributions. This understanding of electronic distributions may shed some light on the ligand–protein interactions and binding mechanisms of these compounds. Differential electrostatic potentials furnish an understanding of regions of varying electron density. In the electronic molecular wave function, the red and green distributions show the positive and negative phases of the wave function, respectively. The HOMO and LUMO locations in a ligand are important for inducing an interaction with any of its potential receptors. There exists an interactive relationship between the ligand HOMO and the receptor LUMO. An increase in ligand HOMO energy reduces the energy gap with the LUMO of the receptor, thereby enhancing binding; conversely, a decrease in the energy of ligand LUMO is also likely to enhance binding [60]. An Electrostatic Potential (ESP) map provides an overview of a ligand’s polarity. On comparing the total energy of all three compounds, SAHA (−23,962.34 eV) showed less negative energy compared to **VI(i)** (−27,978.74 eV) and **VI(j)** (−26,909.72 eV). The compound **VI(i)** exhibited a smaller energy gap (E_HOMO_-E_LUMO_) of 4.81 eV, thus showing more reactivity but less stability than SAHA, along with a greater energy band gap of 4.96 eV (Figure 12). Moreover, the negative nature of the HOMO and the LUMO in all the compounds suggested the stability of the complexes with receptor protein. DFT also calculated the dipole moment, showing the hydrogen bonding ability of the listed compound. Here, SAHA (2.93 Debye) has a lower dipole moment then **VI(j)** and **VI(i)**, which have respective value of 6.49 and 5.19 Debye, indicating better agreement with the docking result (Table 5).

The computation of color-coded Molecular Electrostatic Potential (MEP) provides a more in-depth interpretation of the electrostatic potential of the compounds. The red color indicates the electronegative area acting as a hydrogen bond acceptor, the blue color shows the electropositive area functioning as a hydrogen bond donor, and the yellow and green colors symbolize the neutral zones that are allowed to participate in hydrophobic interactions (Figure 5).

The DFT and MEP analyses also strengthened our understanding on the observed biological activity of compound **VI(i)**. A relatively low HOMO–LUMO energy gap of **VI(i)** suggested high chemical reactivity and a strong tendency to participate in electronic interactions with biomolecular HDAC protein. The HOMO was primarily localized over the hydroxamate and triazole moieties, indicating its role in electron donation, whereas the LUMO was distributed over the aliphatic linker, pointing towards favorable electron-accepting capabilities. These electronic characteristics align with the strong binding affinity shown by **VI(i)** toward HDAC1 and HDAC6 isoforms, as confirmed by molecular docking and MD simulations. Furthermore, the MEP surface of **VI(i)** exhibited a balanced distribution of positive and negative electrostatic potential regions, particularly around the hydroxamate zinc-binding group and aromatic cap. This distribution supports the formation of stable hydrogen bonds and electrostatic interactions with key amino acid residues in the HDAC active site. Collectively, these analyses provide a theoretical basis by which to validate the biological findings of selective HDAC inhibition, ROS-mediated apoptosis induction, and potent cytotoxicity against MCF-7 cells. These results confirmed that the electronic structure and surface potential of **VI(i)** has a direct link with its biological efficacy.

## 3. Materials and Methods

### 3.1. Materials

Every substance employed in the synthesis and analysis was of laboratory grade and analytical quality. The method of open capillary was employed with melting point equipment to estimate the melting temperatures, which were left uncorrected. TLC was used to assess the compounds’ purity by means of pre-coated silica gel–aluminium TLC plates (Merck, Darmstadt, Germany) as a stationary phase, with different solvent systems and iodine vapors and UV light (254 nm) as detecting agent. The IR spectrum was recorded on a SHIMADZU (Kyoto, Japan), IRPrestige-21, using KBr pellets and a Perkin Elmer Spectrum Two FT-IR spectrophotometer. Tetramethylsilane (TMS) (Waltham, MA, USA) was used as the internal standard, and Bruker AVANCE III 500 MHz (AVH D 500) (Billerica, MA, USA) and JEOL JNM-ECZR 600 MHz (Tokyo, Japan) spectrometers were used to record the ^1^H NMR spectra of the synthesized compounds. Chemical shifts were restrained in ppm, δ. The compounds showed solubility in CDCl_3_, with δ ranging from 7.06 to 7.35 in ^1^H NMR on a Bruker Avance-II 400 MHz NMR spectrometer in ^13^C NMR. The mass spectra were recorded on a Shimadzu LC-MS 8040 system.

### 3.2. Synthesis of Target Compounds

#### 3.2.1. General Procedure for Synthesis of Substituted Acrylonitrile **III(a–g)**

Equimolar quantities of acetonitrile (0.04 mol) and substituted benzaldehyde (0.04 mol) were dissolved in a 5% sodium methoxide–methanol solution that was stirred for 4–6 h at 40–45 °C. The resulting solution was neutralized to obtain a final precipitate, which was filtered and washed with distilled water [37]. The yield of the final compounds was in the range of 48.92 to 63.31% after recrystallization with methanol.

#### 3.2.2. General Procedure for Synthesis of Substituted 1,2,3-Triazole **IV(a–g)**

The mixture of the substituted acrylonitrile (0.005 mol), NaN_3_ (0.015 mol), and NH_4_Cl (0.015 mol) in DMF: H_2_O (30 mL:3 mL) was refluxed for 12–14 h. The TLC monitored the progress of the reaction. When the starting material had completely disappeared, we added some chilled water and stirred the mixture for 20–25 min in cold water. The precipitated crude product was separated and recrystallized in methanol or ethanol, yielding the requisite final products. However, ethyl acetate was used to extract the product in the absence of precipitation. The organic extract was thoroughly rinsed with water, and the resultant organic fraction was allowed to dry on a rotary evaporator to obtain the required product [37].

#### 3.2.3. General Procedure for Synthesis of Substituted 1,2,3-Triazole-Based Hydroxamic Acid Analogs **V(a–o)**

The substituted 1,2,3-triazole (4 mmol), sodium bicarbonate (4 mmol), and substituted acid (4 mmol) was dissolved in DMF (30 mL). The mixture was stirred for 6–7 h at 65–80 °C. It was transferred into another flask containing distilled water (50–70 mL) with some crushed ice cubes. The precipitate was strained and recrystallized with a suitable solvent, yielding the desired product [61,62].

#### 3.2.4. General Procedure for Synthesis of Substituted 1,2,3-Triazole-Based Hydroxamide Analogs **V(a–o)**

The substituted 1,2,3-triazole-based hydroxamic acid (2.0 mmol) was dissolved in anhydrous THF (30–40 mL) in an ice bath. Simultaneously, Et_3_N (2.4 mmol) and isobutyl chloroformate (2.4 mmol) were poured into it in sequence. The resultant blend was swirled for 20–25 min at 0 °C. In another beaker, a solution of NH_2_OH.HCl (8.0 mmol) and Et_3_N (8.0 mmol) in anhydrous methanol (10–12 mL) was prepared and mixed with the solution in ice bath. The entire mixture stirred for 20–24 h at RT. After eliminating the solvents post reaction completion, the subsequent solid was initially dissolved in ethyl acetate, followed by a series of washes with HCl (1 M), distilled water, and saturated NaCl solution to eliminate any impurities. The final product was recrystallized by ethyl acetate.

### 3.3. Analytical Data for the Compounds

#### 3.3.1. 3-(4-Methoxyphenyl)acrylonitrile, **III(a)**

Pale yellow crystals. Yield 71.23%, m.p: 97–99 °C, R_f_: 0.67 (CH_3_OH:CHCl_3_: 4:1). IR (cm^−1^): 1028.06 and 1259.52 (C-O str. aryl alkyl ether); 1521.10 and 1610.53 (aromatic C=C str.), 2563.40 (-CN, m, nitrile), 2895.15 (C h str. alkane). ^1^H NMR (500 MHz, CDCl_3_) δ (ppm): 3.78 (m, 3H), 4.88 (s, 2H),7.04–6.89 (m, 2H), 7.28 (d, *J* = 2.1 Hz, 1H), 7.86 (d, *J* = 4.2 Hz, 1H), 8.08 (d, *J* = 4.2 Hz, 1H). ^13^C-NMR (100 MHz, CDCl_3_) δ (ppm): 56.21, 93.93, 113.36, 115.06, 118.80, 122.08, 129.76, 148.00, 148.84, 149.45. LC-MS (ESI): *m/z* calcd for C_10_H_9_NO [M+H]^+^: 160.075; found 160.

#### 3.3.2. 4-(4-Methoxyphenyl)-2H-1,2,3-triazole, **IV(a)**

Light brown solid. Yield 68.37%, m.p: 122–124 °C, R_f_: 0.54 (ethyl acetate–*n*-hexane: 4:1). IR (cm^−1^): 1026.13 and 1261.45 (C-O str. aryl alkyl ether), 3099.69 (aromatic =C h str.). ^1^H NMR (500 MHz, CDCl_3_) δ (ppm): 3.80 (t, *J* = 7.8 Hz, 2H), 4.48 (s,1H), 6.95 (d, *J* = 6.3 Hz, 1H), 7.86–7.02 (m,2H), 8.08 (d, *J* = 4.2 Hz, 1H). ^13^C-NMR (100 MHz, CDCl_3_) δ (ppm): 16.53, 55.60, 113.10, 124.75, 125.24, 126.43, 128.19, 128.46, 144.67. LC-MS (ESI): *m/z* calcd for C_9_H_9_N_3_O [M+H]^+^: 176.081; found 176.

#### 3.3.3. 4-(4-(4-(4-Methoxyphenyl)-2H-1,2,3-triazol-2-yl)phenyl)-4-oxobutanoic Acid **V(a)**

Fine needle shaped yellowish-brown crystals. Yield 52.17%, m.p: 165–167 °C, R_f_: 0.65 (ethyl acetate–*n*-hexane: 4:1). IR (cm^−1^): 1026.13 and 1261.45 (C-O str. aryl alkyl ether), 1427.32 (CH_2_ bend methylene group), 1707.89 (C=O str. carboxylic acid), 1745.58 (C=O str. ketone), 3365.84 (O h str., broad peak). ^1^H NMR (500 MHz, CDCl_3_) δ (ppm): 2.69 (s, 1H), 3.83 (t, *J* = 7.8 Hz, 1H), 3.90 (s, 1H), 7.09–7.05 (m, 1H), 7.25 (d, *J* = 6.3 Hz, 1H), 7.62–7.43 (m, 1H), 7.94 (s, 1H). ^13^C-NMR (100 MHz, CDCl_3_) δ (ppm): 16.53, 28.17, 33.82, 55.60, 113.29, 115.72, 120.83, 123.33, 125.26, 126.58, 130.97, 133.39, 134.28, 149.22, 149.73, 160.90, 174.82, 197.90. LC-MS (ESI): *m/z* calcd for C_19_H_17_N_3_O_4_ [M+H]^+^: 352.129; found 352.

#### 3.3.4. N-Hydroxy-4-(4-(4-(4-methoxyphenyl)-2H-1,2,3-triazol-2-yl)phenyl)-4-oxobutanamide **VI(a)**

Whitish-yellow crystals. Yield 48.92%, m.p: 192–194 °C, R_f_: 0.62 (CHCl_3_–cyclohexane: 3:2). IR (cm^−1^): 3301.94 (O-H); 3286.36 (N-H, sec. amide), 1701.34 (C=O); 1679.95 (C=O, amide), 1258.51 (C-O, ether). ^1^H NMR (500 MHz, CDCl_3_) δ (ppm): 1.27(s,1H), 1.42 (s, 1H), 3.15 (s, 2H), 3.85 (d, J = 8.4 Hz, 1H), 7.94–7.05 (m, 2H), 8.07 (d, J = 4.2 Hz, *2H*), 10.80 (s, 2H). ^13^C-NMR (100 MHz, CDCl_3_) δ (ppm): 19.15, 44.61, 55.31, 68.10, 113.91, 115.85, 120.49, 120.51, 125.82, 129.41, 130.60, 134.31, 134.88, 140.23, 143.96, 148.38, 160.75, 170.55, 197.94. LC-MS (ESI): m/z calcd for C_19_H_18_N_4_O_4_ [M+H]^+^: 367.140; found 367.

#### 3.3.5. N-Hydroxy-4-(4-(4-(4-nitrophenyl)-2H-1,2,3-triazol-2-yl)phenyl)-4-oxobutanamide **VI(b)**

Dark orange powder. Yield 51.60%, m.p: 220–222 °C, R_f_: 0.69 (CHCl_3_–cyclohexane: 3:2). IR (cm^−1^): 3339.23 (O-H); 3305.32 (N-H, sec. amide), 1711.78 (C=O), 1683.61 (C=O, amide), 1338.25 (N=O). ^1^H NMR (500 MHz, CDCl_3_) δ (ppm): 1.84 (m, 2H), 2.45 (t, *J* = 8.8 Hz, 1H), 3.60 (t, *J* = 8.8 Hz, 1H), 7.28–7.05 (m, 1H), 7.94–7.53 (m, 2H), 8.25–8.02 (m, 2H), 8.90 (d, *J* = 3.5 Hz, 1H), 11.39 (s, 1H). ^13^C-NMR (100 MHz, CDCl_3_) δ (ppm): 38.71, 81.31, 120.85, 120.87, 125.02, 125.08, 127.27, 130.89, 133.61, 134.83, 135.40, 143.91, 146.60, 149.84, 165.80, 196.99. LC-MS (ESI): m/z calcd for C_18_H_15_N_5_O_5_ [M+H]^+^: 382.114; found 382.

#### 3.3.6. 4-(4-(4-(4-Chlorophenyl)-2H-1,2,3-triazol-2-yl)phenyl)-N-hydroxy-4-oxobutanamide **VI(c)**

Pale whitish solid. Yield 56.84%, m.p: 205–207 °C, R_f_: 0.73 (ethyl acetate–benzene: 3:2). IR (cm^−1^): 3523.16 (O-H), 3351.95 (N-H, sec. amide), 1741.25 (C=O), 1670.89 (C=O, amide), 630.92 (C-Cl). ^1^H NMR (500 MHz, CDCl_3_) δ (ppm): 1.23 (m, 2H), 2.01 (t, *J* = 8.4 Hz, 2H), 3.13 (s, 2H), 7.28 (s,2H), 7.38 (s, 2H), 7.49–7.44 (m, 2H), 7.73 (d, J = 4.2 Hz, 1H), 7.83 (d, J = 12.6 Hz, 1H), 8.10–8.03 (m, 2H), 8.44 (s, 1H), 11.52 (s, 2H). ^13^C-NMR (100 MHz, CDCl_3_) δ (ppm): 39.92, 54.67, 120.71, 120.75, 127.90, 129.66, 129.70, 130.47, 131.50, 132.87, 133.60, 135.25, 143.19, 149.81, 170.00, 191.69. LC-MS (ESI): m/z calcd for C_18_H_15_ClN_4_O_3_ [M+H]^+^: 371.090; found 371.

#### 3.3.7. N-Hydroxy-4-oxo-4-(4-(4-(*p*-tolyl)-2H-1,2,3-triazol-2-yl)phenyl)butanamide **VI(d)**

Light brown-white powder. Yield 58.02%, m.p: 186–188 °C, R_f_: 0.60 (CHCl_3_:*n*–hexane: 4:1). IR (cm^−1^): 3352.36 (O-H), 3301.82 (N-H, sec. amide), 3048.17 (C-H), 1697.17 (C=O), 1600.92 (C=O, amide). ^1^H NMR (500 MHz, CDCl_3_) δ (ppm): 1.35–1.28(m, 2H), 2.70 (t, *J* = 8.8 Hz, 1H), 3.16 (t, *J* = 8.4 Hz, 1H), 7.29–7.22 (m, 2H), 7.66–7.57 (m, 2H), 8.01–7.99(m, 1H), 8.20 (d, *J* = 3.2 Hz, 1H), 10.69 (d, *J* = 3.2 Hz, 1H). ^13^C-NMR (100 MHz, CDCl_3_) δ (ppm): 16.51, 21.26, 37.17, 43.48, 120.52, 120.54, 126.43, 126.47, 129.90, 130.45, 130.50, 130.52, 133.58, 135.47, 137.99, 143.95, 149.61, 174.72, 198.82. LC-MS (ESI): m/z calcd for C_19_H_18_N_4_O_3_ [M+H]^+^: 351.145; found 351.

#### 3.3.8. N-Hydroxy-4-(4-(4-(4-hydroxyphenyl)-2H-1,2,3-triazol-2-yl)phenyl)-4-oxobutanamide **VI(e)**

Brown solid. Yield 48.10%, m.p: 202–204 °C, R_f_: 0.69 (ethyl acetate–*n*-hexane: 4:1). IR (cm^−1^): 3401.54 (O-H), 3390.18 (N-H, sec. amide), 1682.70 (C=O). ^1^H NMR (600 MHz, CDCl_3_) δ (ppm): 3.30 (dd, *J* = 17.6, 7.0 Hz, 1H), 3.55 (dd, *J* = 17.6, 7.0 Hz, 1H), 6.37 (dd, *J* = 1.8, 0.9 Hz, 1H), 6.56 (d, *J* = 2.1 Hz, 1H), 6.74 (dd, *J* = 8.5, 2.1 Hz, 1H), 7.37 (s, 1H), 7.65–7.62 (m, 2H), 7.91–7.89 (m, 2H), 8.07 (d, *J* = 1.8 Hz, 1H), 9.24 (d, *J* = 3.5 Hz, 1H), 9.47 (d, *J* = 3.3 Hz, 1H). ^13^C-NMR (100 MHz, CDCl_3_) δ (ppm): 31.09, 36.17, 107.40, 116.53, 116.54, 125.10, 125.33, 127.87, 130.31, 130.35, 135.74, 144.16, 150.79, 159.56, 163.74, 170.93. LC-MS (ESI): m/z calcd for C_18_H_16_N_4_O_4_ [M+H]^+^: 352.124; found 352.

#### 3.3.9. 4-(4-(4-(4-Fluorophenyl)-2H-1,2,3-triazol-2-yl)phenyl)-N-hydroxy-4-oxobutanamide **VI(f)**

Fluffy white powder. Yield 51.47%, m.p: 188–191 °C, R_f_: 0.70 (ethyl acetate–cyclohexane: 4:1). IR (cm^−1^): 3310.65 (O-H), 3293.21 (N-H, sec. amide), 1722.63 (C=O), 1674.89 (C=O, amide), 1220.19 (C-F). ^1^H NMR (600 MHz, CDCl_3_) δ (ppm): 2.69 (dd, *J* = 16.9, 7.9 Hz, 1H), 2.95 (dd, *J* = 16.8, 8.0 Hz, 1H), 6.26 (dd, *J* = 1.9, 0.9 Hz, 1H), 7.15–7.11 (m, 2H), 7.74–7.67 (m, 4H), 7.87–7.85 (m, 2H), 8.09 (d, *J* = 1.8 Hz, 1H), 9.30 (d, *J* = 3.1 Hz, 1H), 9.69 (d, *J* = 3.1 Hz, 1H). ^13^C-NMR (100 MHz, CDCl_3_) δ (ppm): 39.17, 72.21, 107.41, 116.20, 116.21, 116.38, 116.39, 125.32, 127.98, 128.96, 128.99, 130.99, 130.60, 130.66, 133.31, 144.46, 150.77, 162.19, 163.55, 164.16, 170.28. LC-MS (ESI): m/z calcd for C_18_H_15_FN_4_O_3_ [M+H]^+^: 355.120; found 355.

#### 3.3.10. 4-(4-(4-(3,4-Dimethoxyphenyl)-2H-1,2,3-triazol-2-yl)phenyl)-N-hydroxy-4-oxobutanamide **VI(g)**

Yellowish orange powder. Yield 52.60%, m.p: 181–183 °C, R_f_: 0.75 (CHCl_3_–benzene: 2:3). IR (cm^−1^): 3279.99 (O-H), 1676.36 (C=O), 1297.84 and 1254.36 (C-O, ether). ^1^H NMR (600 MHz, CDCl_3_) δ (ppm): 2.70 (dd, *J* = 16.8, 7.6 Hz, 1H), 2.95 (dd, *J* = 16.8, 7.5 Hz, 1H), 3.37 (d, *J* = 1.4 Hz, 3H), 3.85 (d, *J* = 7.1 Hz, 5H), 6.26 (d, *J* = 7.9 Hz, 1H), 7.38 (dd, *J* = 7.9, 2.0 Hz, 1H), 7.69–7.67 (m, 2H), 7.88–7.86 (m, 2H), 9.04 (d, *J* = 3.1 Hz, 1H), 9.73 (d, *J* = 3.3 Hz, 1H). ^13^C-NMR (400 MHz, CDCl_3_) δ (ppm): 55.94, 55.98, 107.41, 112.04, 112.54, 121.54, 125.02, 125.12, 125.13, 127.88, 135.77, 144.16, 148.96, 151.76, 152.14, 163.40, 170.97, 199.10. LC-MS (ESI): m/z calcd for C_20_H_20_N_4_O_5_ [M+H]^+^: 397.150; found 397.

#### 3.3.11. 6-(4-(4-Chlorophenyl)-2H-1,2,3-triazol-2-yl)-N-hydroxyhexanamide **VI(h)**

Yellowish-brown semi-solid. Yield 57.41%, R_f_: 0.54 (ethyl acetate–hexane: 3:2). IR (cm^−1^): 3243.40 (O-H), 1646.62 (C=O, amide), 1594.93 and 1472.39 (C=C, aromatic ring), 1444.26 (CH2 bend), 762.46 (C-Cl). ^1^H NMR (600 MHz, CDCl_3_) δ (ppm): 1.72–1.59 (m, 2H), 1.91–1.84 (m, 2H), 1.94 (m, 1H), 4.08 (dt, *J* = 14.1, 6.2 Hz, 1H), 7.42–7.40 (m, 2H), 7.80–7.77 (m, 2H), 8.08 (s, 1H), 9.44 (d, *J* = 3.5 Hz, 1H), 9.51 (d, *J* = 3.5 Hz, 1H). ^13^C-NMR (100 MHz, CDCl_3_) δ (ppm): 21.23, 29.85, 34.27, 39.71, 53.55, 67.88, 128.19, 129.66, 130.66, 130.68, 130.84, 135.24, 149.09, 171.64. LC-MS (ESI): m/z calcd for C1_4_H_17_ClN_4_O_2_ [M+H]^+^: 309.111; found 310.

#### 3.3.12. N-Hydroxy-6-(4-(4-methoxyphenyl)-2H-1,2,3-triazol-2-yl)hexanamide **VI(i)**

Brownish-black semi-solid. Yield 57.91%, R_f_: 0.57 (ethyl acetate–hexane: 3:2). IR (cm^−1^): 3366.59 (O-H), 1682.35 (C=O, amide), 1645.25 and 1473.67 (C=C, aromatic ring), 1449.31 (CH2 bend), 1173.72 (C-O, ether). ^1^H NMR (600 MHz, CDCl_3_) δ (ppm): 1.78–1.51 (m, 4H), 2.09 (s, 1H), 2.31–2.25 (m, 1H), 3.48–3.45 (m, 1H), 3.83 (s, 2H), 4.17 (dd, *J* = 14.5, 3.3 Hz, 1H), 6.85 (ddd, *J* = 7.9, 2.1, 1.1 Hz, 1H), 7.36–7.31 (m, 2H), 7.54 (ddd, *J* = 8.6, 2.2, 1.1 Hz, 1H), 8.13 (s, 1H), 8.80 (d, *J* = 3.3 Hz, 1H), 9.74 (d, *J* = 3.3 Hz, 1H). ^13^C-NMR (100 MHz, CDCl_3_) δ (ppm): 21.16, 28.04, 33.21, 53.97, 55.35, 69.96, 115.23, 125.45, 128.35, 136.13, 157.57, 160.26, 171.87. LC-MS (ESI): m/z calcd for C_15_H_20_N_4_O_3_ [M+H]^+^: 305.160; found 305.

#### 3.3.13. N-Hydroxy-6-(4-(4-hydroxyphenyl)-2H-1,2,3-triazol-2-yl)hexanamide **VI(j)**

Black sticky solid. Yield 42.63%, R_f_: 0.48 (ethyl acetate–hexane: 3:2). IR (cm^−1^): 3378.74 (O-H), 1650.08 (C=O, amide), 1594.67 and 1474.06 (C=C, aromatic ring), 1448.54 (CH_2_ bend). ^1^H NMR (600 MHz, CDCl_3_) δ (ppm): 1.35–1.29 (m,2H), 1.57 (qdd, *J* = 12.8, 9.1, 6.0 Hz, 1H), 1.82–1.77 (m, 1H), 2.00–1.88(m,2H), 2.24 (dd, *J* = 16.1, 8.1 Hz, 1H), 2.49 (dd, *J* = 16.1, 8.1 Hz, 1H), 4.12–4.05 (m, 2H), 6.91–6.88 (m, 2H), 7.42–7.40 (m, 2H), 7.69 (s, 1H), 8.39 (d, *J* = 3.1 Hz, 1H), 9.89 (d, *J* = 3.1 Hz, 1H). ^13^C-NMR (100 MHz, CDCl_3_) δ (ppm): 24.17, 25.53, 27.38, 32.28, 54.03, 117.15, 117.06, 124.11, 128.50, 128.59, 130.69, 148.85, 159.90, 175.89. LC-MS (ESI): *m/z* calcd for C_14_H_18_N_4_O_3_ [M+H]^+^: 291.145; found 291.

#### 3.3.14. N-Hydroxy-6-(4-(*p*-tolyl)-2H-1,2,3-triazol-2-Yl)hexanamide **VI(k)**

Dark yellowish-orange sticky solid. Yield 50.29%, R_f_: 0. 73 (ethyl acetate–hexane: 3:2). IR (cm^−1^): 3158.57 (O-H), 3043.18 (C-H), 1667.46 (C=O, amide), 1648.19 and 1516.87 (C=C, aromatic ring), 1453.27 (CH_2_ bend), 1385.61 (CH_3_ bend). ^1^H NMR (600 MHz, CDCl_3_) δ (ppm): 1.51 (qdd, *J* = 5.9, 4.6, 1.1 Hz, 2H), 1.80–1.61 (m, 2H), 2.25 (t *J* = 6.7, 4.1 Hz, 2H), 3.45 (tt, *J* = 7.3, 5.1 Hz, 2H), 4.25 (dd, *J* = 14.5, 3.5 Hz, 1H), 7.26–7.25 (m, 2H), 7.80–7.78 (m, 2H), 8.10 (s, 1H), 8.81 (d, *J* = 3.3 Hz, 1H), 9.92 (d, *J* = 3.5 Hz, 1H). ^13^C-NMR (100 MHz, CDCl_3_) δ (ppm): 21.26, 24.19, 25.55, 27.39, 32.25, 54.09, 126.13, 126.22, 129.90, 129.95, 130.71, 131.18, 137.93, 148.82, 175.80. LC-MS (ESI): m/z calcd for C_15_H_20_N_4_O_2_ [M+H]^+^: 289.165; found 289.

#### 3.3.15. 3-(4-(4-(4-Chlorophenyl)-2H-1,2,3-triazol-2-Yl)phenyl)-N-hydroxypropanamide **VI(l)**

Yellowish-brown solid. Yield 56.32%, m.p: 208–211 °C, R_f_: 0.47 (ethyl acetate–benzene: 3:2). IR (cm^−1^): 3182.09 (O-H)’,1652.07 (C=O, amide), 1592.92 and 1473.08 (C=C, aromatic ring), 1487.68 (CH2 bend), 602.16 (C-Cl). ^1^H NMR (600 MHz, CDCl_3_) δ (ppm): 2.57 (dd, *J* = 17.7, 6.5 Hz, 1H), 2.82 (dd, *J* = 17.8, 6.6 Hz, 1H), 7.34–7.32 (m, 2H), 7.48–7.45 (m, 2H), 7.60–7.57 (m, 2H), 7.70–7.67 (m, 2H), 9.08 (s, 1H), 9.20 (d, *J* = 3.3 Hz, 1H). ^13^C-NMR (100 MHz, CDCl_3_) δ (ppm): 42.15, 53.50, 77.46, 122.73, 125.78, 128.19, 128.34, 130.26, 134.73, 136.81, 137.26, 143.49, 165.99, 169.92. LC-MS (ESI): m/z calcd for C_17_H_15_ClN_4_O_2_ [M+H]^+^: 343.095; found 343.

#### 3.3.16. N-Hydroxy-3-(4-(4-(*p*-tolyl)-2H-1,2,3-triazol-2-yl)phenyl)propanamide **VI(m)**

Dark muddy yellow powder. Yield 63.31%, m.p: 192–194 °C, R_f_: 0.63 (CH_3_OH–CHCl_3_: 4:1). IR (cm^−1^): 3260.28 (O-H), 2946.95 (C-H, methyl), 1687.08 (C=O, amide), 1610.39 and 1470.73 (C=C, aromatic ring), 1446.56 (CH_2_ bend), 1395.58 (CH_3_ bend). ^1^H NMR (600 MHz, CDCl_3_) δ (ppm): 2.37 (d, *J* = 13.4 Hz, 4H), 2.82 (dt, *J* = 14.7, 6.7, 0.9 Hz, 1H), 3.07 (ddt, *J* = 14.7, 6.6, 1.0 Hz, 1H), 7.39–7.37 (m, 2H), 7.53–7.51 (m, 2H), 7.76–7.73 (m, 2H), 9.57 (d, *J* = 3.3 Hz, 1H). ^13^C-NMR (100 MHz, CDCl_3_) δ (ppm): 21.37, 40.04, 70.34, 1007.45, 125.19, 125.21, 128.17, 128.19, 129.04, 129.88, 130.91, 135.34, 137.59, 140.81, 149.68, 163.86, 171.06. LC-MS (ESI): m/z calcd for C_18_H_18_N_4_O_2_ [M+H]^+^: 323.150; found 323.

#### 3.3.17. N-Hydroxy-3-(4-(4-(4-hydroxyphenyl)-2H-1,2,3-triazol-2-yl)phenyl)propanamide **VI(n)**

Brownish-white powder. Yield 50.82%, m.p: 201–203 °C, R_f_: 0.58 (ethyl acetate–benzene: 3:2). IR (cm^−1^): 3369.28 (O-H), 1647.97 (C=O, amide), 1595.41 and 1474.10 (C=C, aromatic ring), 1464.72 (CH2 bend). ^1^H NMR (600 MHz, CDCl_3_) δ (ppm): 1.15 (t, *J* = 6.9, 1.5 Hz, 3H), 3.03 (dq, *J* = 9.0, 6.8 Hz, 1H), 3.99 (s, 2H), 6.26 (dd, *J* = 1.9, 0.9 Hz, 1H), 7.04–7.02 (m, 2H), 7.34–7.32 (m, 2H), 7.54–7.51 (m, 2H), 9.17 (d, *J* = 3.1 Hz, 1H), 9.38 (d, *J* = 3.1 Hz, 1H). ^13^C-NMR (100 MHz, CDCl_3_) δ (ppm): 13.32, 44.43, 55.35, 75.14, 107.44, 114.27, 114.29, 125.01, 125.02, 125.61, 125.71, 129.75, 129.77, 139.77, 140.38, 150.77, 159.75, 163.68, 173.60. LC-MS (ESI): m/z calcd for C_17_H_16_N_4_O_3_ [M+H]^+^: 325.129; found 325.

#### 3.3.18. N-Hydroxy-3-(4-(4-(4-methoxyphenyl)-2H-1,2,3-triazol-2-Yl)phenyl)propanamide **VI(o)**

Dark yellowish-brown powder. Yield 49.62%, m.p. 206–208, R_f_: 0.51 (ethyl acetate–benzene: 3:2). IR (cm^−1^): 3145.34 (O-H), 1669.12 (C=O, amide), 1590.14 and 1487.27 (C=C, aromatic ring), 1459.49 (CH_2_ bend), 1180.80 (C-O, ether). ^1^H NMR (600 MHz, CDCl_3_) δ (ppm): 2.60–2.57 (m, 1H), 2.83 (ddt, *J* = 8.3, 7.7, 0.7 Hz, 1H), 5.72 (s, 1H), 6.12 (d, *J* = 1.0 Hz, 1H), 6.94–6.91 (m, 1H), 7.20 (m, 3H), 7.55 (d, *J* = 1.6 Hz, 1H), 8.93 (d, *J* = 3.5 Hz, 1H). ^13^C-NMR (100 MHz, CDCl_3_) δ (ppm): 31.19, 33.11, 76.71, 114.73, 123.50, 126.74, 128.47, 131.73, 136.94, 138.96, 145.83, 158.03, 159.56, 171.07. LC-MS (ESI): m/z calcd for C_14_H_17_ClN_4_O_2_ [M+H]^+^: 339.145; found 339.

### 3.4. Biological Evaluation

#### 3.4.1. Maintenance of Mammalian Cell Lines

Following an extensive review of the literature, we selected human breast cancer (MCF-7, MDA-MB-231) and lung cancer (A549) cell lines to evaluate the anticancer potential of the synthesized compounds, and some compounds with good anti-proliferation activity were again tested to evaluate their cytotoxic potential on A-549, MCF-7, and MDA-MB-231. The same were also tested on one non-cancerous/normal (HEK-293) cell line [63,64,65]. All the cell lines for the required screening were developed in a suitable medium comprising 10% FBS and 2mM L-glutamine. Cancerous and non-cancerous/normal cell lines were selected for the cell proliferation and cytotoxicity assay and maintained with 5% CO_2_ at 37 °C in a CO_2_ incubator with 100% relative humidity for one complete day before the addition of experimental drugs [66].

#### 3.4.2. Antiproliferative Assay

Antiproliferative activity through an SRB assay was performed for all the synthesized compounds on A-549 and MCF-7 cell lines. Around 5000 cells/well were quarantined into a 96-well microtiter plate in 100 μL. The synthesized compounds were solubilized in DMSO and diluted to different concentrations before use. The final-drug-concentration aliquots were poured into microtiter wells, and the plates were incubated for 48 h in regular conditions. After adding the final drug concentration, the cells were allowed to set in place and incubated for 60 min. A total of 50 µL of SRB solution (0.4% *w*/*v*) in 1% CH_3_COOH was poured into each well. The plates were then incubated for 20 min at RT. During subsequent staining, the unbounded dye was removed, and the plate reader recorded the eluted bound stain at 540 nm with a 690 nm reference wavelength. The assay was performed precisely using the appropriate reagents and reaction situation, as per the reported procedure [38,39]. ADR was selected as the reference drug.

#### 3.4.3. Cytotoxicity Assay

MTT, a well-known colorimetric cytotoxic assay was performed to validate the cytotoxicity [40] of the selected compounds—**VI(b)**, **VI(c)**, **VI(e)**, and **VI(i)**—against three different human tumor cell lines [MCF-7, MDA-MB-231, A549] and a normal cell line [HEK293]. The seeding of 1 × 10^4^ cells onto each well was executed in a 96-well plate in triplicate, and to keep the cells viable, overnight incubation was undertaken at 37 °C with 5% CO_2_ in a CO_2_ incubator. Following overnight incubation, the exhausted complete medium was removed and exchanged with fresh complete medium comprising various concentrations of the compounds [**VI(b)**, **VI(c)**, **VI(e)**, and **VI(i)**]; then, the 96-well culture plate was kept for 24 h of incubation at 37 °C. Compounds incorporating the medium were discarded and replaced by fresh MTT comprising the complete medium and incubated at 37 °C for 2 h. During this 2 h incubation period, purple-colored formazan crystals were produced by the active cells via enzymatic reaction cascades. A total of 100 µL DMSO/well was poured into each well to solubilize the formed purple crystals and left for half an hour at 37 °C. The microplate reader was set at 570 nm to measure the absorbance of each well.

#### 3.4.4. Apoptosis Study Using Acridine Orange/Ethidium Bromide (AO/EtBr) Dual-staining Technique

The staining technique was performed to determine changes in cellular morphology as acridine orange is a cell-permeable dye; i.e., it can enter inside the nucleus and subsequently intercalate with the DNA of both live and apoptotic cells an then emit a green fluorescence [41]. On the other hand, ethidium bromide can only cross the plasma membrane of compromised or late apoptotic cells and bind with their DNA, subsequently radiating an orange to red fluorescence [42]. Therefore, to assess alterations in the morphology of cells, MCF-7 cells were planted in a 12-well plate at a density of 5 × 104 cells/well and incubated in humidified air for 24 h at 37 °C. Following cell seeding, MCF-7 cells were exposed to compound **VI(i)** with at lower (40 µg/mL), IC_50_ (60 µg/mL), and higher (80 µg/mL) concentrations. Following this, the incubated cells were washed with a buffer (phosphate-buffered saline), and dual staining was executed with acridine orange and ethidium bromide. Then, a 12-well cell culture plate was kept at 37 °C for 30 min of incubation. Consequently, cells were lightly rinsed with phosphate-buffered saline. Following this, pictures were obtained in the inverted fluorescence microscope using the red and green channels for both the dyes.

#### 3.4.5. Detection of Enhanced Reactive Oxygen Species (ROS)

ROS are commonly produced as a result of the cellular metabolic routes, and they play a key role as a secondary messenger in the healthy physiological system, but elevated quantities of ROS are the reason for various pathological conditions [43,44]. To assess oxidative stress (ROS)-mediated apoptosis, DCFH-DA (2′,7′-dichlorodihydrofluorescein diacetate) dye was used as ROS transforms DCFH to DCF, producing a green fluorescence. The intensity of green fluorescence emitted is directly correlated with the amount of ROS produced by the cells in the treatment groups. Briefly, MCF-7 cells were passaged then seeded (5 × 104 cells /well) in a 12-well plate and exposed to all three (lower, IC_50_, higher) concentrations of compound **VI(i)**; then, overnight incubation was undertaken with 5% carbon dioxide at 37 °C. To assess the generated ROS, treated cells (MCF-7) were washed with PBS. Staining was undertaken using DCFH-DA and photographed with the help of a fluorescence microscope (inverted) in the green and phase contrast channels.

#### 3.4.6. Analysis of Cell Cycle Distribution

Apoptosis can also be determined based on the content of the DNA allocation at different stages of cell cycle distribution. The cultured MCF-7 cell line was washed, trypsinized, and seeded, followed by compound **VI(i)** exposure in a 6-well cell culture plate. Following this, cells were harvested using 1mM EDTA; then, to fix the cells, ice-cold ethanol was used, and overnight, cells were kept at −20 °C. Afterwards, cells were maintained at room temperature, followed by centrifugation at 3000 RPM for 7 min. Then, staining was conducted using propidium iodide, RNase A, and Triton-X in a buffer (phosphate-buffered saline). It was then analyzed using a flow cytometer.

#### 3.4.7. Determination of Apoptosis Through Annexin V/Propidium Iodide (PI)

Qualitative analysis of different stages of apoptosis in MCF-7 cells using AO/EtBr dual staining was further quantitatively validated using annexin V/PI staining. Annexin V binds with the exposed phosphatidyl serine of apoptotic cells in a calcium-dependent manner and permits the determination of a number of cells in different stages of apoptosis [45]. At the same time, PI binds with the nucleic acid of dead cells. In brief, cells were plated in a 6-well cell culture plate followed by exposure to lower, IC_50_, and higher concentrations of compound **VI(i)**, as described in the above experiments. Following 24 h of incubation, treated cells were harvested using 1 millimole EDTA; then, annexin V/PI staining was performed, which was incubated for 30 min at 37 °C. The population of the apoptotic cells was examined by evaluating the cell suspension using a flow cytometer.

#### 3.4.8. HDAC Inhibition Assay

Using color de lys ELISA assay kits (Mybiosource, Inc., San Diego, CA, USA), and following the manufacturer’s’ instructions, the in vitro HDAC–inhibitory assay of some selected VI series compounds **[VI(b)**, **VI(c)**, **VI(e)**, and **VI(i)]** against HDAC1 and HDAC6 isoforms was determined. SAHA served as the assay’s positive control. The HeLa nuclear extract was used as a source of HDAC activity. The trypsinized HeLa cells were tallied, put into 96-well microtiter plates, and nurtured. Samples and the standard were placed in wells at a concentration of 100 µL. The culture environment was maintained as per the procedure. 3,3′,5,5′-tetramethylbenzidine (TMB) was included for visualization. Finally, 50 µL of halt solution was smeared. The samples and different SAHA concentrations (0.01, 0.1, 1, 10 μM) were introduced to the appropriate well and left for 72 h. BioTek Epoch (Winooski, VT, USA) microplate reader was used to quantify optical density at 450 nm. The Graph Pad Prism 5 helped to determine the IC_50_ values [46,47].

### 3.5. Molecular Modeling Studies

#### 3.5.1. Molecular Docking

Molecular docking was conducted on all the synthesized compounds against HDAC1 (PDB: 1C3S) and HDAC6 (PDB: 1EEI) isoforms by the Glide module of Maestro. Supplementary Materials provided a detailed explanation of procedure [48,49,50,51,52].

#### 3.5.2. Molecular Dynamic (MD) Simulations

The top docked compound, **VI(i)**, was selected for MD simulation using the Desmond (v 2.0) module of Maestro. The HDAC–inhibitor complex was simulated for 100 ns. The prior step before any simulation is to mount an appropriate biological system through the system builder panel [53]. The compound was engrossed in a predefined SPC water model as a solvent in an orthorhombic figured box of 10 × 10 × 10 Å dimensions [54]. Salt was added in a definite concentration of Na+ (50.901 nM) and Cl- (61.081 mM), bringing it to a total charge of +30 and −36, respectively. The box volume was minimized in an optional mode. A system-built/default option was employed to ensure that the created system was electrically neutral before proceeding for simulation. The counter ions were included to neutralize the simulated protein system in an aqueous solution. Commonly, the charges-stabilizing counter ions were added in close proximity to charged scums to nullify the net charge on the protein surface. The system then underwent energy minimization through inadequate memory Broyden–Fletcher–Goldfarb–Shanno algorithms, accompanied by 10 steepest descent steps and 03 vectors, until a gradient threshold of 25 kcal/Mol/Å was established [55]. Generally, isothermal–isochoric NVT and isothermal–isobaric NPT assemblages are used to equilibrate the system. The dynamic simulation was proficient under the NPT ensemble for 100 ns at 300 K and 1 bar pressure [56,57]. The thermostat and barostat were implemented via the Nose–Hoover chain and Martyna–Tobias–Klein techniques, respectively. The SHAKE algorithm was smeared to oblige the hydrogen bonds. Electrostatic interactions in protein–ligand complexes were analyzed using the PME technique with harmonic constraints scaling [58].

#### 3.5.3. MD Trajectory Analysis

The analyzed trajectory frame measured the essential structural features of this molecular dynamics trajectory and determined various geometric properties for every simulation frame. Every 100 p defined the trajectory of the recording interval. A total of 2000 frames were captured throughout the simulation [59]. The RMSD values and distance to the Zn ion were calculated engaging the Simulation Event Analysis panel. The backbone atoms of the protein were used to calculate the RMSD, whereas the RMSD of the ligand and Zn ion were computed via fitting to the protein mainstay. The RMSF and receptor–ligand contacts were examined through the Simulation Interaction Diagram panel.

#### 3.5.4. Free Binding Energy Calculation (MM-GBSA)

The MM/GBSA technique enables the determination of the binding free energy (∆G_MM/GBSA_). The prime 3.1 module was applied to the best few hits to ascertain the free binding energy.

#### 3.5.5. ADME and Toxicity Predictability Studies

The QikProp v 5.4 was used to forecast the ADME assets of the synthesized compounds, and “ProTox 3.0” toxicity predictor software aided in providing data for molecules with favorable toxic endpoints and predicting the expected loading dose of the synthesized compounds.

#### 3.5.6. Density Functional Theory (DFT) Investigations

In our research, we considered correlating the structure and function of the two best-docked compounds, **VI(i)** and **VI (j)**, along with an established marketed drug, SAHA. Analyzing this correlation through DFT was the preferred choice. The GAUSSIAN 16 software package was employed for all theoretical calculations, followed by result visualization on GAUSS-VIEW 6.1 [60]. This software utilized Becke’s three-parameter method for exchange interaction and Lee–Yang–Parr for correlation functional (B3LYP), along with the 6-31+G(d,p) basis sets for calculating the total energy of orbitals, dipole moments, HOMO, LUMO energy levels, and band gap energy [60].

## 4. Conclusions

The present research dealt with the plan and synthesis of a series of fifteen novel 1,2,3-triazole-based hydroxamides as HDAC inhibitor. The compounds were evaluated for their biological activities. The computational approaches of molecular docking, MD simulation, MM/GBSA binding free energy computation, and ADMET prediction were also explored to study the underlying molecular mechanism behind enzymatic inhibition. The biological and computational data revealed that the 6-carbon straight methylene chain as a linker and the *p*-methoxy phenyl clubbed 1,2,3-triazole ring as a cap group had a significant effect on anticancer activity. The cytotoxic study was performed against MCF-7, MDA-MB-231, and A-549 cell lines, which showed compounds **VI(b)**, **VI(c)**, **VI(e)**, and **VI(i)** as the most active among all. In the MTT experiment, compound **VI(i)** exhibited modest cytotoxicity against MCF-7, though its antiproliferative activity was more prominent in the SRB assay. However, over time, a decline in activity of **VI(i)** was observed by the researchers, and this could be one of the main reasons behind the limited activity of **VI(i)** in the MTT assay. There was a time gap of a few months between the assays; the MTT assay was performed a few months after performing SRB assay, as a sort of validation to reconfirm the cell line for further biological assays. Despite comprehensive evaluation across multiple cancer cell lines, compound **VI(i)** revealed remarkable selectivity as an anti-breast cancer drug candidate. It exhibited potency against MCF-7 cells (IC_50_ = 60 μg/mL) while showing minimal activity against other cancer lines (IC_50_ >100 μg/mL), with low toxicity toward normal HEK-293 cells. The compound’s ability to induce ROS-mediated apoptosis, S and G2/M phase cell cycle arrest, superior HDAC1/HDAC6 inhibition tendency (IC_50_ values = 3.06 μg/mL for HDAC1 and 4.08 μg/mL for HDAC6), favorable pharmacokinetic properties, and stable in silico interaction with HDAC1 and HDAC6 proteins establishes it as a promising compound with significant therapeutic potential. It also displayed a higher dock score value and binding efficacy than the reference drug, SAHA. The binding interaction of **VI(i)** with HDAC proteins was further confirmed via MD simulation. The MM-GBSA experiment also established the notable binding stability of the 1C3S-**VI(i)** complex over the 5EEI-**VI(i)** complex. DFT calculations showed greater reactivity and the possibility of stronger interactions with **VI(i)** through greater dipole moment and a small HOMO-LUMO energy gap. The computational output collectively validated the higher biological activity of **VI(i)** at the molecular level. Thus, a clear link has been established between the computational predictions and their biological relevance, suggesting 1,2,3-triazole-based hydroxamide analogs as leading compounds in the development of future anticancer medicines.

## Figures and Tables

**Figure 7:**
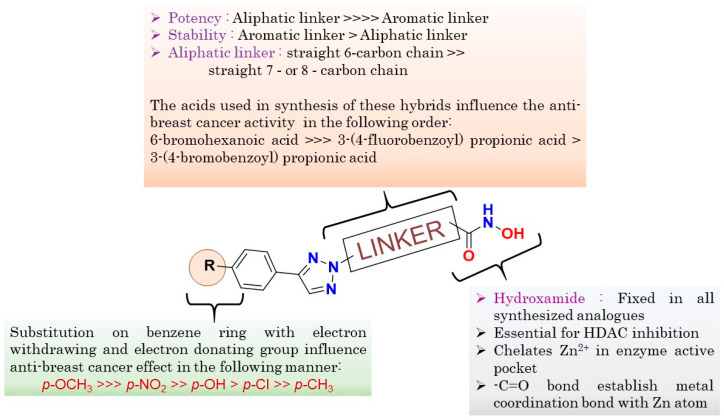
General SAR of the synthesized compounds.

**Figure 10:**
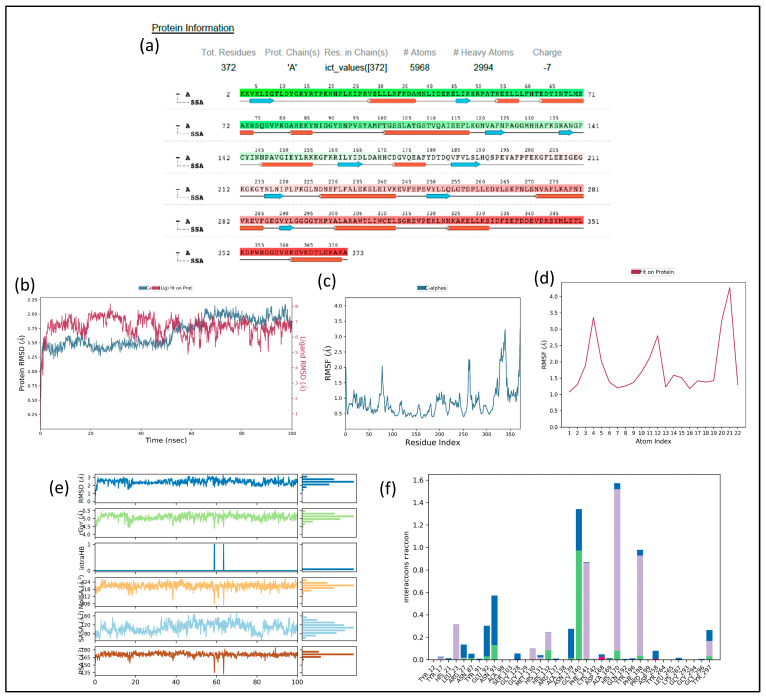
(**a**) Information about HDAC1 (1C3S) protein during 100 ns simulation. (**b**) Observed RMSD values of **VI(i)** in the active site of 1C3S protein. (**c**) Observed RMSF of **VI(i)**. (**d**) Observed RMSF of 1C3S. (**e**) **VI(i)** properties. (**f**) Observed protein–ligand contact interaction histogram.

**Figure 11:**
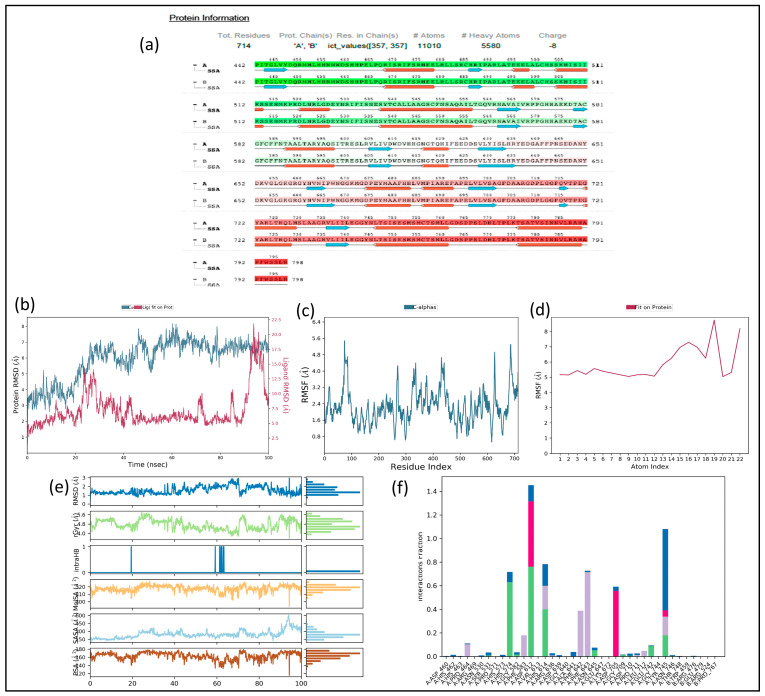
(**a**) Information about HDAC6 (5EEI) protein during 100 ns simulation. (**b**) Observed RMSD values of **VI(i)** in the active site of 5EEI protein. (**c**) Observed RMSF of **VI(i)**. (**d**) Observed RMSF of 5EEI. (**e**) **VI(i)** properties. (**f**) Observed protein–ligand contact interaction histogram.

**Table 1:** In vitro antiproliferative activities of synthesized compounds **VI(a–o)** using an SRB assay.

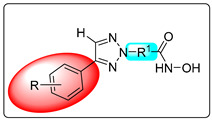				
Compd	R	R^1^ (Linker)	GI_50_ (μg/mL) ^a^	
			A-549 (Human Lung Cancer Cell Line)	MCF-7 (Human Breast Cancer Cell Line)
**VI(a)**	p-OCH_3_ phenyl	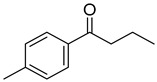	19.35	15.13
**VI(b)**	p-NO_2_ phenyl		<10	<10
**VI(c)**	p-Cl phenyl		<10	<10
**VI(d)**	p-CH_3_ phenyl		14.69	12.91
**VI(e)**	p-OH phenyl		<10	<10
**VI(f)**	p-F phenyl		26.92	<10
**VI(g)**	3,4-diOCH_3_ phenyl		53.00	15.00
**VI(h)**	p-Cl phenyl	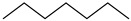	>80	50.00
**VI(i)**	p-OCH_3_ phenyl		<10	<10
**VI(j)**	p-OH phenyl		50.00	17.00
**VI(k)**	p-CH_3_ phenyl		>80	14.86
**VI(l)**	p-Cl phenyl	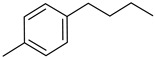	>80	>80
**VI(m)**	p-CH_3_ phenyl		>80	>80
**VI(n)**	p-OH phenyl		>80	>80
**VI(o)**	p-OCH_3_ phenyl		62.00	>80
**ADR**	-	-	<10	<10

^a^ GI_50_ = compound concentration required to inhibit 50% growth, and data are expressed as the mean from the dose–response curves of at least three experiments; S.D. < 10%.

**Table 2:** In vitro HDAC–inhibitory activity of selected compounds.

Comp. No.	IC_50_ Values ^a^ (μg/mL)	
	HDAC1	HDAC6
**VI(b)**	8.04	9.21
**VI(c)**	7.87	11.76
**VI(e)**	9.60	6.57
**VI(i)**	3.06	4.08
**SAHA ^b^**	0.08	0.09

^a^ Experiments were carried out in triplicate; i.e., IC_50_ values are mean of three independent experiments; S. D. < 10%. ^b^ SAHA was employed as positive reference.

**Table 3:** The extra precision (XP) dock score value of synthesized substituted-1,2,3-triazole-based-hydroxamide analogs [**VI(a–o)**], along with their interaction with different amino acids of HDAC1 (PDB ID:1C3S) and HDAC6 (PDB ID: 5EEI) proteins.

Sr. No.	Compd. Code	Dock Score (kcal/mol)	
		HDAC1: 1C3S	HDAC6: 5EEI
**1**	**VI(a)**	−9.002	−8.449
2	**VI(b)**	−8.301	−7.096
3	**VI(c)**	−9.446	−7.065
4	**VI(d)**	−8.562	−7.646
5	**VI(e)**	−8.343	−6.718
6	**VI(f)**	−9.308	−7.937
7	**VI(g)**	−8.398	−7.841
8	**VI(h)**	−9.883	−9.718
9	**VI(i)**	−10.691	−11.373
10	**VI(j)**	−10.482	−10.358
11	**VI(k)**	−9.951	−9.581
12	**VI(l)**	−9.770	−9.509
13	**VI(m)**	−10.047	−10.201
14	**VI(n)**	−8.629	−10.073
15	**VI(o)**	−9.395	−10.349
16	**SAHA**	−9.724	−11.261

**Table 4:** Binding free energies of HDAC protein–**VI(i)** ligand complexes via MMGBSA.

MM/GBSA Energy Descriptors (kcal mol^−1^)	1C3S-VI(i)		5EEI-VI(i)	
	0 ns	100 ns	0 ns	100 ns
MMGBSA_dG_Bind	−76.1329	−91.8075	−55.2446	−59.4738
MMGBSA_dG_Bind_Coulomb	−36.8718	−37.8267	4.5912	38.9528
MMGBSA_dG_Bind_Hbond	−1.4332	−0.9737	−1.5613	−0.2904
MMGBSA_dG_Bind_Lipo	−22.7017	−32.4736	−25.5294	−19.6282
MMGBSA_dG_Bind_Packing	−10.228	−1.876	−0.000019	0
MMGBSA_dG_Bind_Solv_GB	28.0751	23.7755	−0.0757	−35.1282
MMGBSA_dG_Bind_vdW	−35.9366	−47.8496	−37.0841	−20.7722
Complex_Energy	−11,968.88	−11,986.90	−23,399.1	−23,378.5
Complex_Coulomb	−11,349.10	−11,349.80	−23,184.5	−23,147
Complex_Hbond	−162.939	−162.48	−351.914	−350.643
Complex_Lipo	−2045.13	−2053.54	−4366.95	−4361.11
Complex_Packing	−21.9893	−13.449	−25.4552	−25.4552
Complex_Solv_GB	−2737.34	−3745.27	−3372.43	−3409.2
Complex_vdW	−1254.67	−1263.47	−2654.44	−2629.52

**Table 5:** Different energy of VI(j), VI(i) and SAHA after the DFT analysis.

Parameters	VI(j) (Best Docked Compound Among the Series)	VI(i) (Compound with Good Anticancer Activity)	SAHA (Marketed HDAC Inhibitor)
Total energy (eV)	−26,909.72	−27,978.74	−23,962.34
HOMO energy (eV)	−6.49	−6.40	−6.39
LUMO energy (eV)	−1.62	−1.59	−1.43
Band gap (eV)	4.87	4.81	4.96
Dipole moment	5.19	6.49	2.93

## Data Availability

The original contributions presented in this study are included in the article/Supplementary Materials. Further inquiries can be directed to the corresponding authors.

## Supplementary Materials

The following supporting information can be downloaded at: https://www.mdpi.com/article/10.3390/ph18081148/s1, Figure S1: Growth curve shown by compounds for (a) Human lung (A-549), and (b) Human breast (MCF-7) cancer cell lines, during SRB assay, S.D. < 10%; Table S1: ADMET analysis of the synthesized compounds using the QikProp and Protox 3.0 tool.
