# Supplementary material for: Heteroaryl-Capped Hydroxamic Acid Derivatives with Varied Linkers: Synthesis and Anticancer Evaluation with Various Apoptosis Analyses in Breast Cancer Cells, Including Docking, Simulation, DFT, and ADMET Studies
Source: Pharmaceuticals (Basel). 2025 Aug 1;18(8):1148. doi: 10.3390/ph18081148 (PMC12389269; doi:10.3390/ph18081148)
Supplement: Supplementary file 1 [file pharmaceuticals-18-01148-s001.zip › pharmaceuticals-3766876-supplementary.pdf]

Heteroaryl-capped hydroxamic acid derivatives with varied linkers: Synthesis and Anticancer evaluation with various apoptosis analysis in breast cancer cells including docking, simulation, DFT and ADMET studies

Description of the Spectral Analysis

Spectral data of compound III(a)

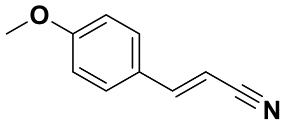

3-(4-methoxyphenyl)acrylonitrile
Chemical Formula: $C_{10}H_9NO$
Molecular Weight: 159.19

IR-spectra of compound III(a)

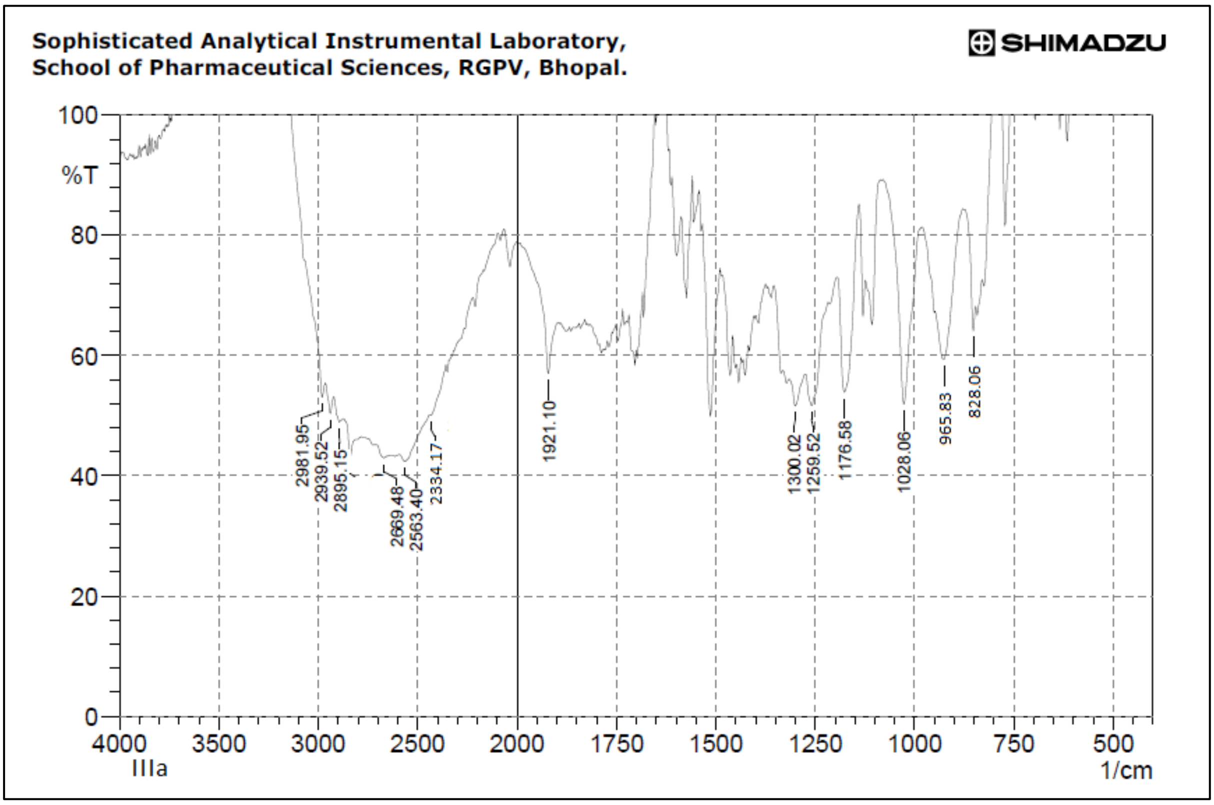

H[1]-NMR-spectra of compound III(a)

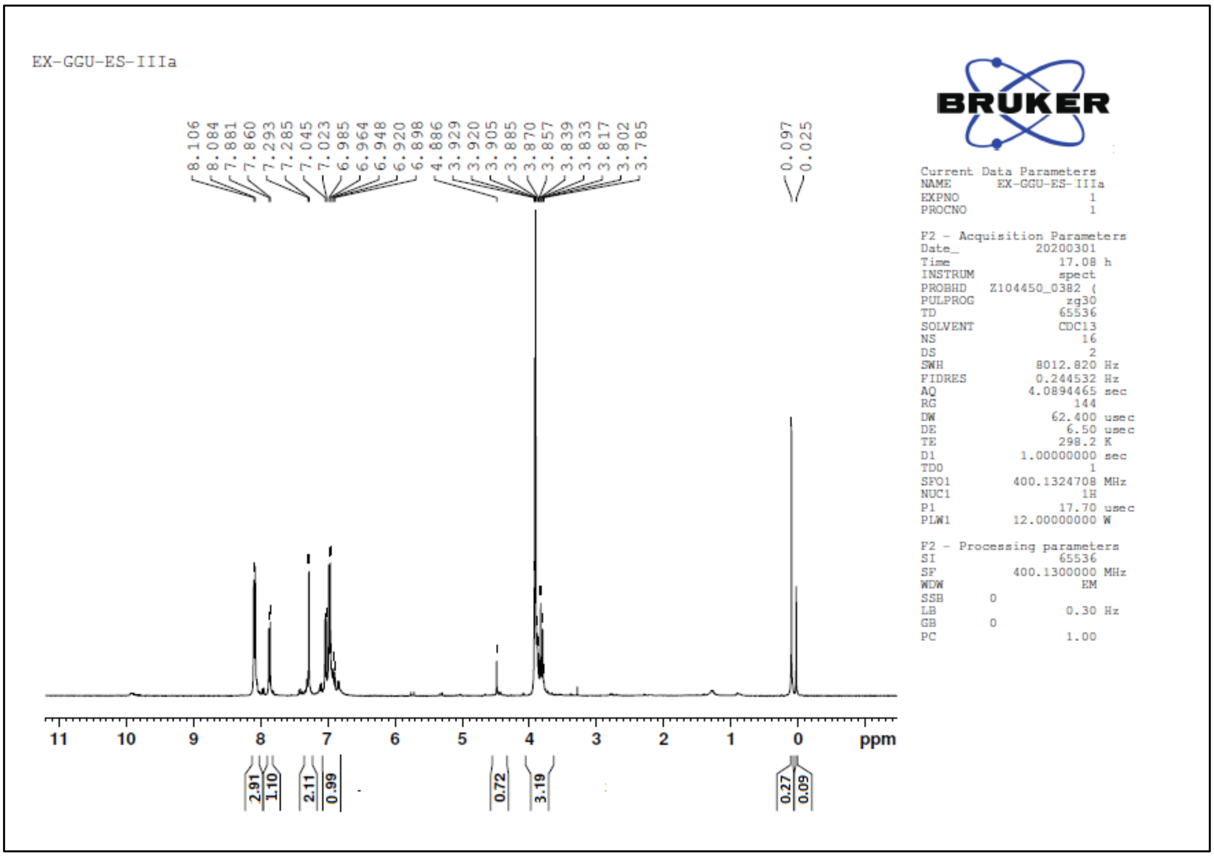

^{13C}NMR-spectra of compound III(a)

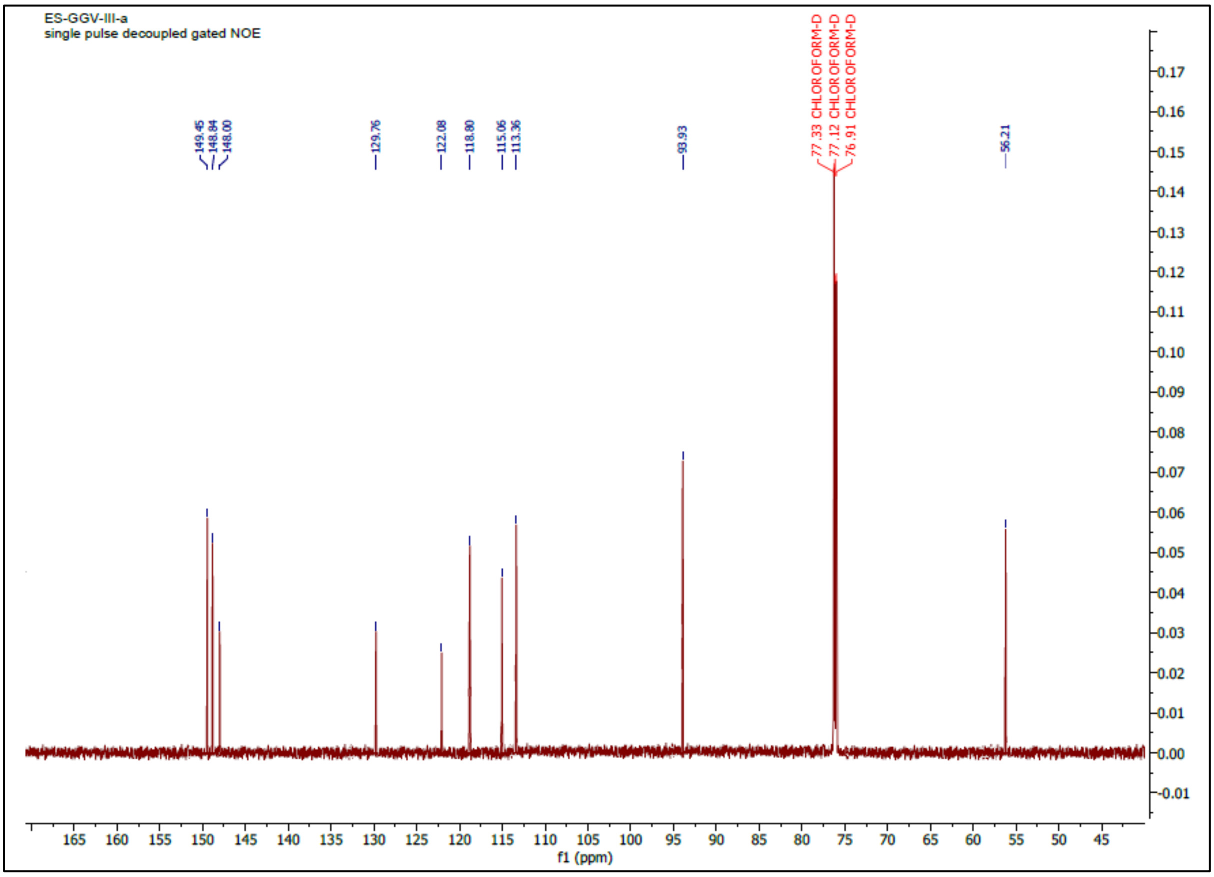

Mass-spectra of compound III(a)

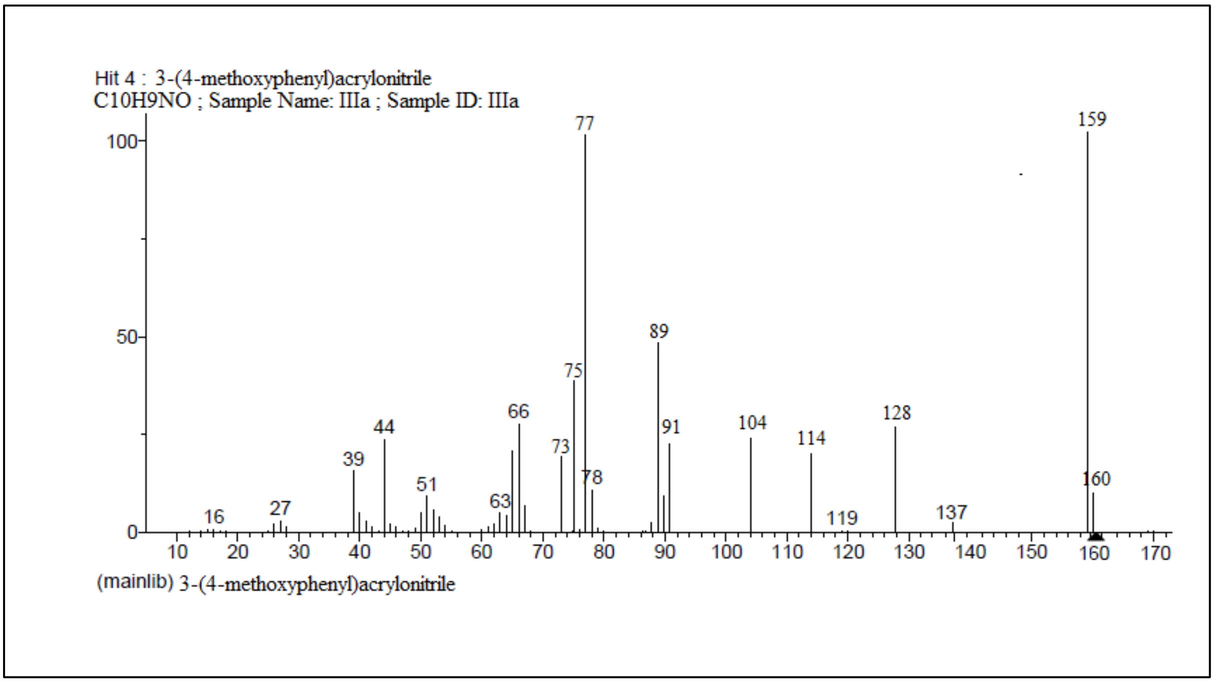

Spectral data of compound IV(a)

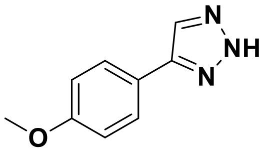

4-(4-methoxyphenyl)-2*H*-1,2,3-triazole
Chemical Formula: $C_9H_9N_3O$
Molecular Weight: 175.19

IR-spectra of compound IV(a)

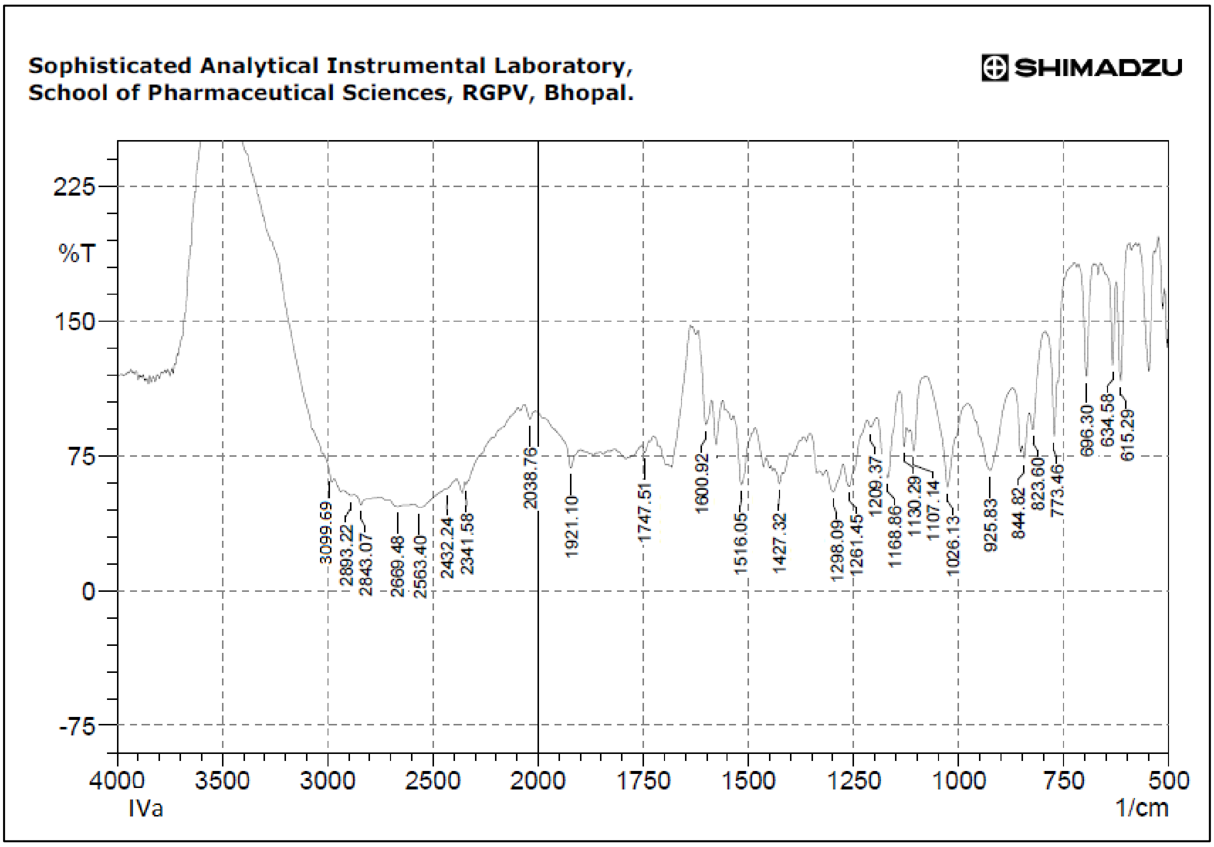

H[1]-NMR-spectra of compound IV(a)

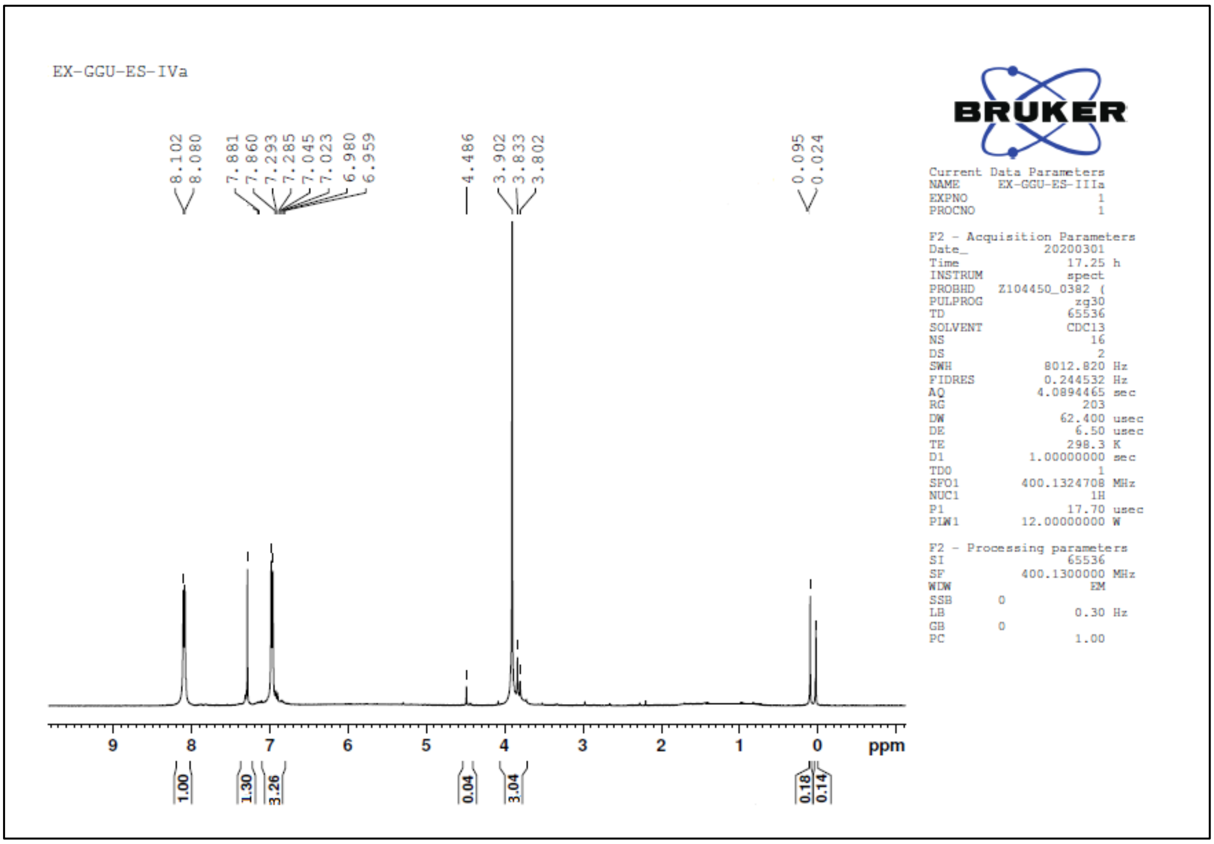

[13C]NMR-spectra of compound IV(a)

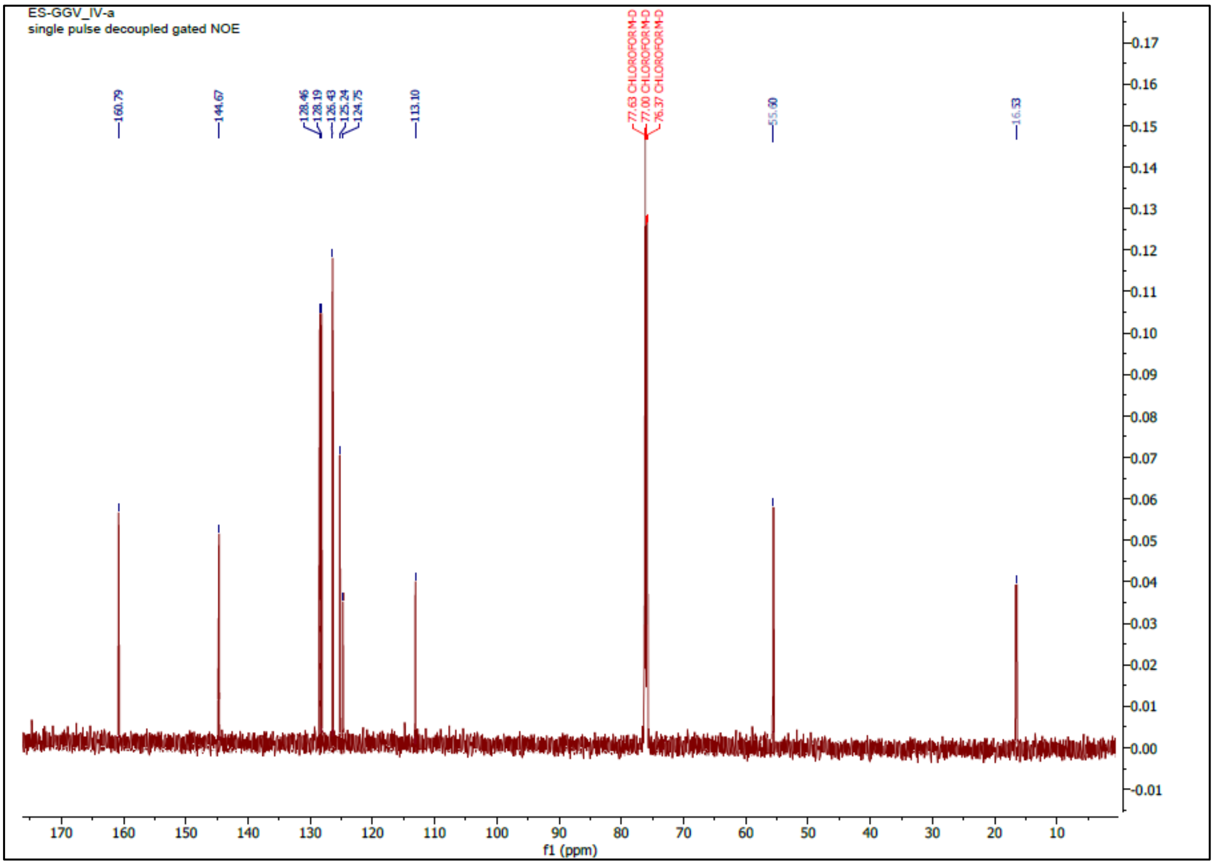

Mass-spectra of compound IV(a)

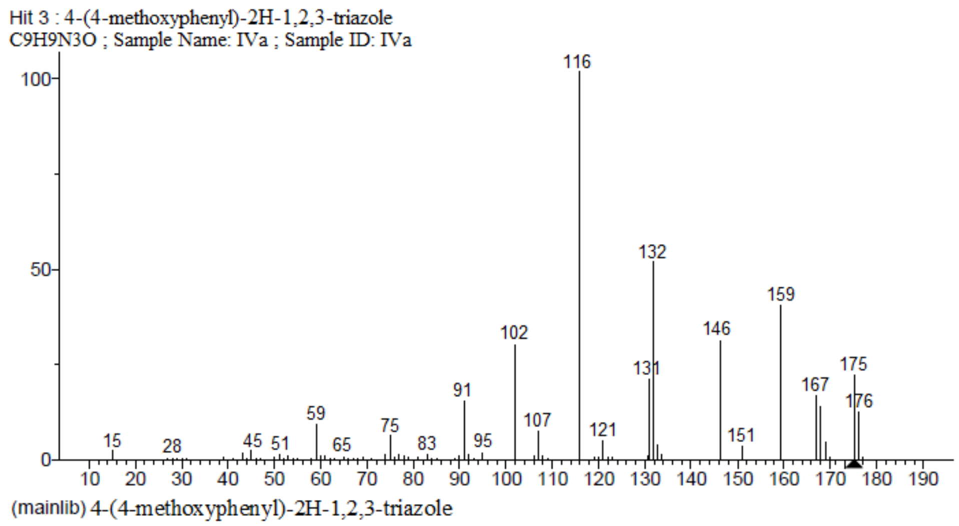

Spectral data of compound V(a)

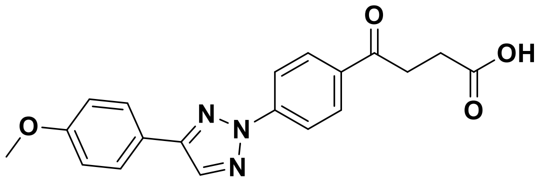

4-(4-(4-(4-methoxyphenyl)-2*H*-1,2,3-triazol-2-yl)phenyl)-4-oxobutanoic acid
Chemical Formula: $C_{19}H_{17}N_3O_4$
Molecular Weight: 351.36

IR-spectra of compound V(a)

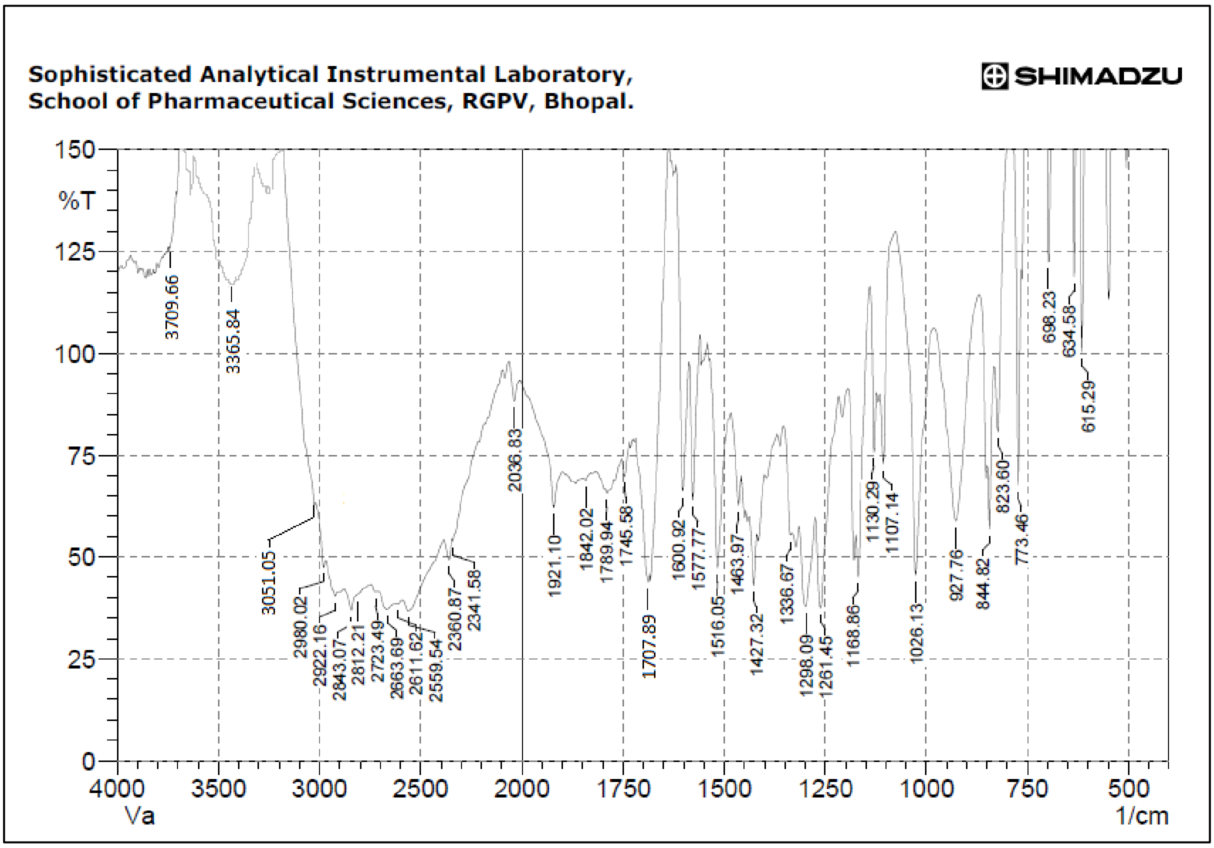

H^1-NMR-spectra of compound V(a)

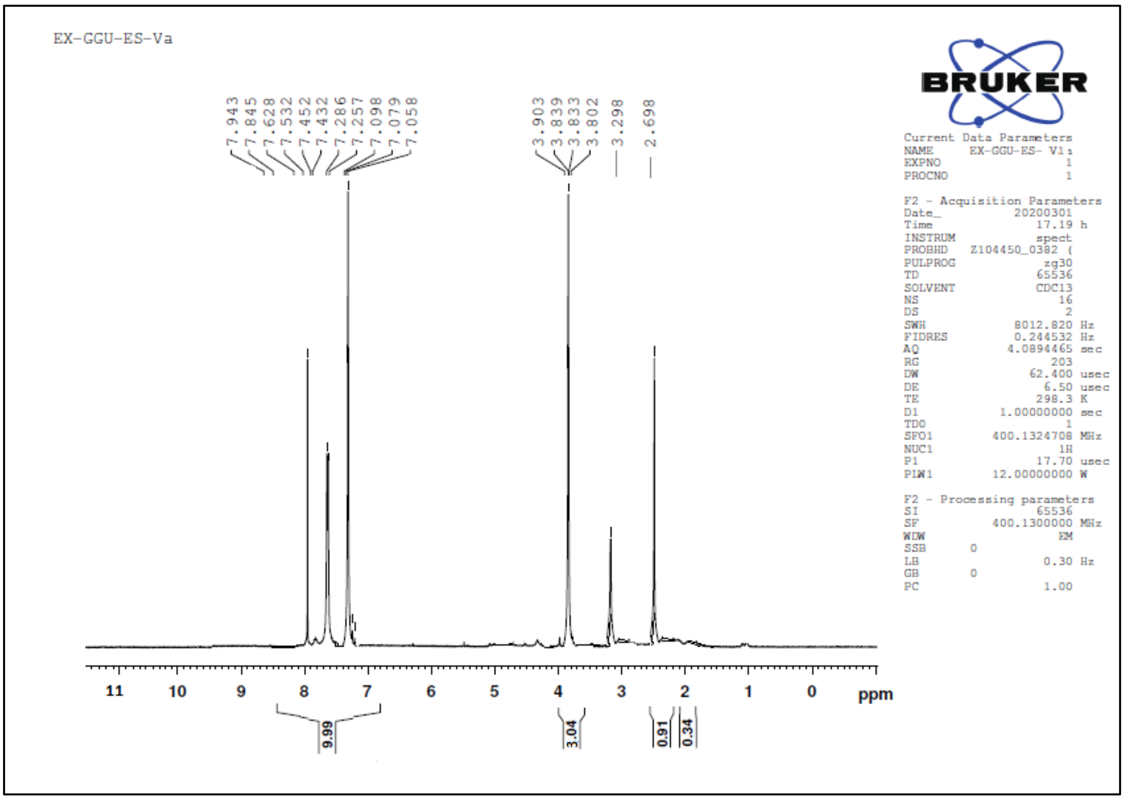

^{13C}NMR-spectra of compound V(a)

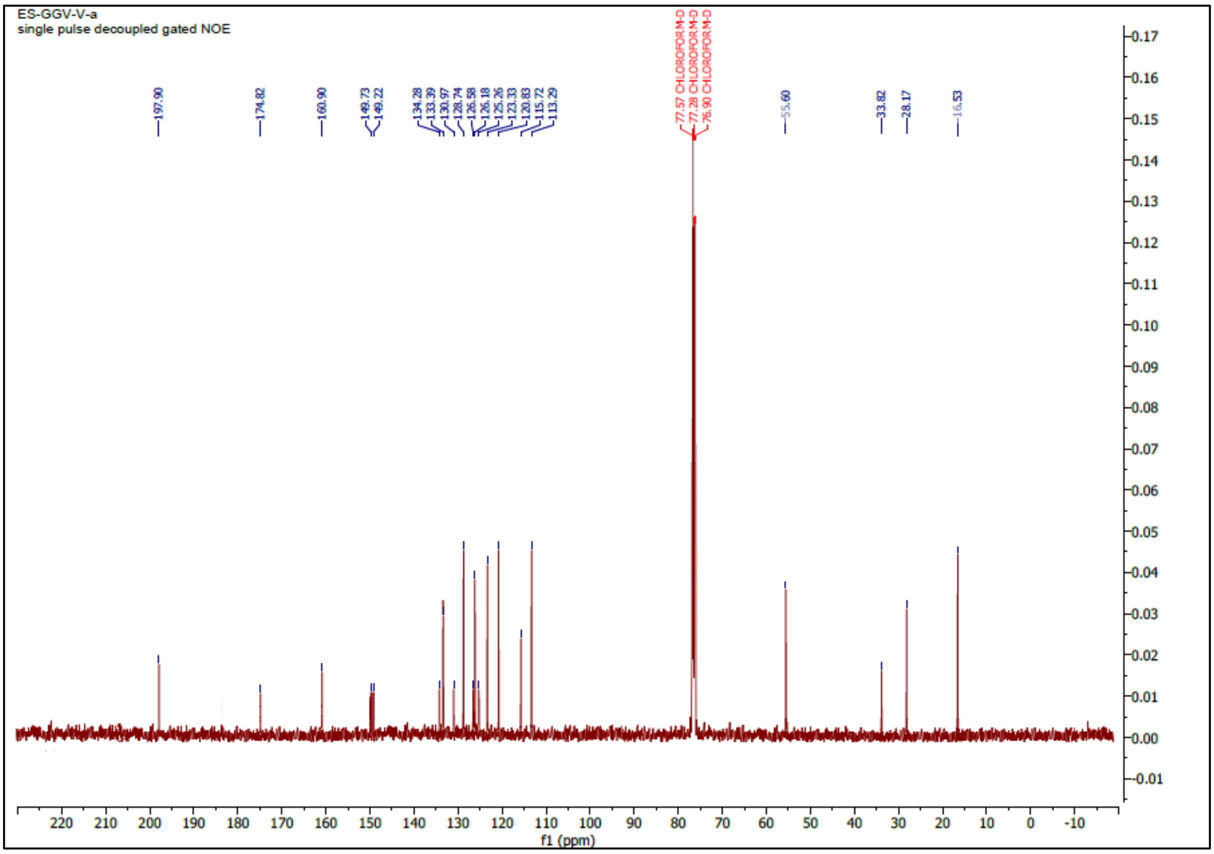

Mass-spectra of compound V(a)

Hit 5 : 4-(4-(4-(4-methoxyphenyl)-2H-1,2,3-triazol-2-yl)phenyl)-4-oxobutanoic acid
C19H17N3O4 ; Sample Name: Va ; Sample ID: Va

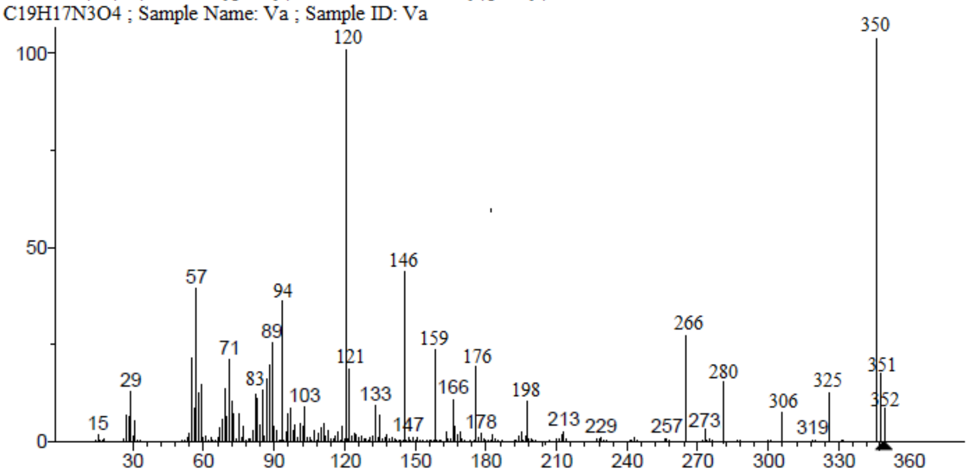

(mainlib) 4-(4-(4-(4-methoxyphenyl)-2H-1,2,3-triazol-2-yl)phenyl)-4-oxobutanoic acid

Spectral data of compound VI(a)

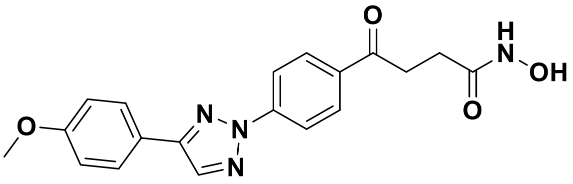

N-hydroxy-4-(4-(4-(4-methoxyphenyl)-2*H*-1,2,3-triazol-2-yl)phenyl)-4-oxobutanamide
Chemical Formula: $C_{19}H_{18}N_4O_4$
Molecular Weight: 366.38

IR-spectra of compound VI(a)

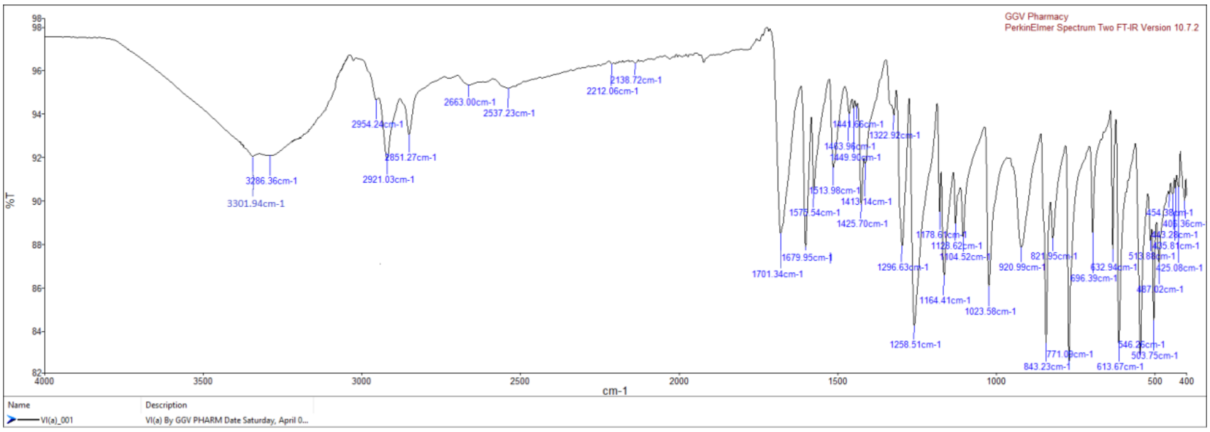

H[1]-NMR-spectra of compound VI(a)

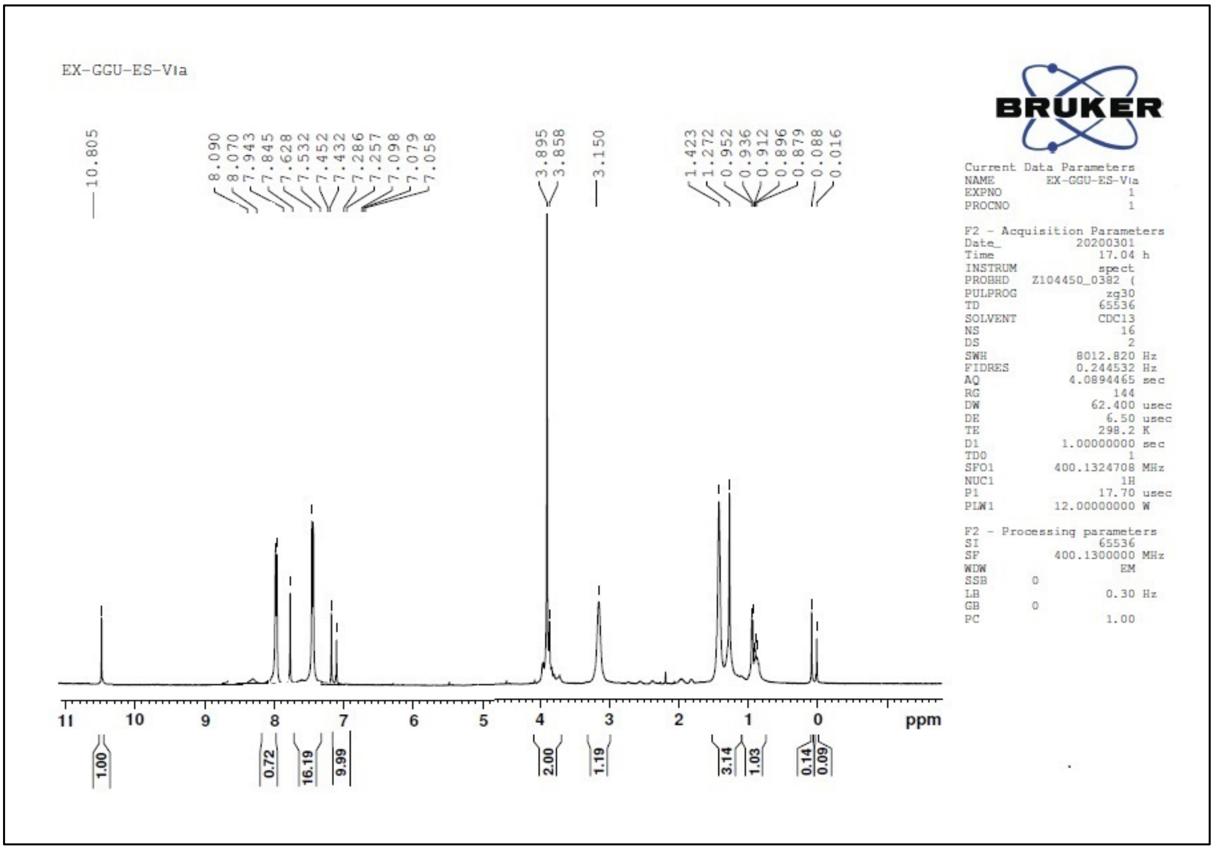

^{13C}NMR-spectra of compound VI(a)

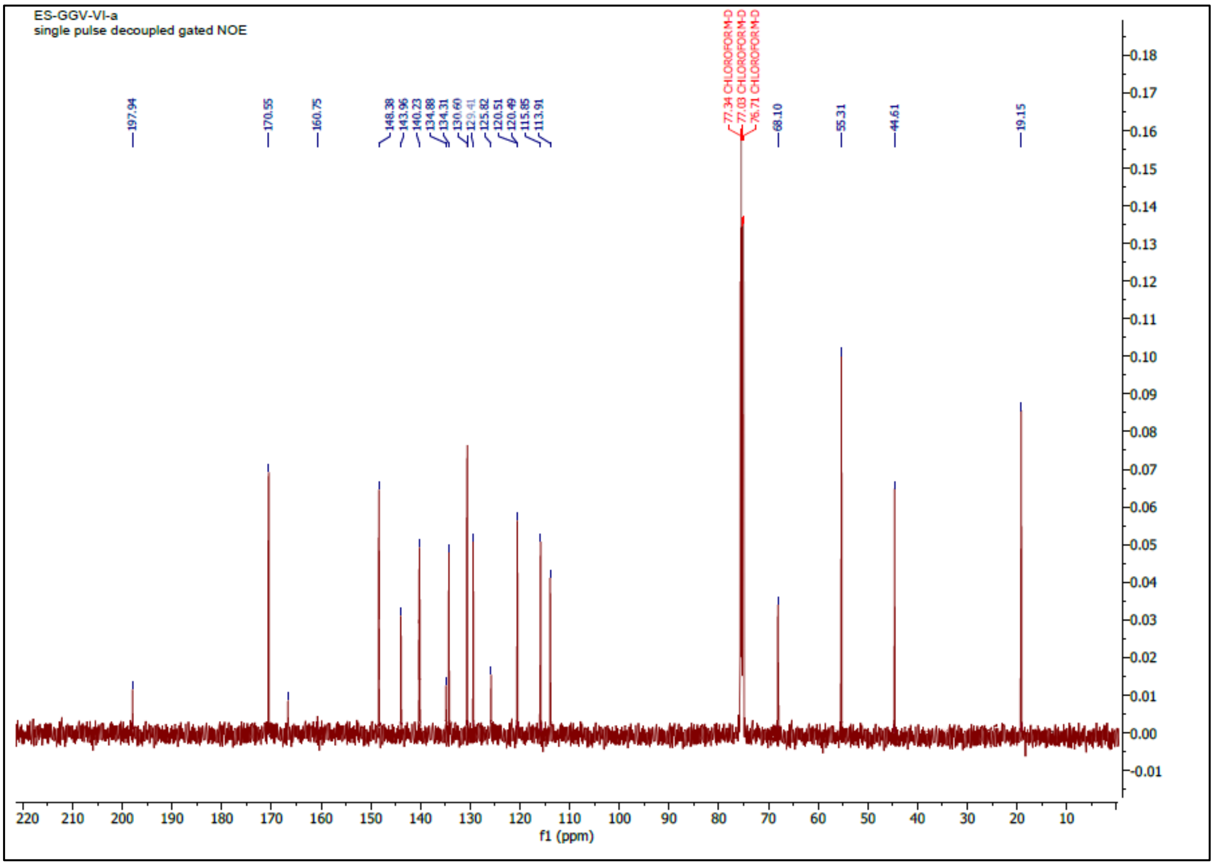

Mass-spectra of compound VI(a)

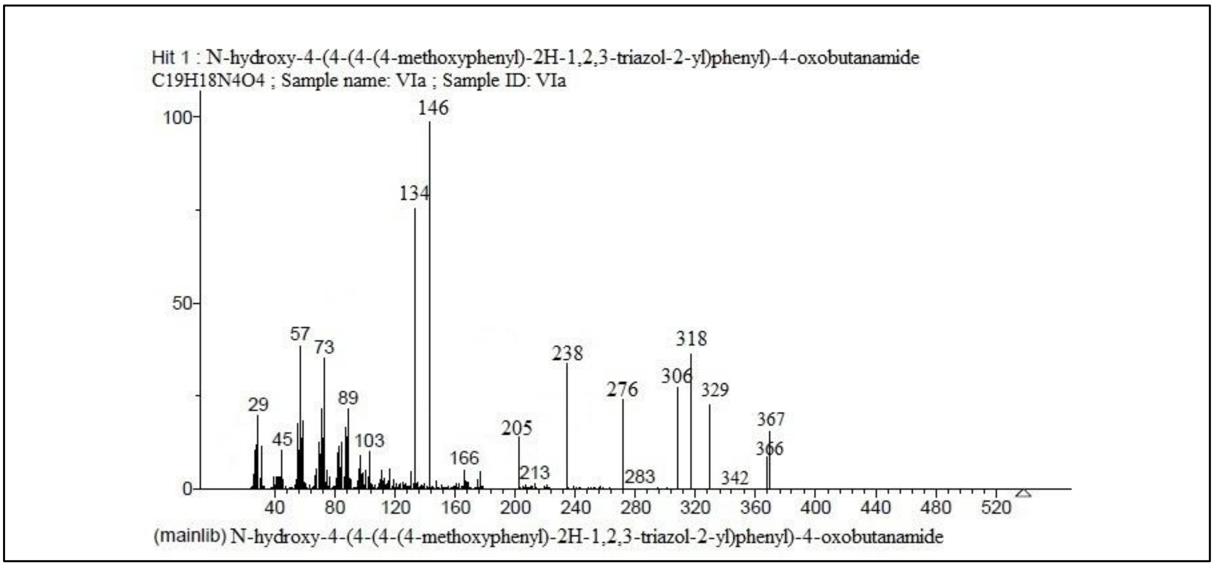

Spectral data ofcompound VI(b)

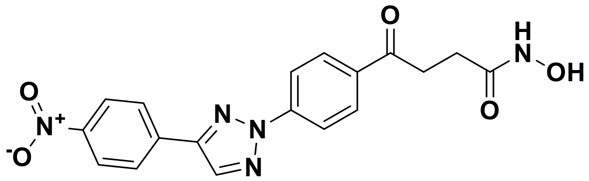

N-hydroxy-4-(4-(4-(4-nitrophenyl)-2*H*-1,2,3-triazol-2-yl)phenyl)-4-oxobutanamide

Chemical Formula: $C_{18}H_{15}N_5O_5$

Molecular Weight: 381.35

IR-spectra of compound VI(b)

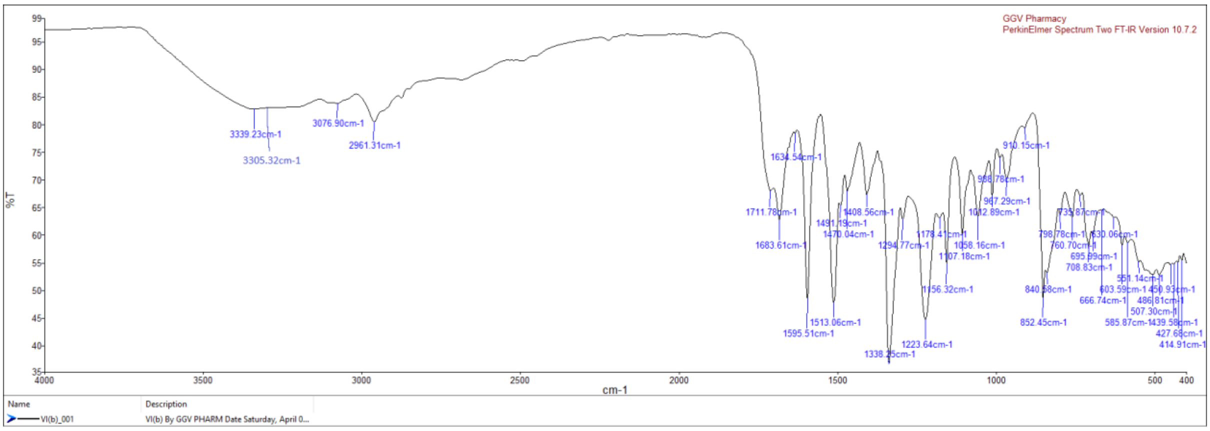

H[1]-NMR-spectra of compound VI(b)

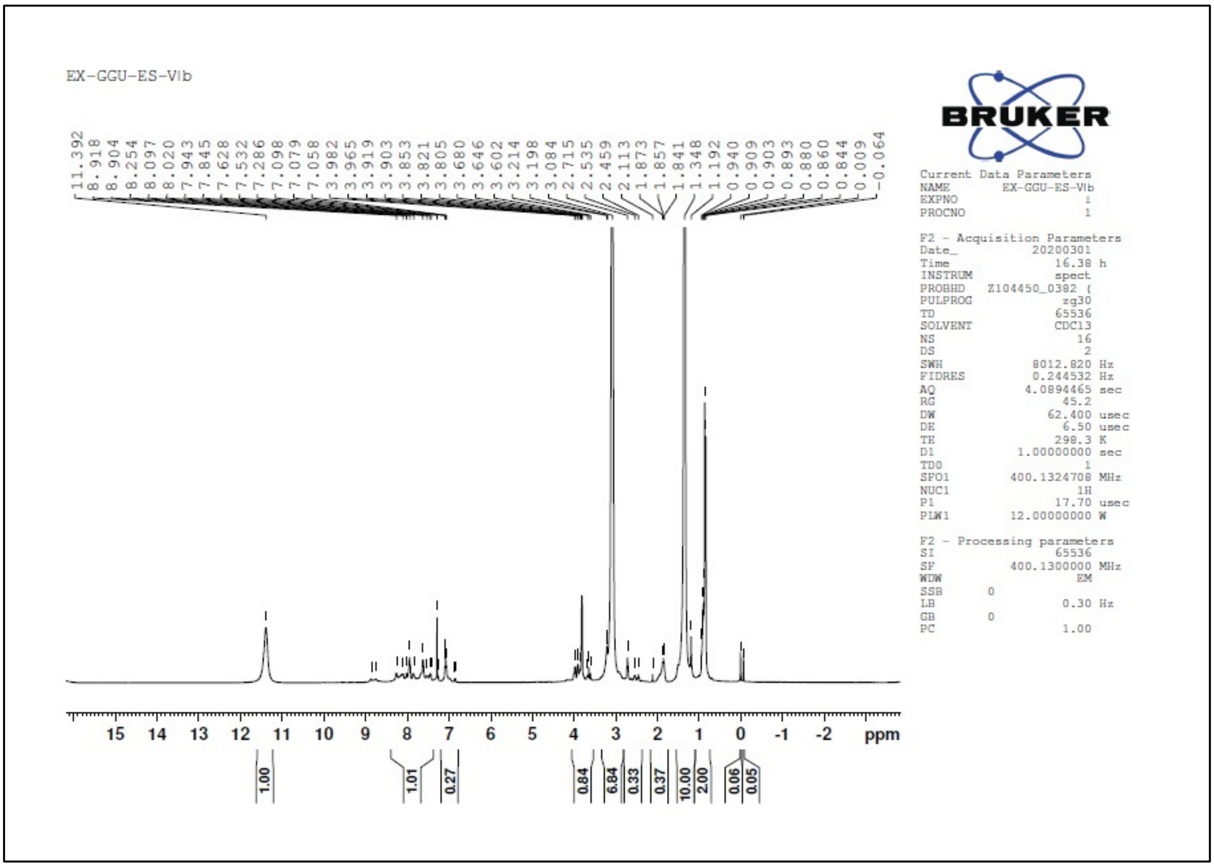

^{13}CNMR-spectra of compound VI(b)

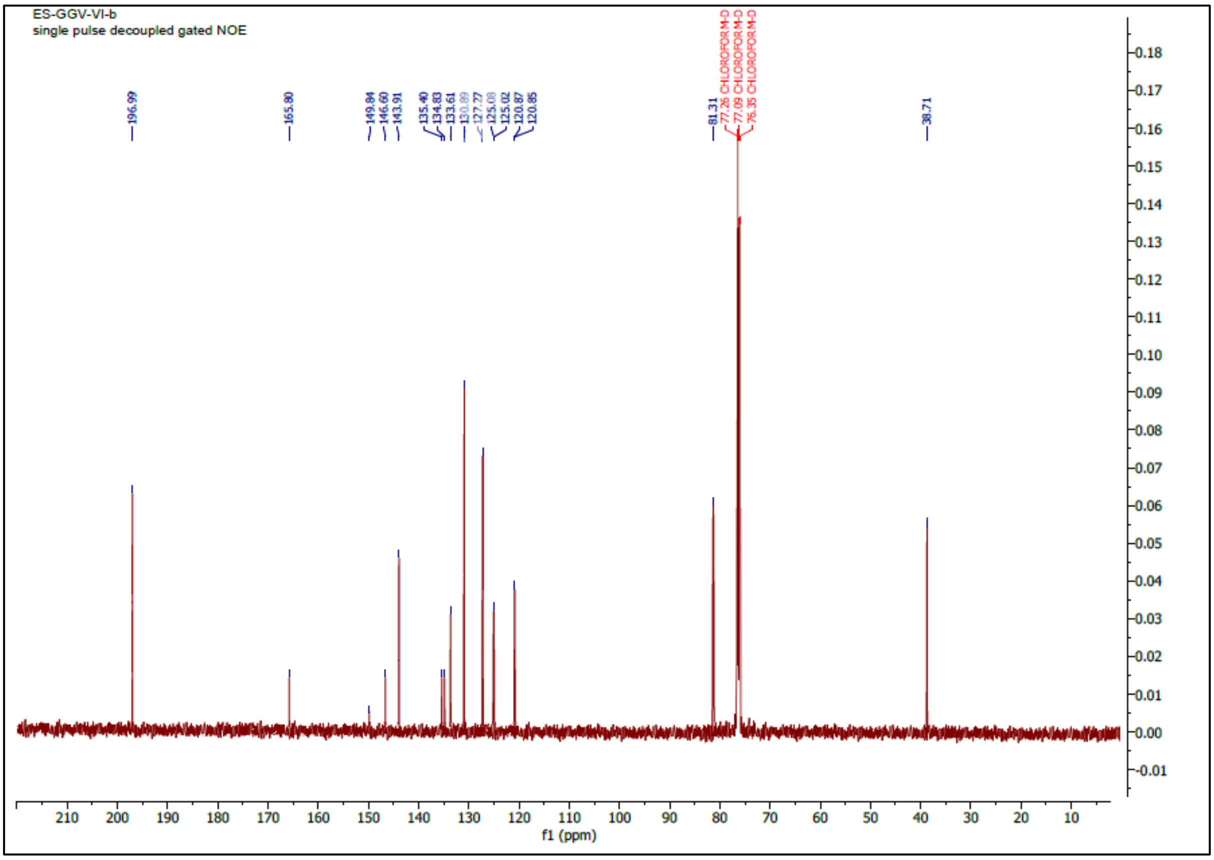

Mass-spectra of compound VI(b)

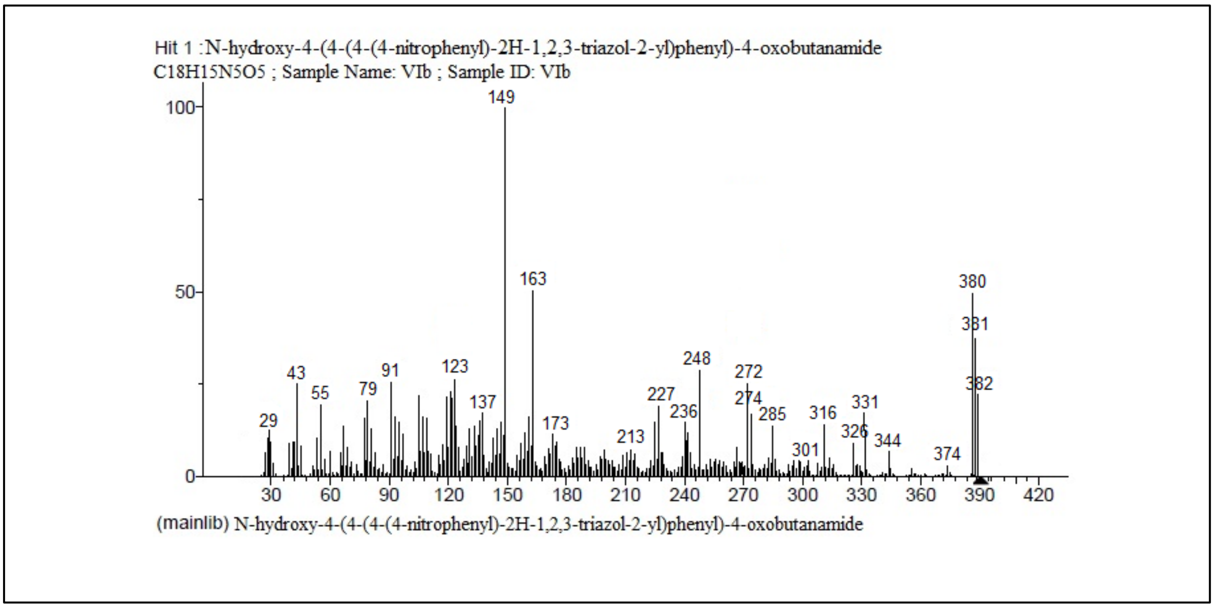

Spectral data of compound VI(c)

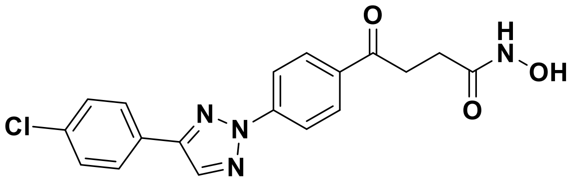

4-(4-(4-(4-chlorophenyl)-2*H*-1,2,3-triazol-2-yl)phenyl)-*N*-hydroxy-4-oxobutanamide

Chemical Formula: $C_{18}H_{15}ClN_4O_3$

Molecular Weight: 370.79

IR-spectra of compound VI(c)

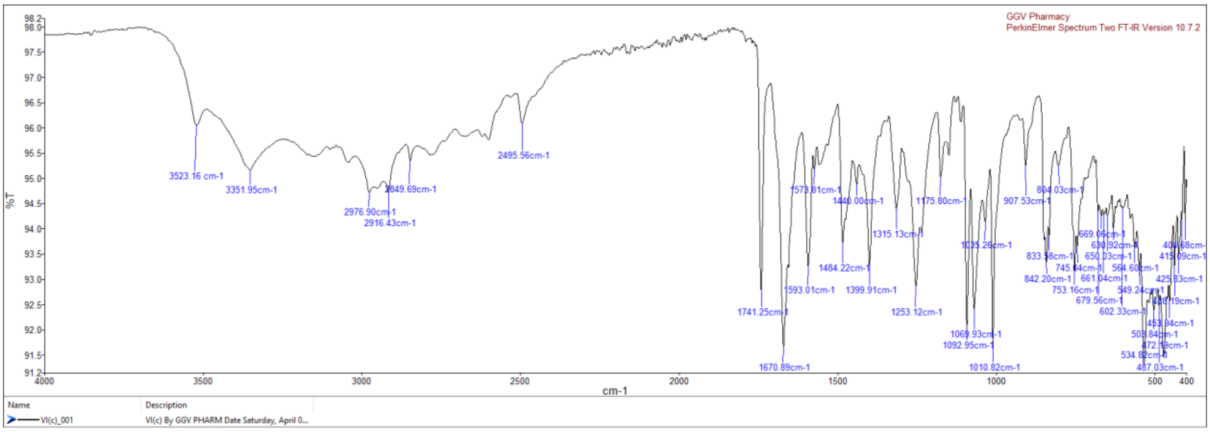

H[1]-NMR-spectra of compound VI(c)

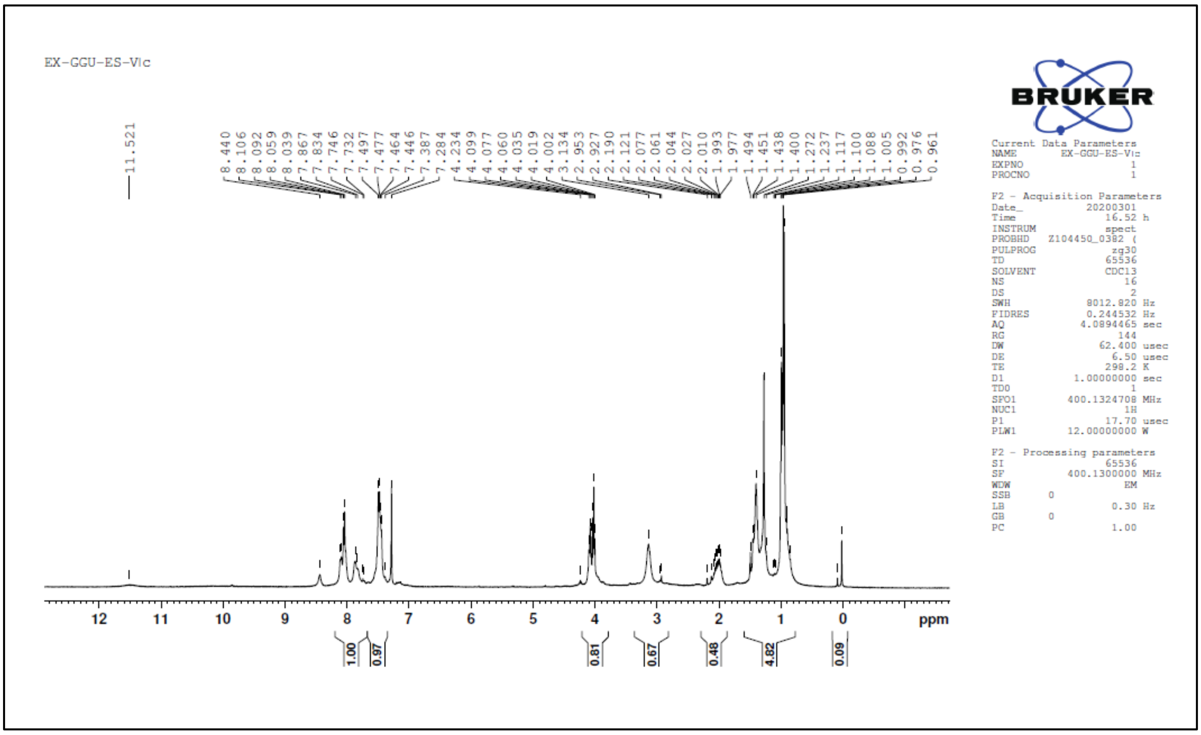

^{13}CNMR-spectra of compound VI(c)

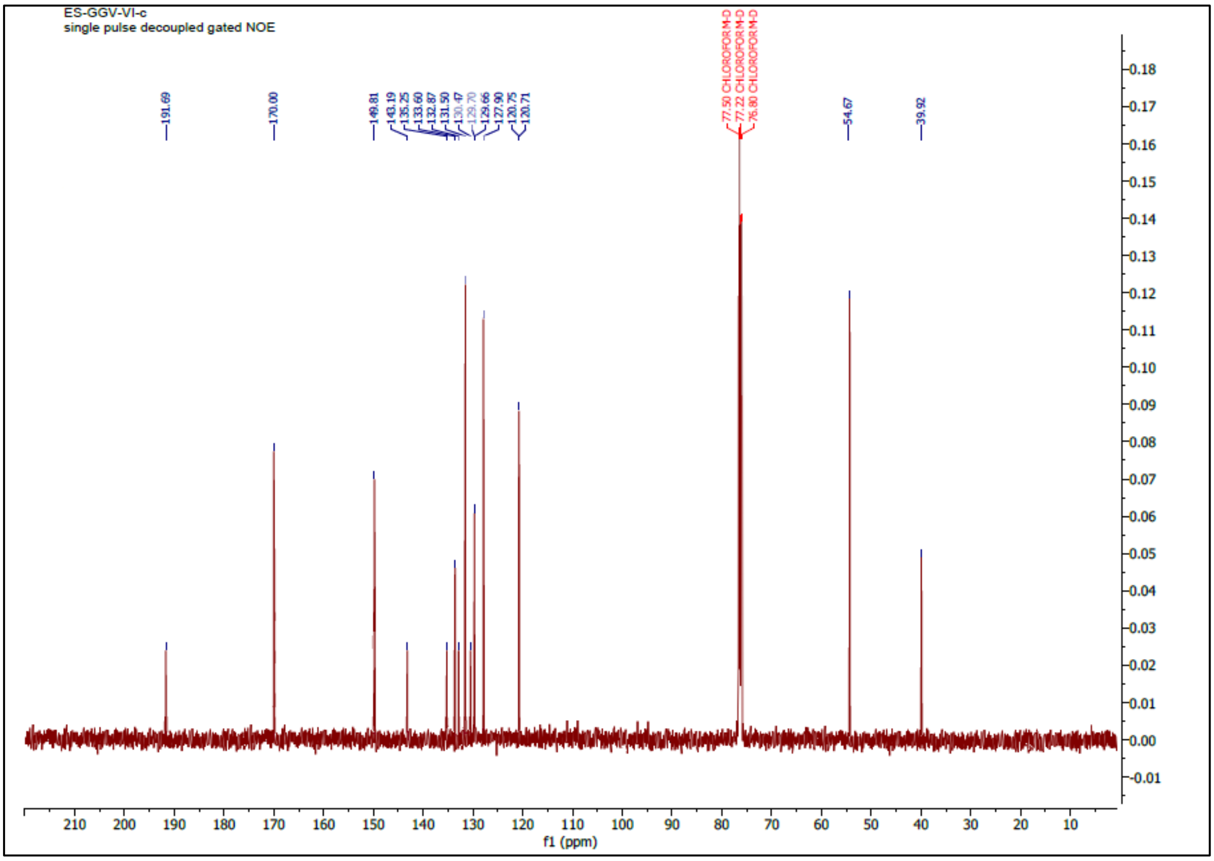

Mass-spectra of compound VI(c)

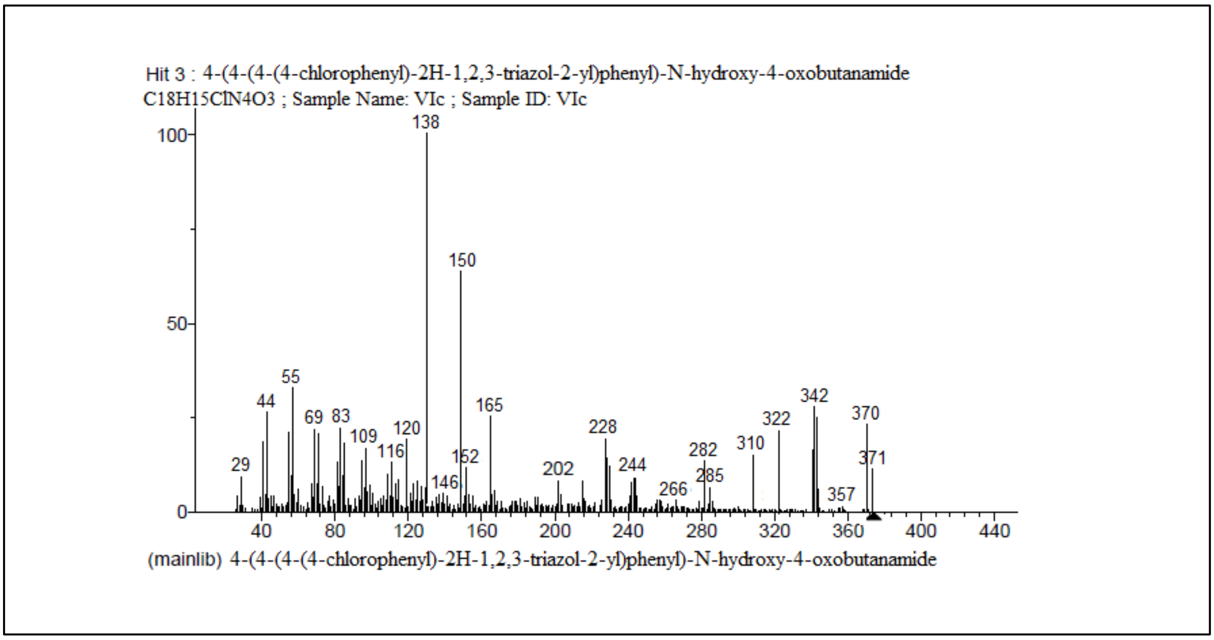

Spectral data of compound VI(d)

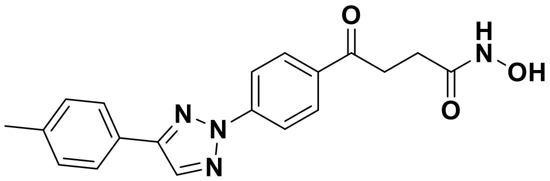

N-hydroxy-4-oxo-4-(4-(4-(*p*-tolyl)-2*H*-1,2,3-triazol-2-yl)phenyl)butanamide

Chemical Formula: $C_{19}H_{18}N_4O_3$

Molecular Weight: 350.38

IR-spectra of compound VI(d)

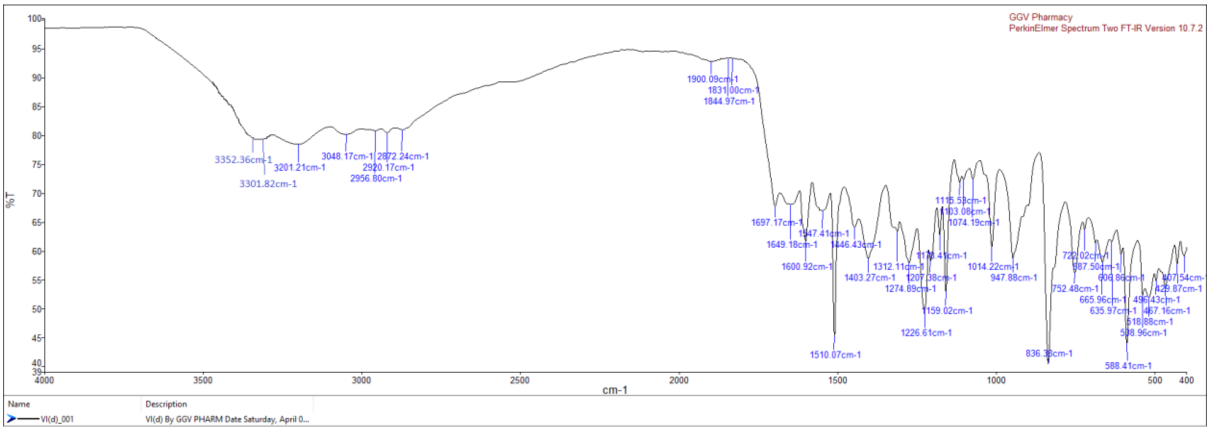

H[1]-NMR-spectra of compound VI(d)

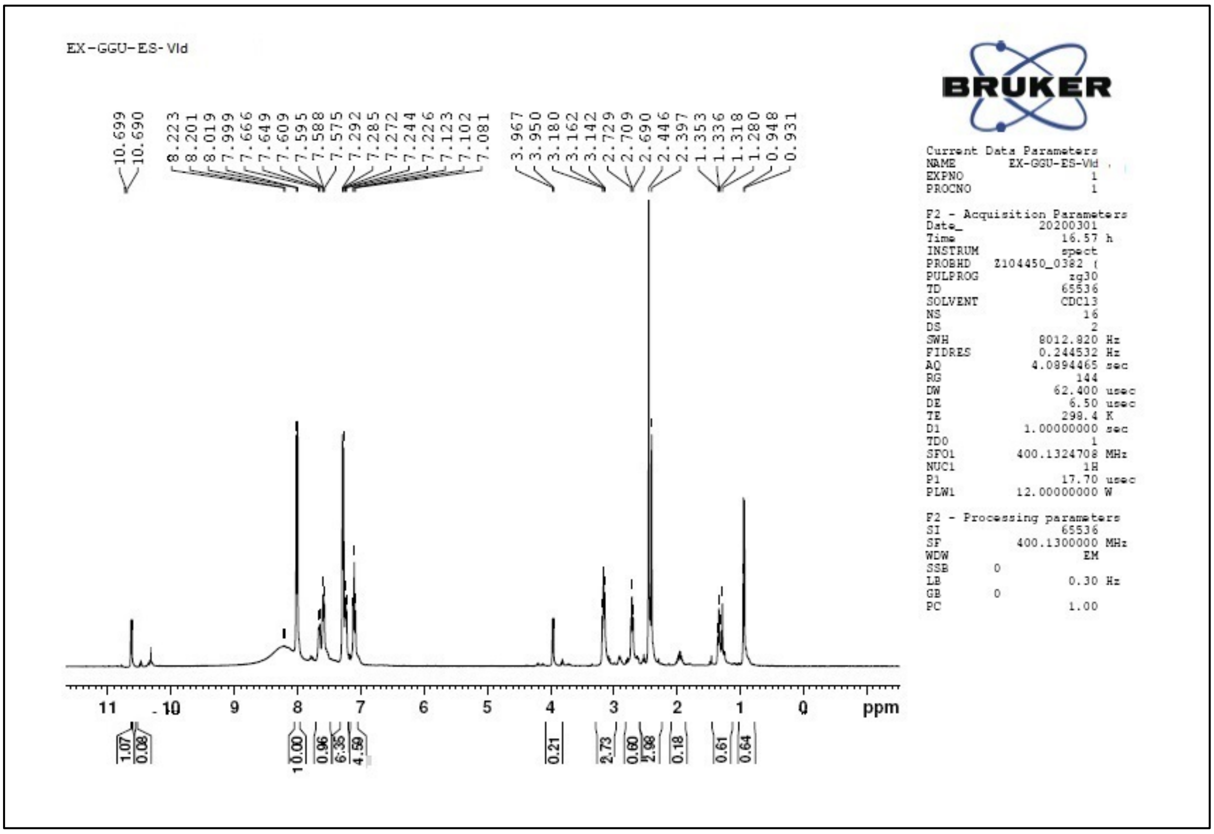

^{13C}NMR-spectra of compound VI(d)

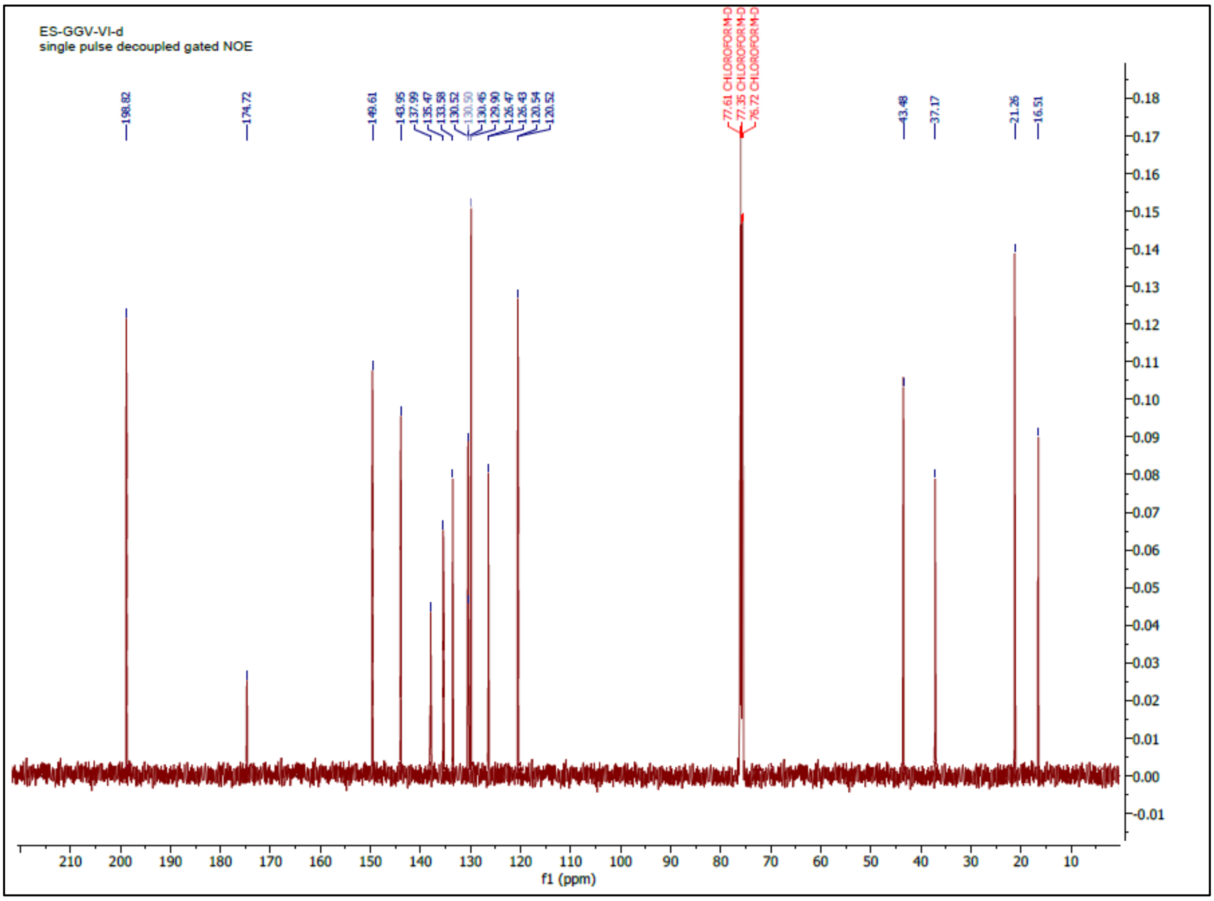

Mass-spectra of compound VI(d)

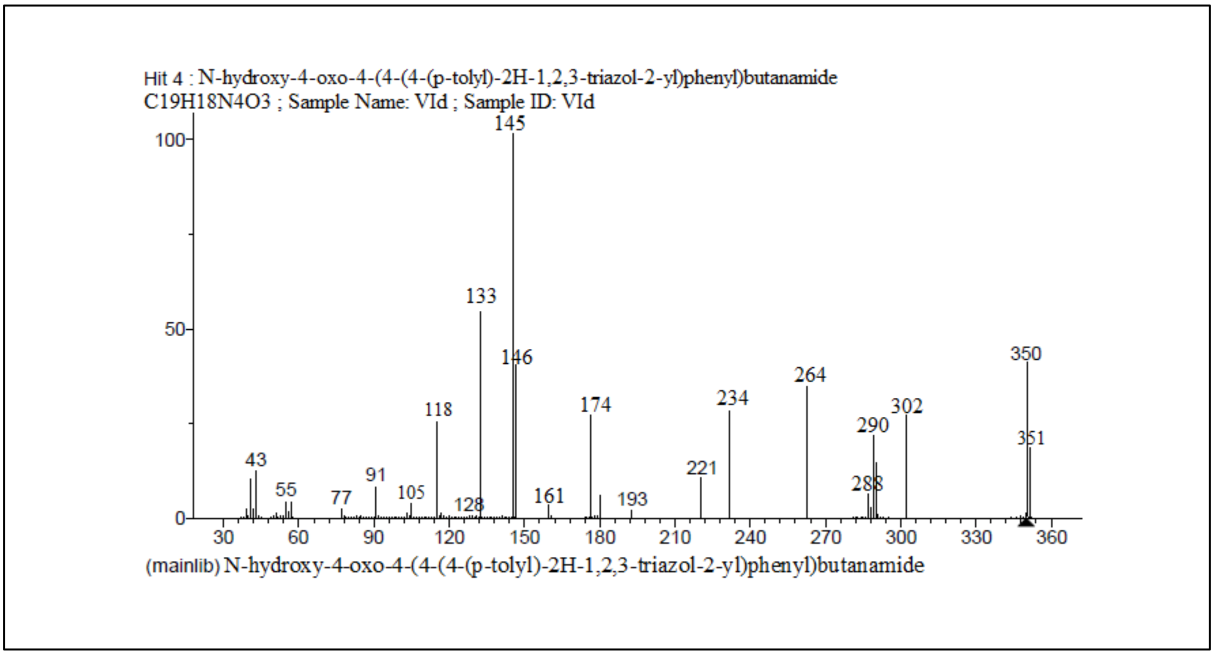

Spectral data of compound VI(e)

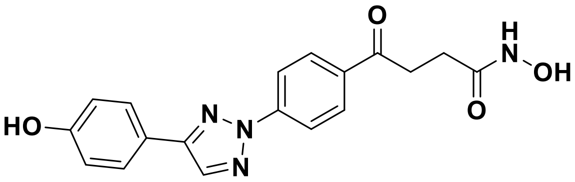

N-hydroxy-4-(4-(4-(4-hydroxyphenyl)-2*H*-1,2,3-triazol-2-yl)phenyl)-4-oxobutanamide

Chemical Formula: $C_{18}H_{16}N_4O_4$

Molecular Weight: 352.35

IR-spectra of compound VI(e)

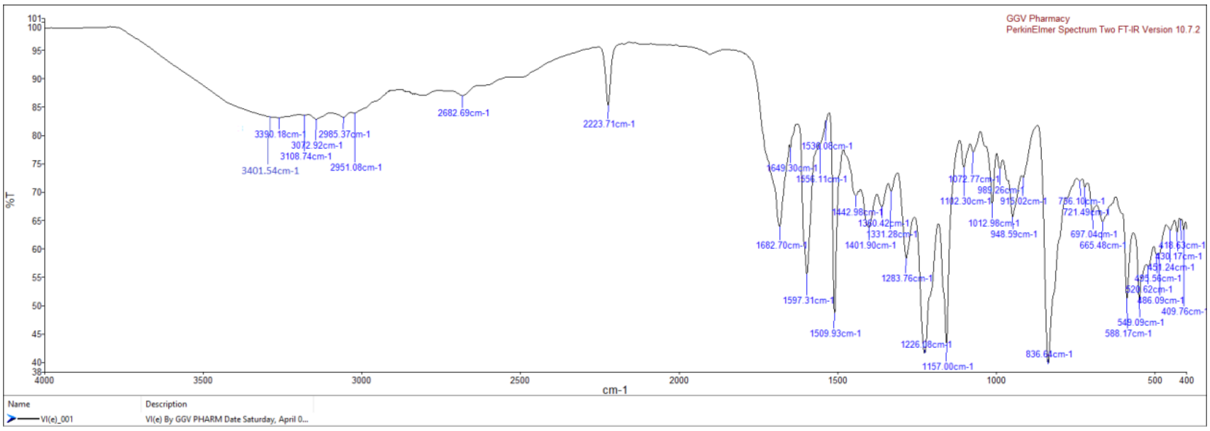

H[1]-NMR-spectra of compound VI(e)

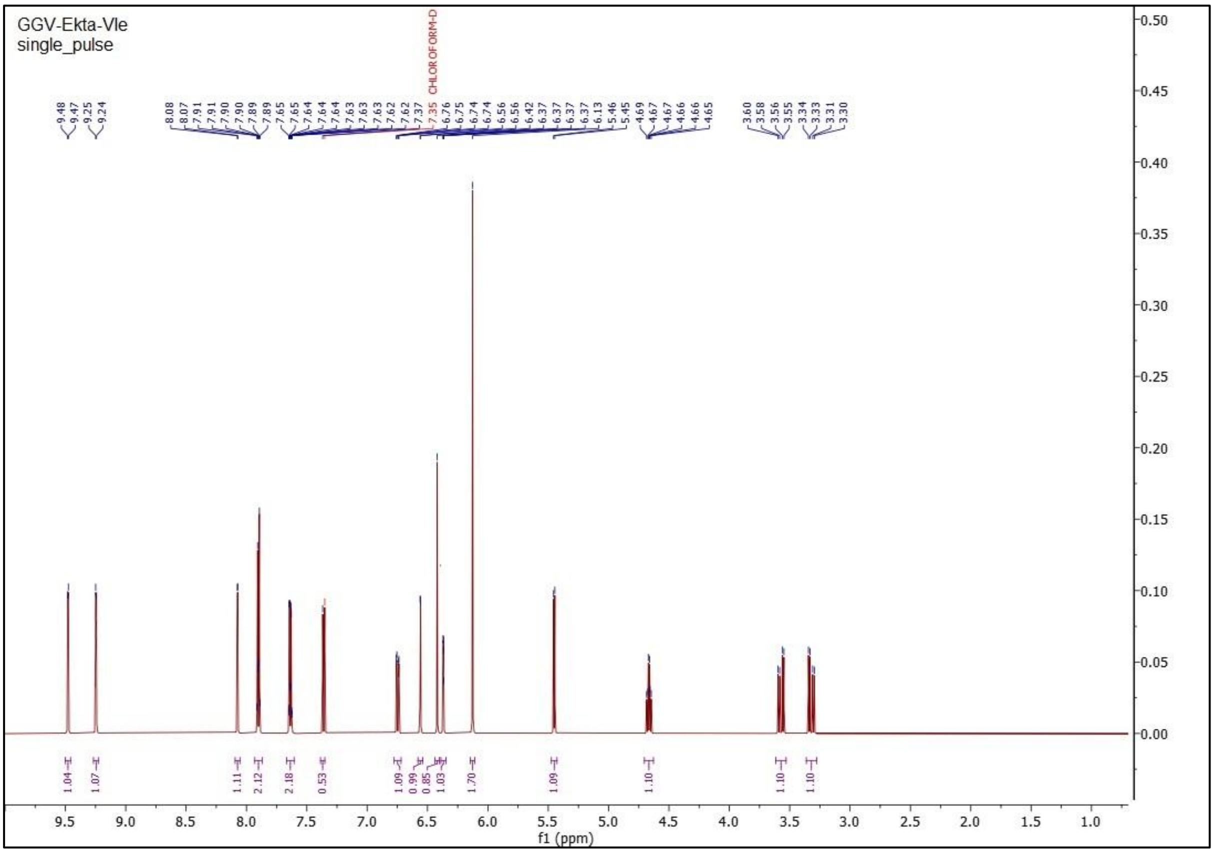

[13C]NMR-spectra of compound VI(e)

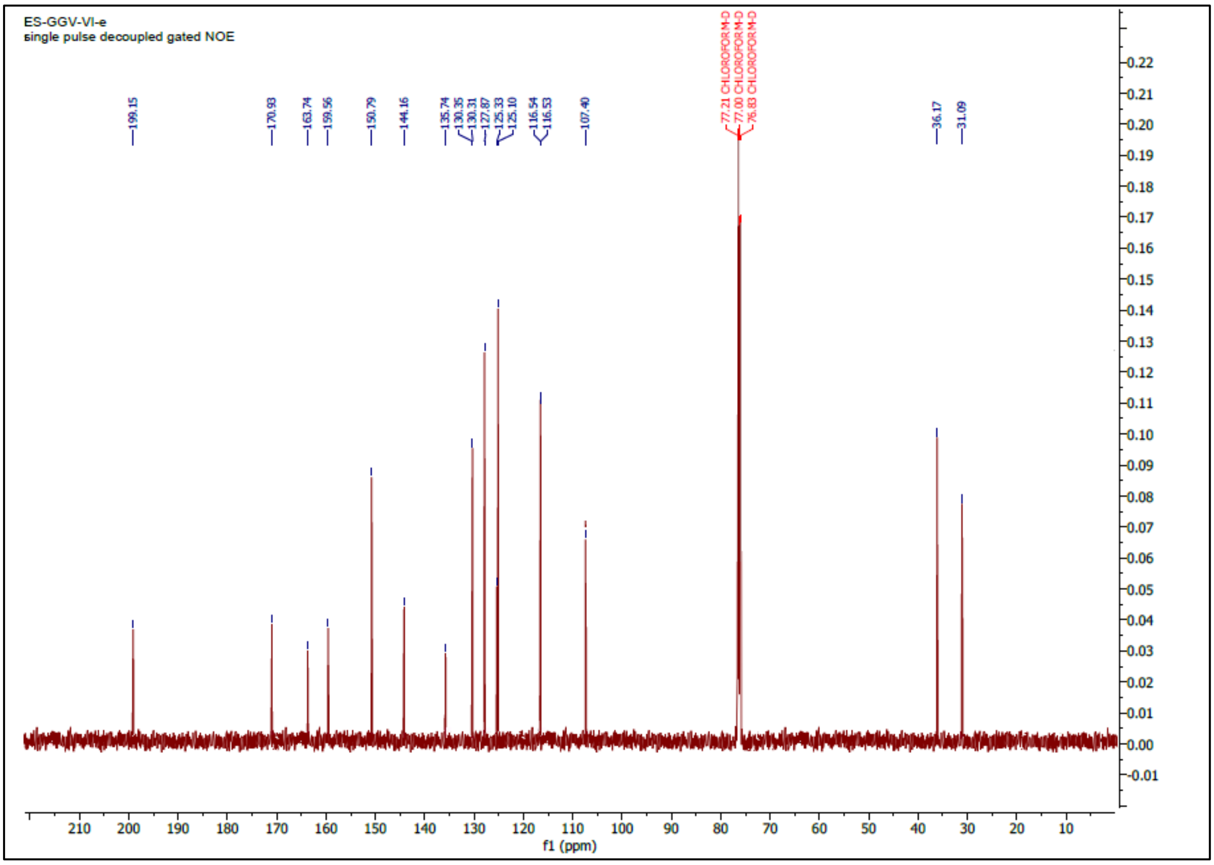

Mass-spectra of compound VI(e)

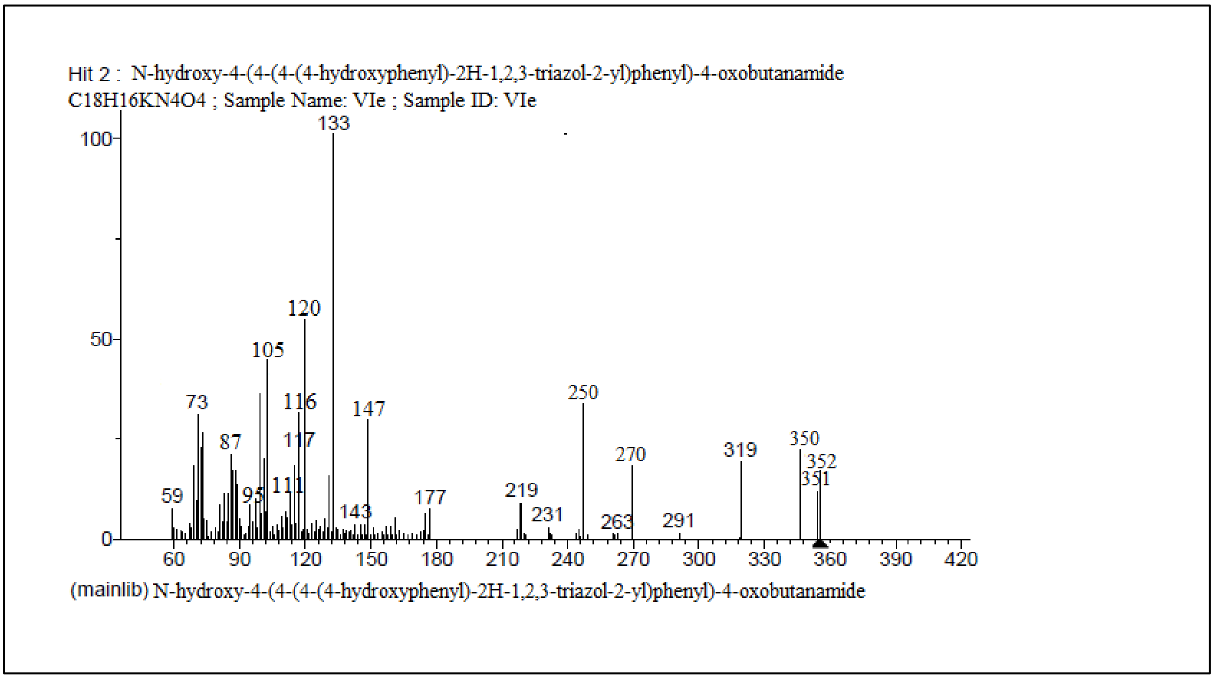

Spectral data of compound VI(f)

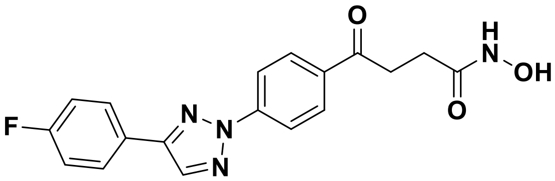

4-(4-(4-(4-fluorophenyl)-2*H*-1,2,3-triazol-2-yl)phenyl)-*N*-hydroxy-4-oxobutanamide

Chemical Formula: $C_{18}H_{15}FN_4O_3$

Molecular Weight: 354.34

IR-spectra of compound VI(f)

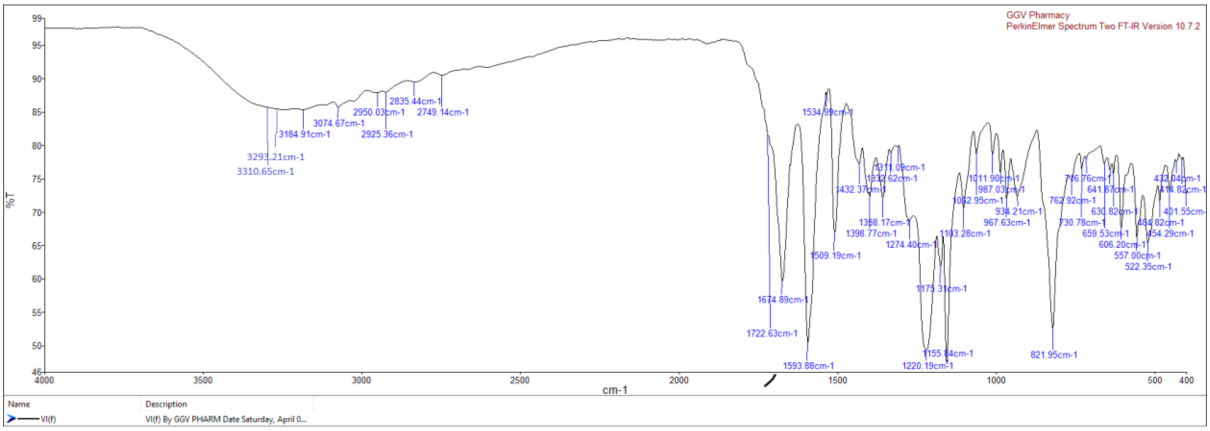

H[1]-NMR-spectra of compound VI(f)

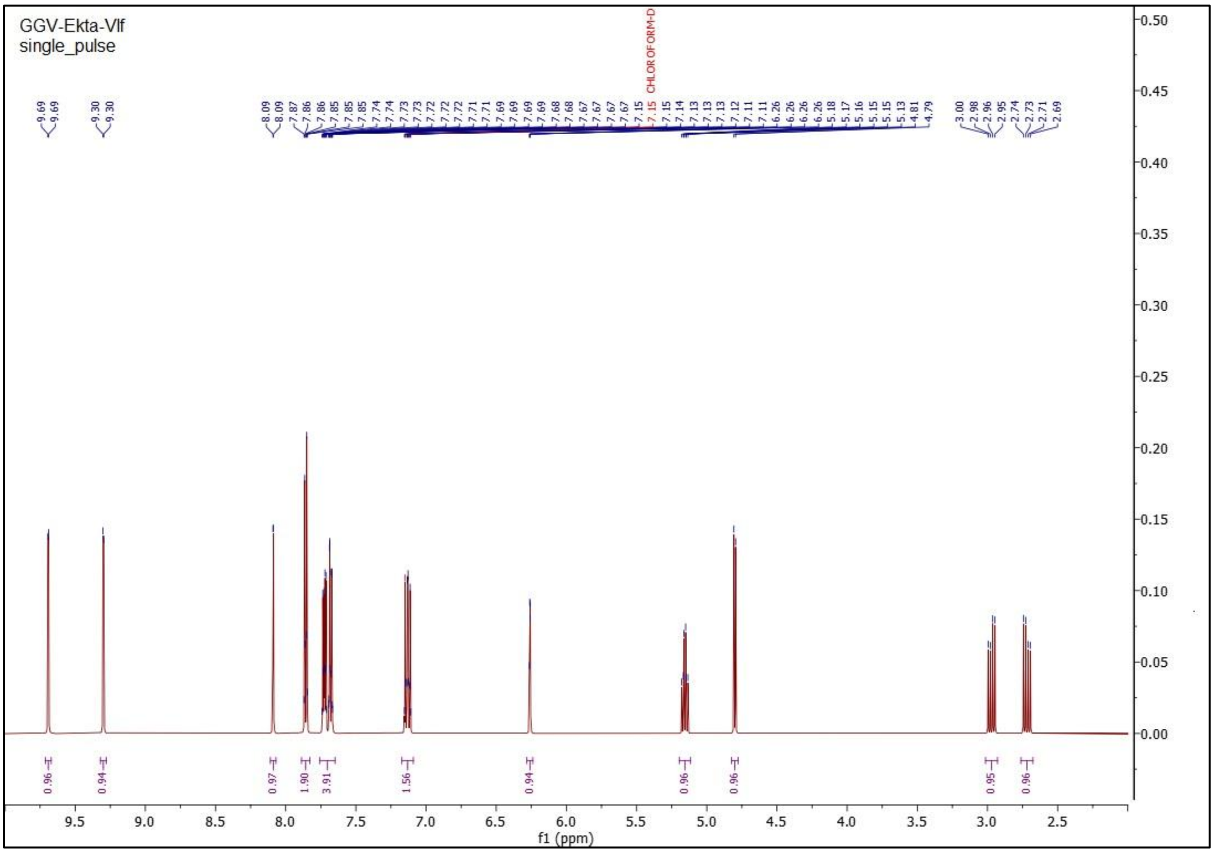

^{13C}NMR-spectra of compound VI(f)

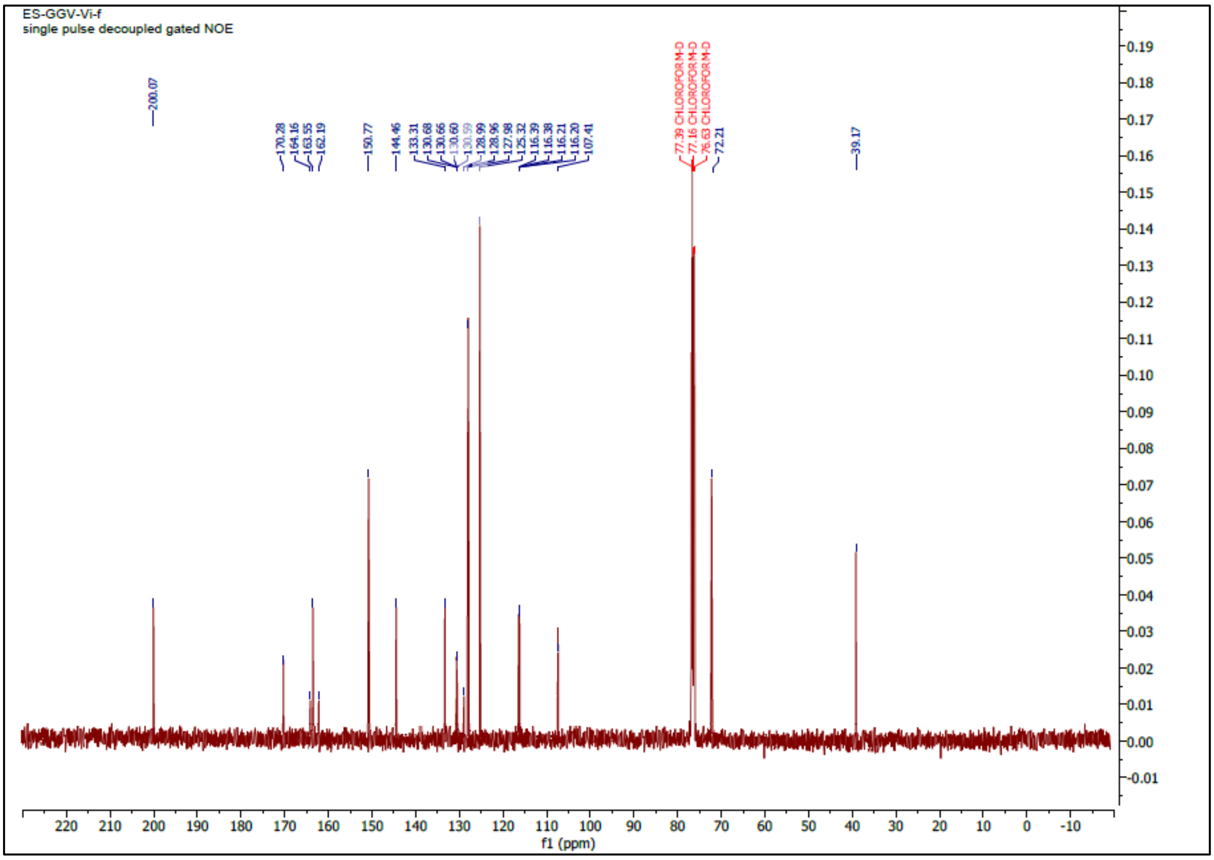

Mass-spectra of compound VI(f)

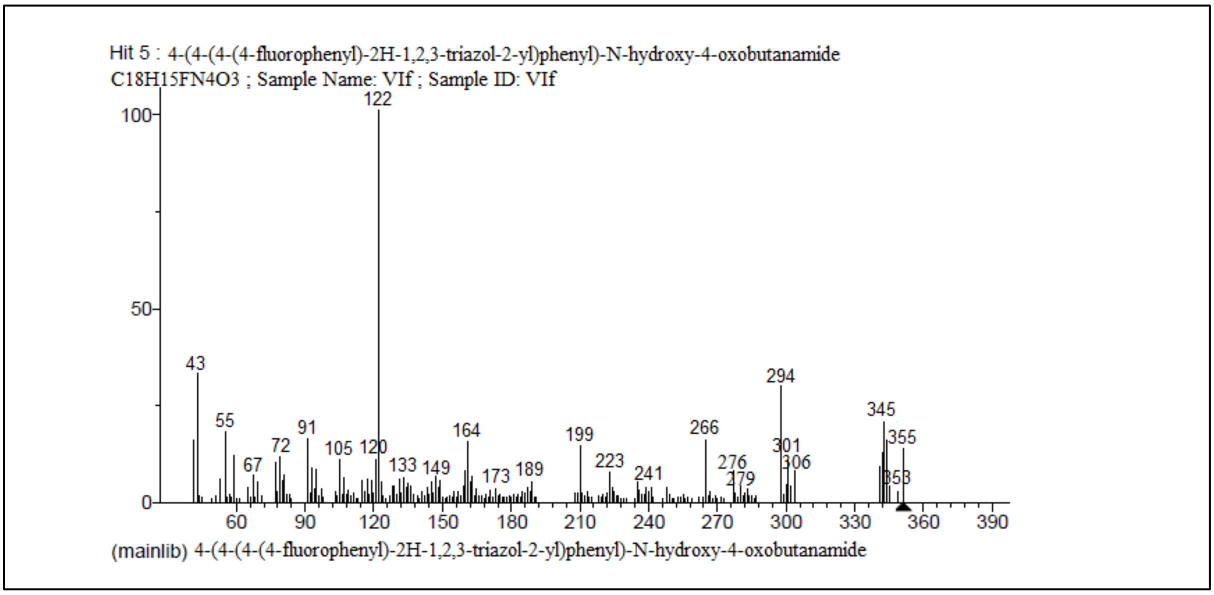

Spectral data of compound VI(g)

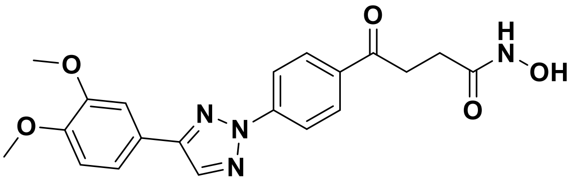

4-(4-(4-(3,4-dimethoxyphenyl)-2*H*-1,2,3-triazol-2-yl)phenyl)-*N*-hydroxy-4-oxobutanamide

Chemical Formula: $C_{20}H_{20}N_4O_5$

Molecular Weight: 396.40

IR-spectra of compound VI(g)

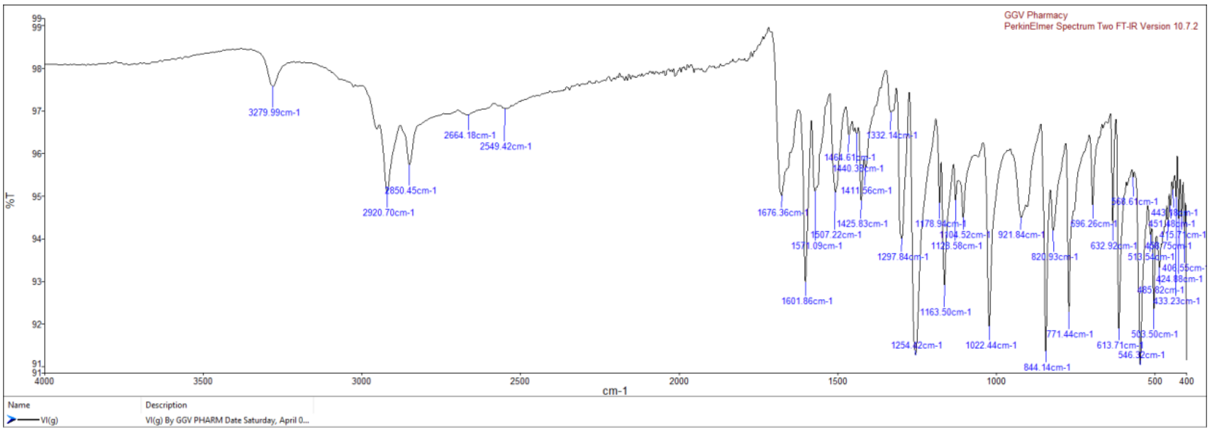

H[1]-NMR-spectra of compound VI(g)

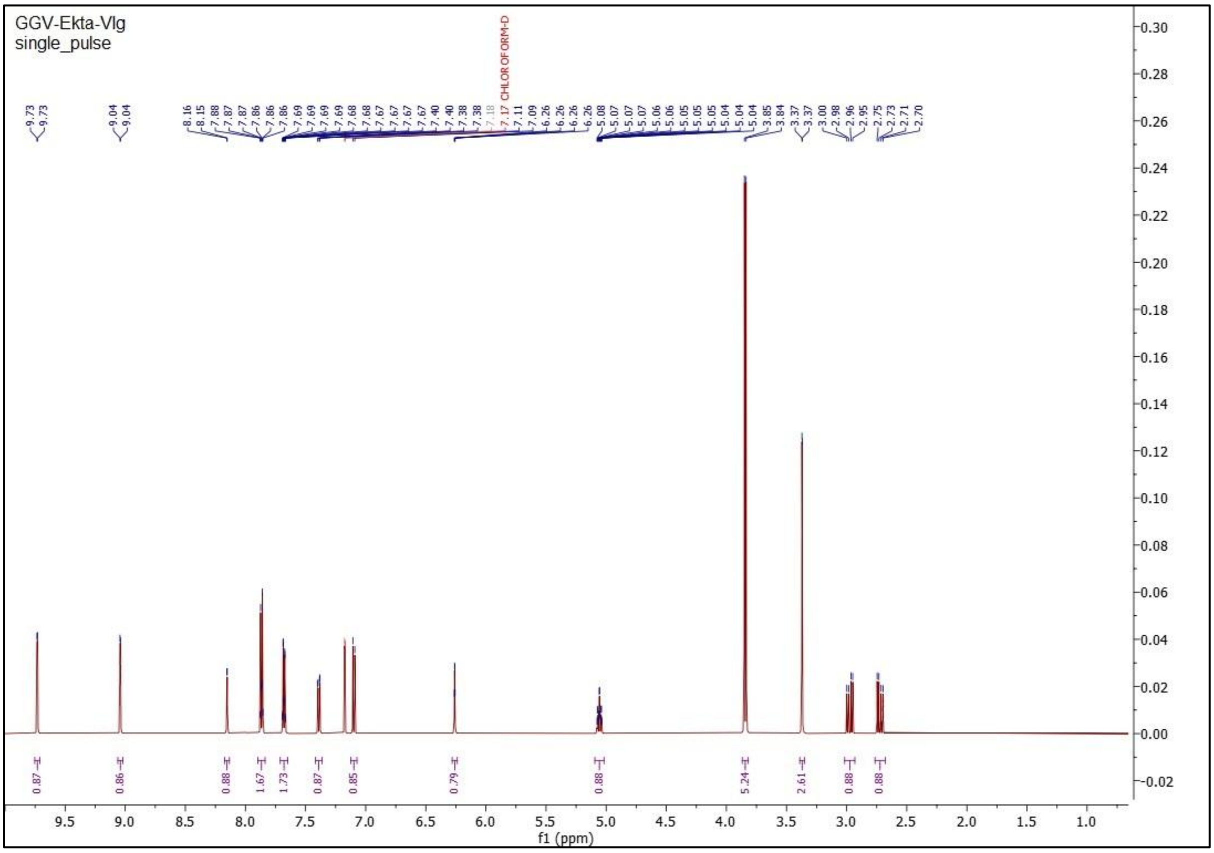

^{13C}NMR-spectra of compound VI(g)

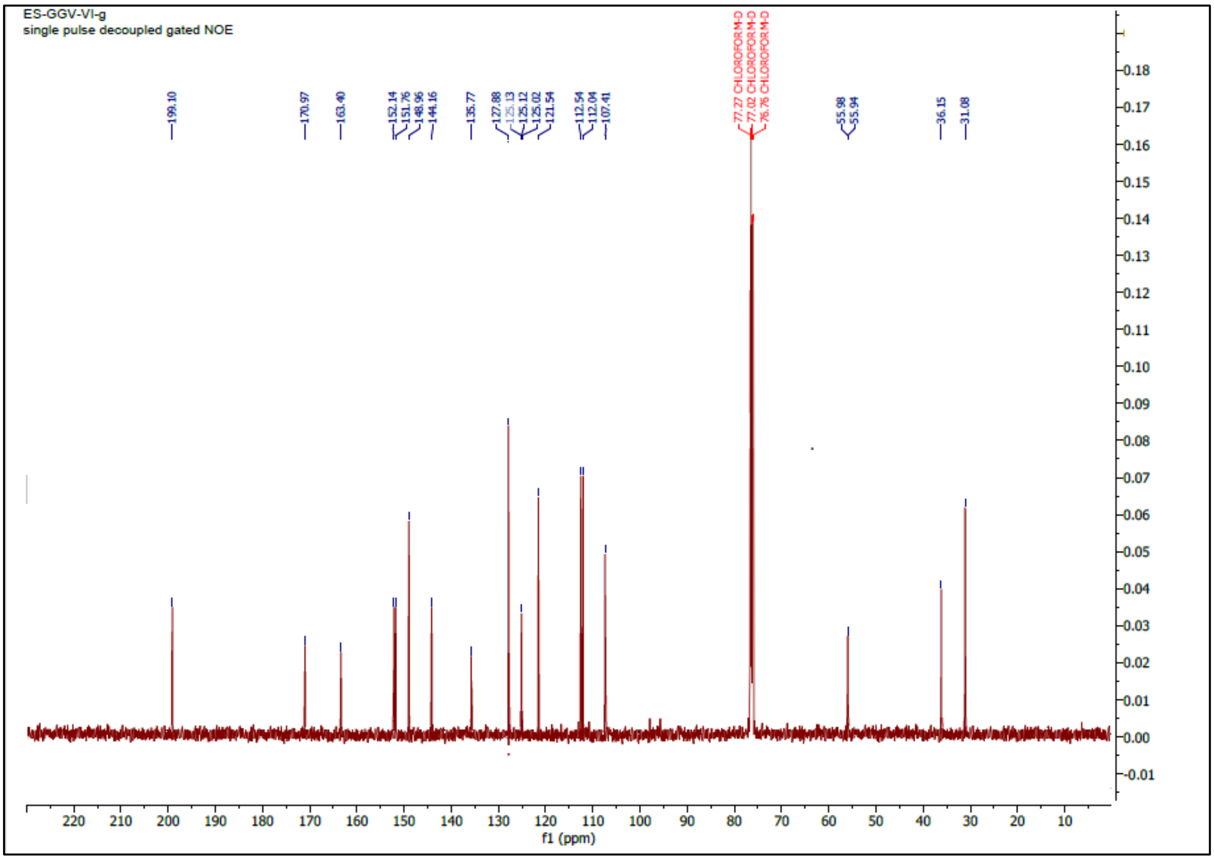

Mass-spectra of compound VI(g)

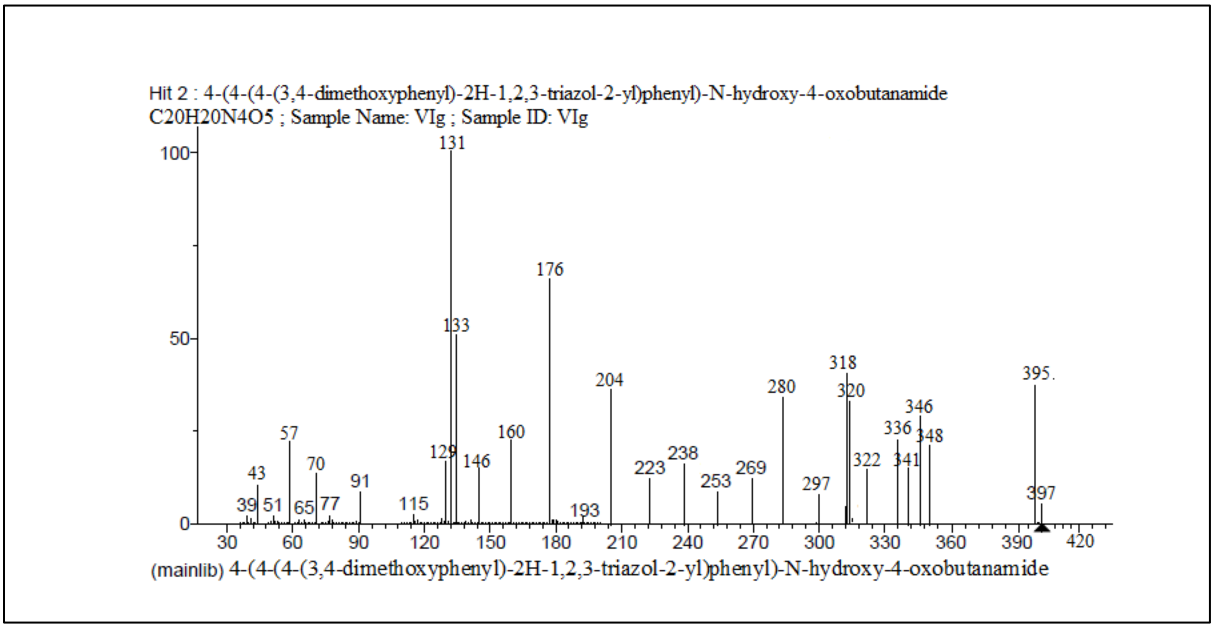

Spectral data of compound VI(h)

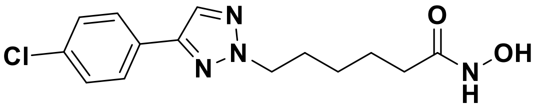

6-(4-(4-chlorophenyl)-2*H*-1,2,3-triazol-2-yl)-*N*-hydroxyhexanamide
Chemical Formula: $C_{14}H_{17}ClN_4O_2$
Molecular Weight: 308.77

IR-spectra of compound VI(h)

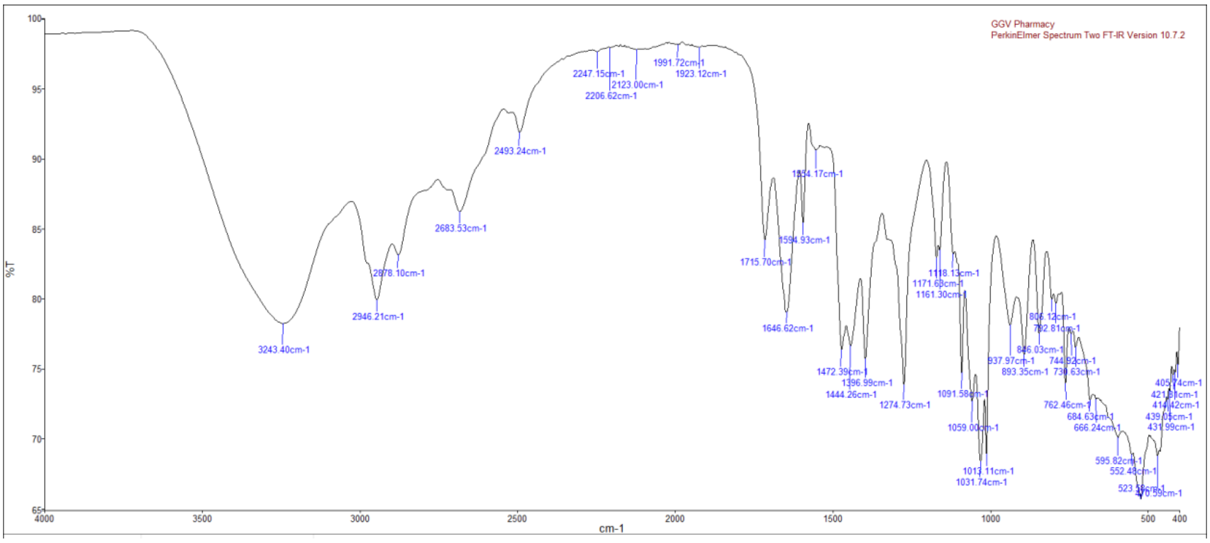

H[1]-NMR-spectra of compound VI(h)

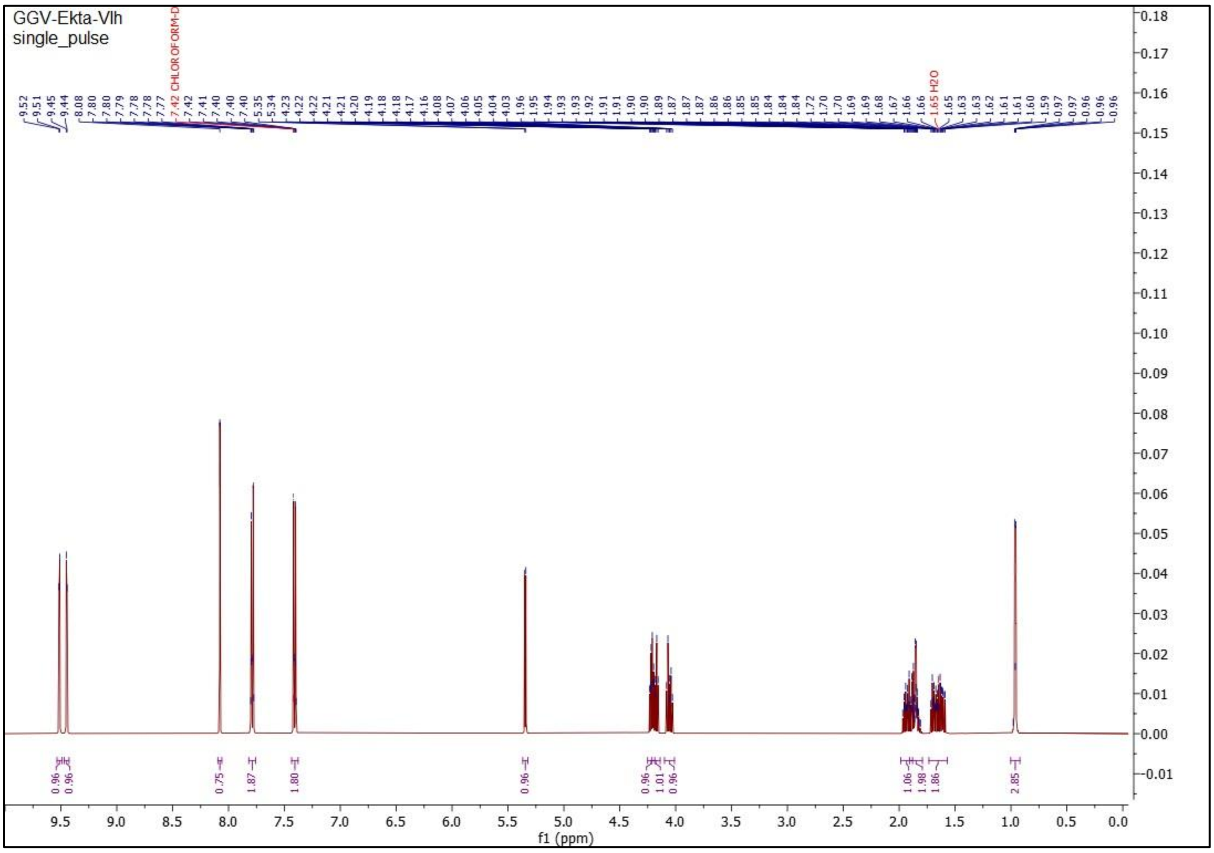

^{13}C NMR-spectra of compound VI(h)

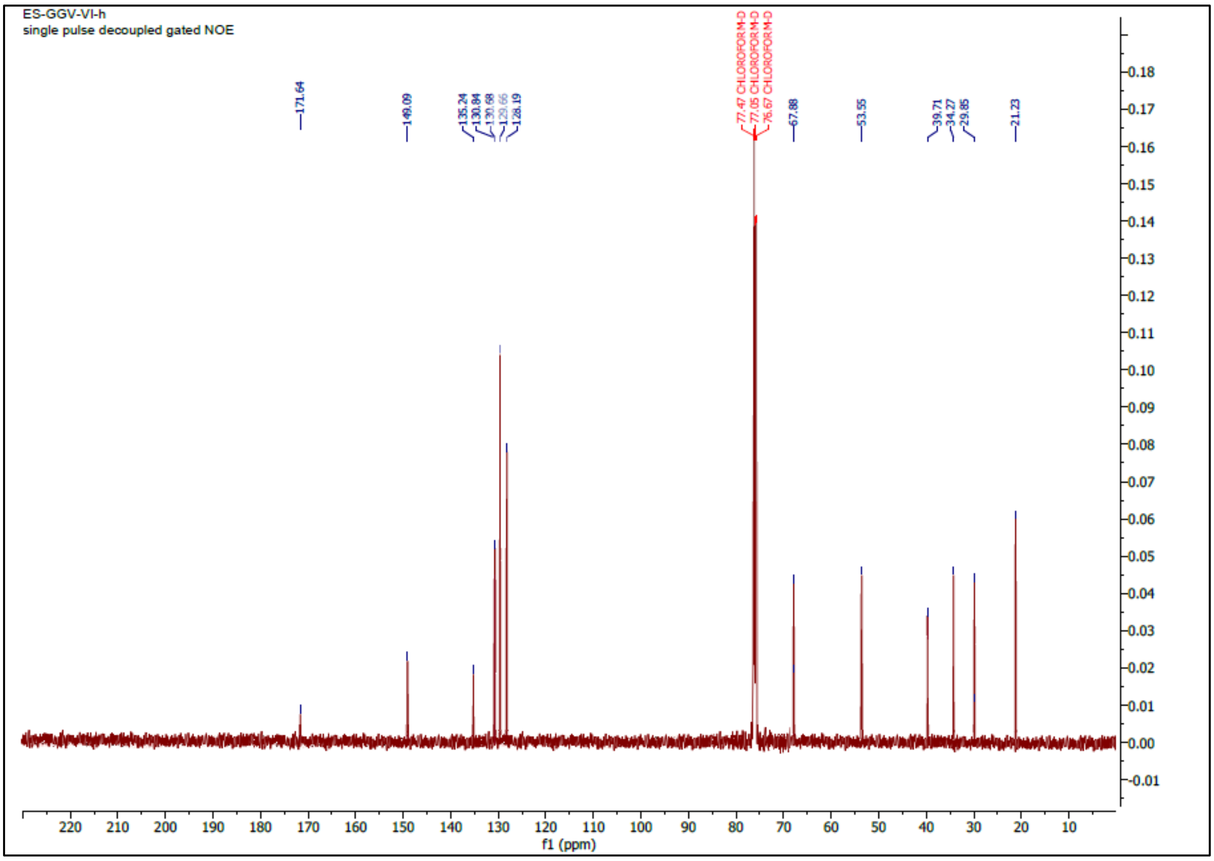

Mass-spectra of compound VI(h)

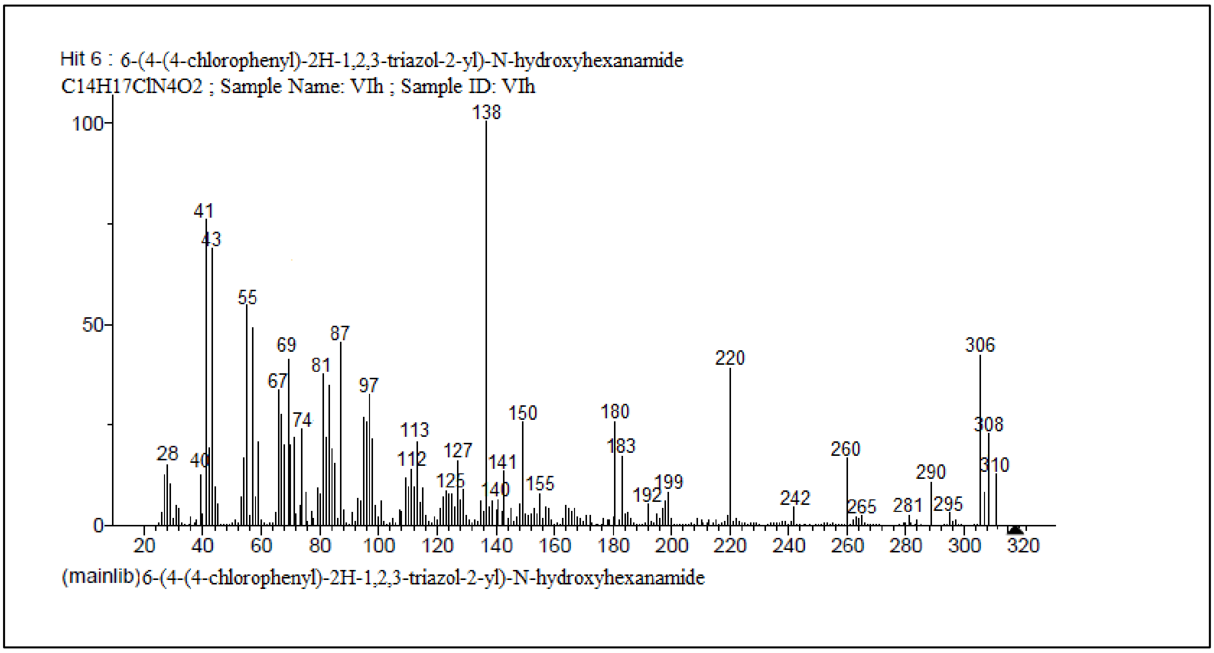

Spectral data of compound VI(i)

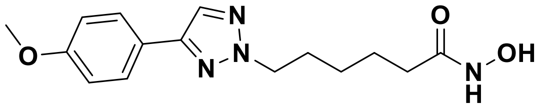

N-hydroxy-6-(4-(4-methoxyphenyl)-2*H*-1,2,3-triazol-2-yl)hexanamide
Chemical Formula: $C_{15}H_{20}N_4O_3$
Molecular Weight: 304.35

IR-spectra of compound VI(i)

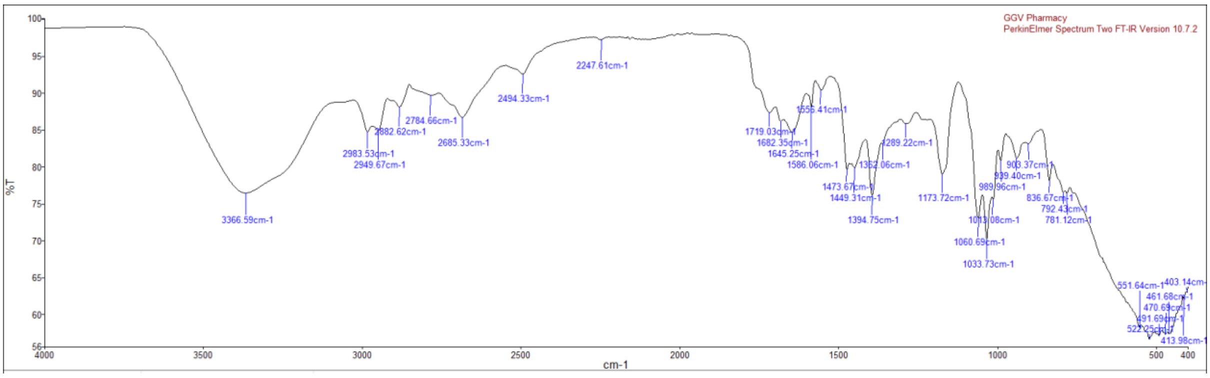

H[1]-NMR-spectra of compound VI(i)

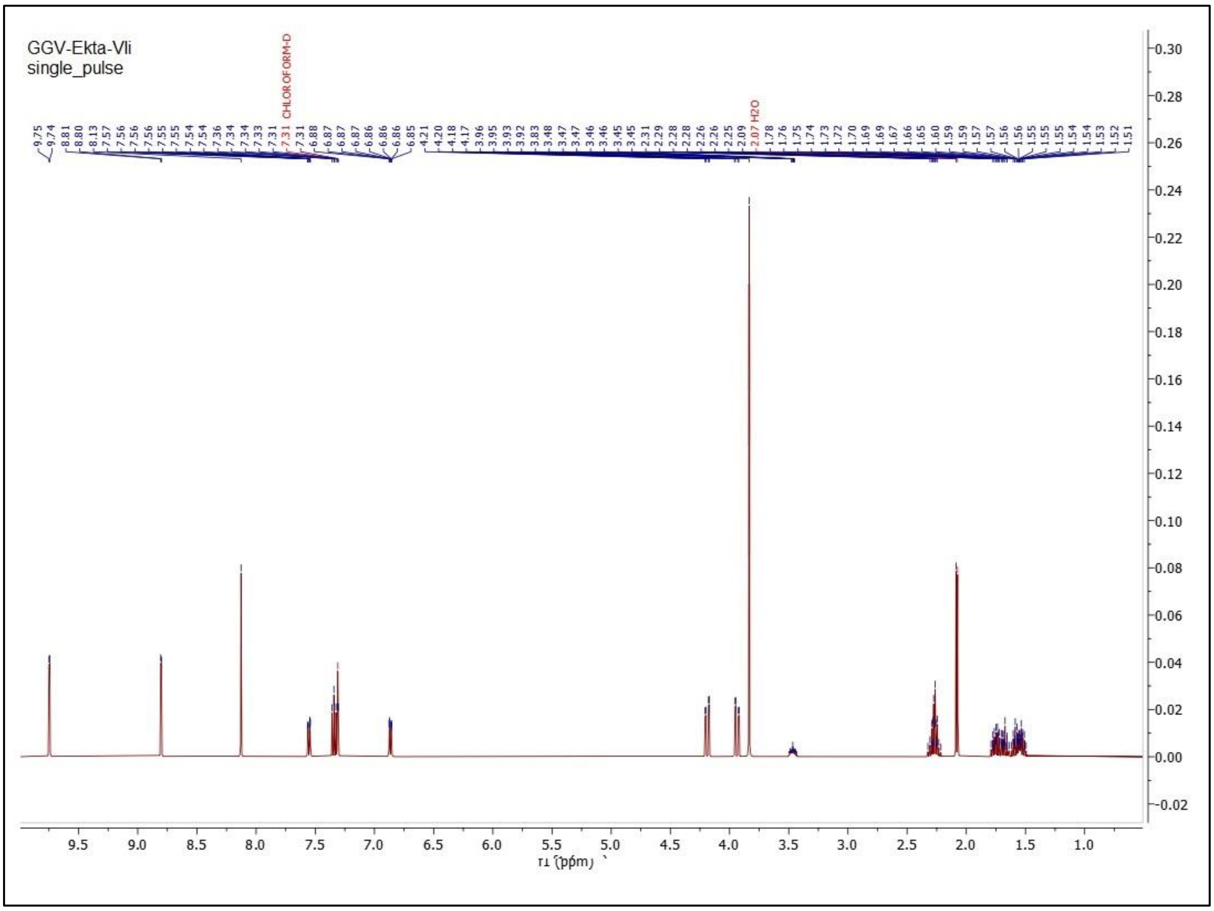

^{13}CNMR-spectra of compound VI(i)

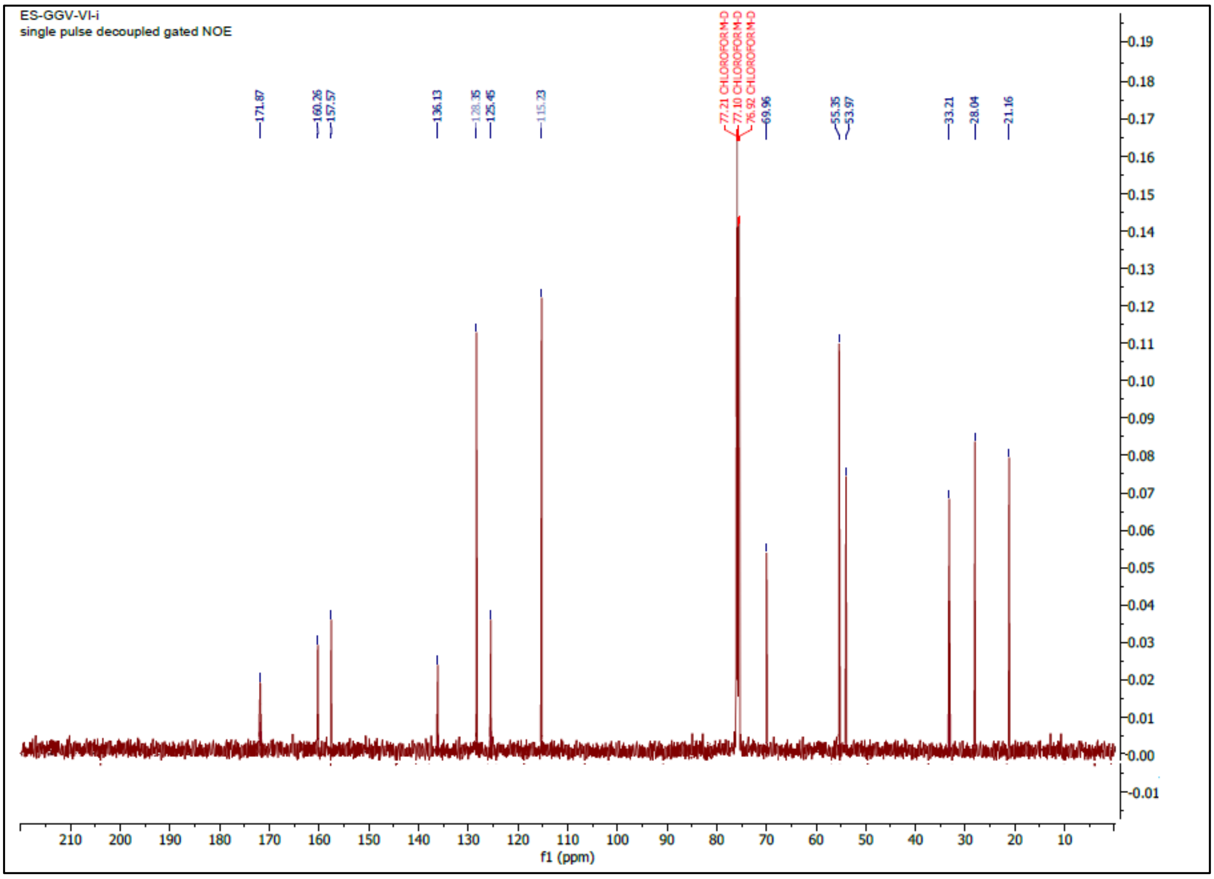

Mass-spectra of compound VI(i)

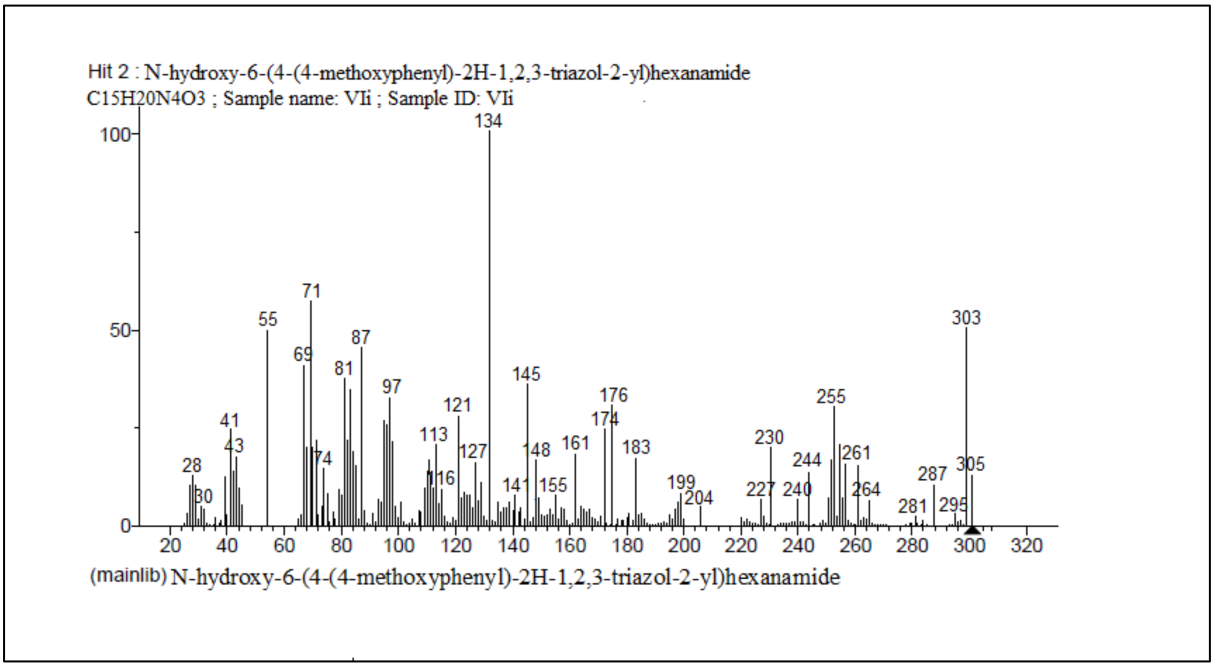

Spectral data of compound VI(j)

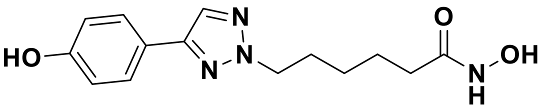

N-hydroxy-6-(4-(4-hydroxyphenyl)-2*H*-1,2,3-triazol-2-yl)hexanamide
Chemical Formula: $C_{14}H_{18}N_4O_3$
Molecular Weight: 290.32

IR-spectra of compound VI(j)

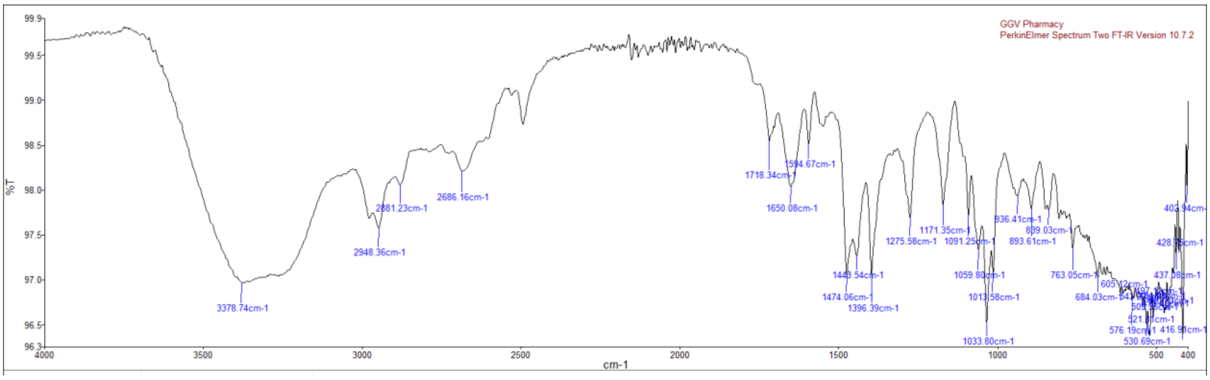

H[1]-NMR-spectra of compound VI(j)

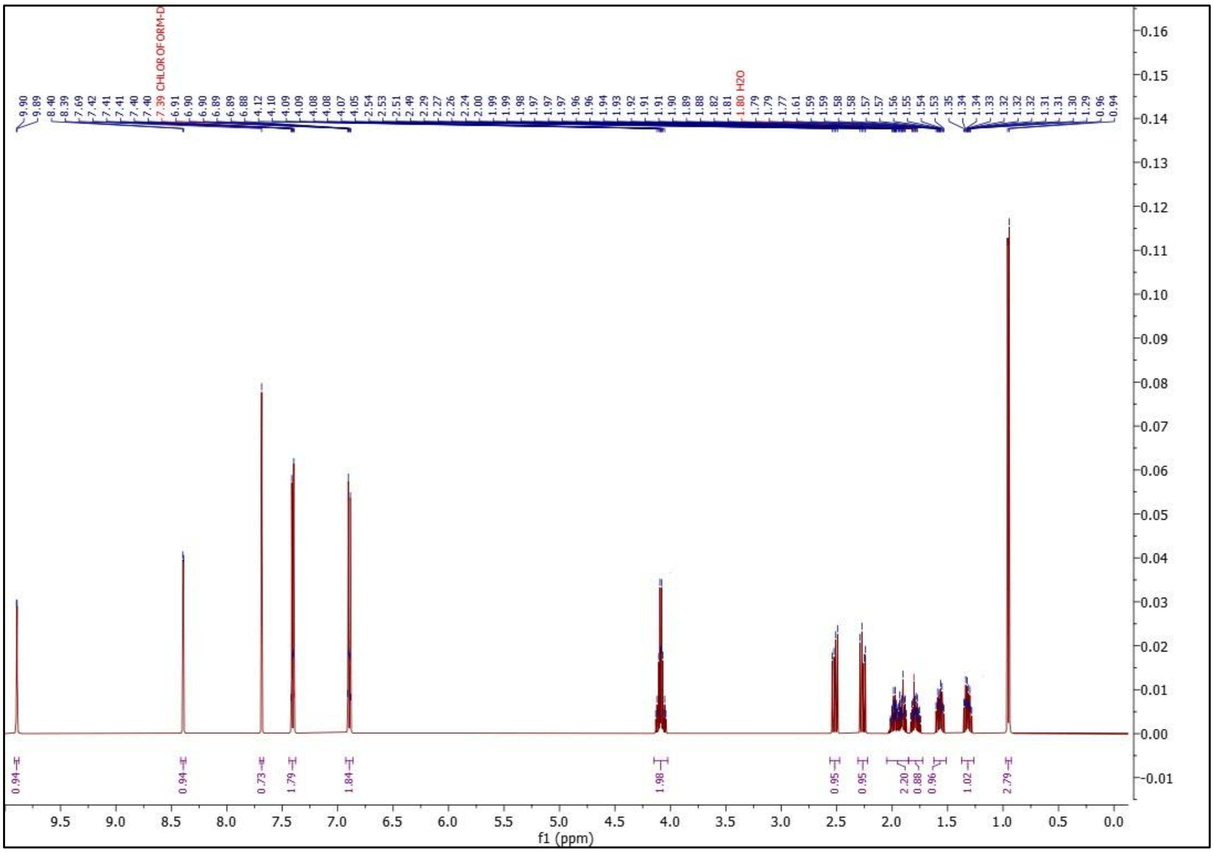

^{13C}NMR-spectra of compound VI(j)

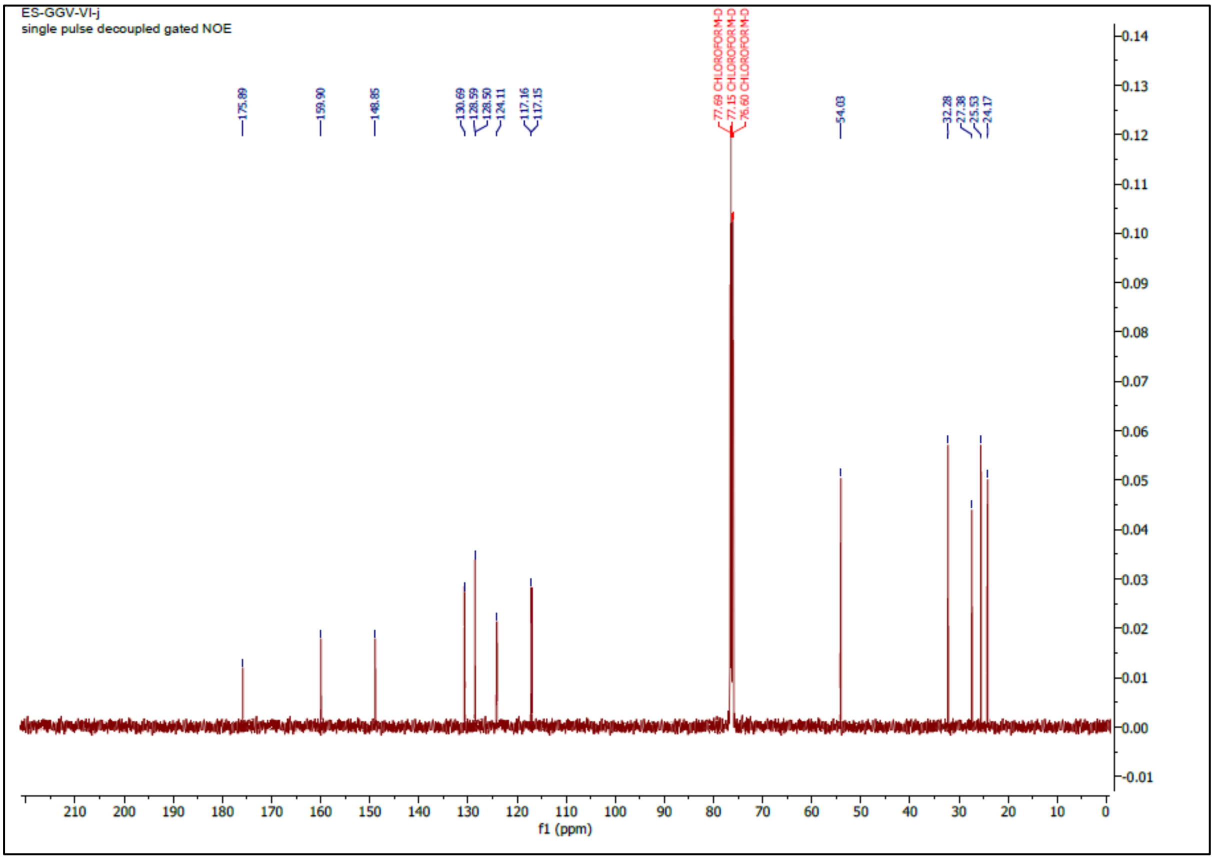

Mass-spectra of compound VI(j)

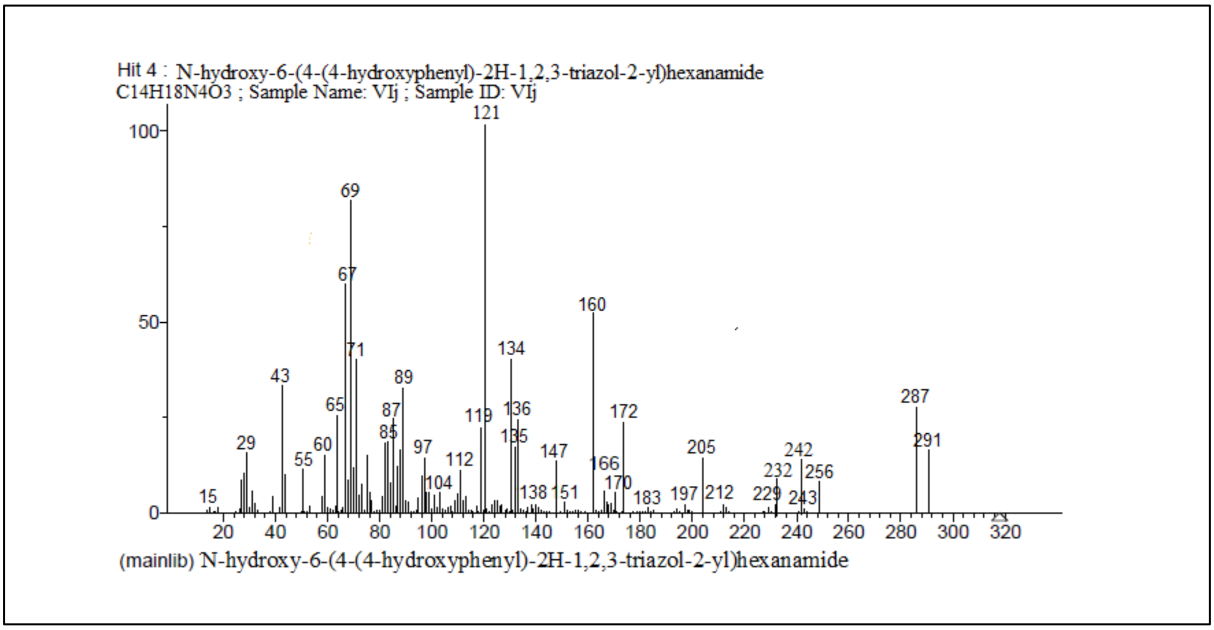

Spectral data of compound VI(k)

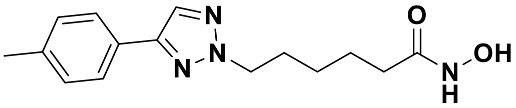

N-hydroxy-6-(4-(*p*-tolyl)-2*H*-1,2,3-triazol-2-yl)hexanamide
Chemical Formula: $C_{15}H_{20}N_4O_2$
Molecular Weight: 288.35

IR-spectra of compound VI(k)

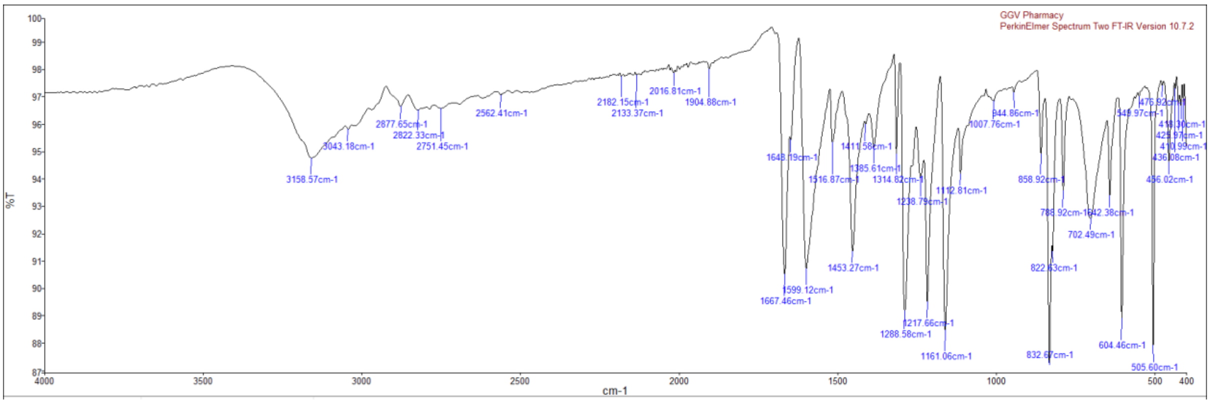

H[1]-NMR-spectra of compound VI(k)

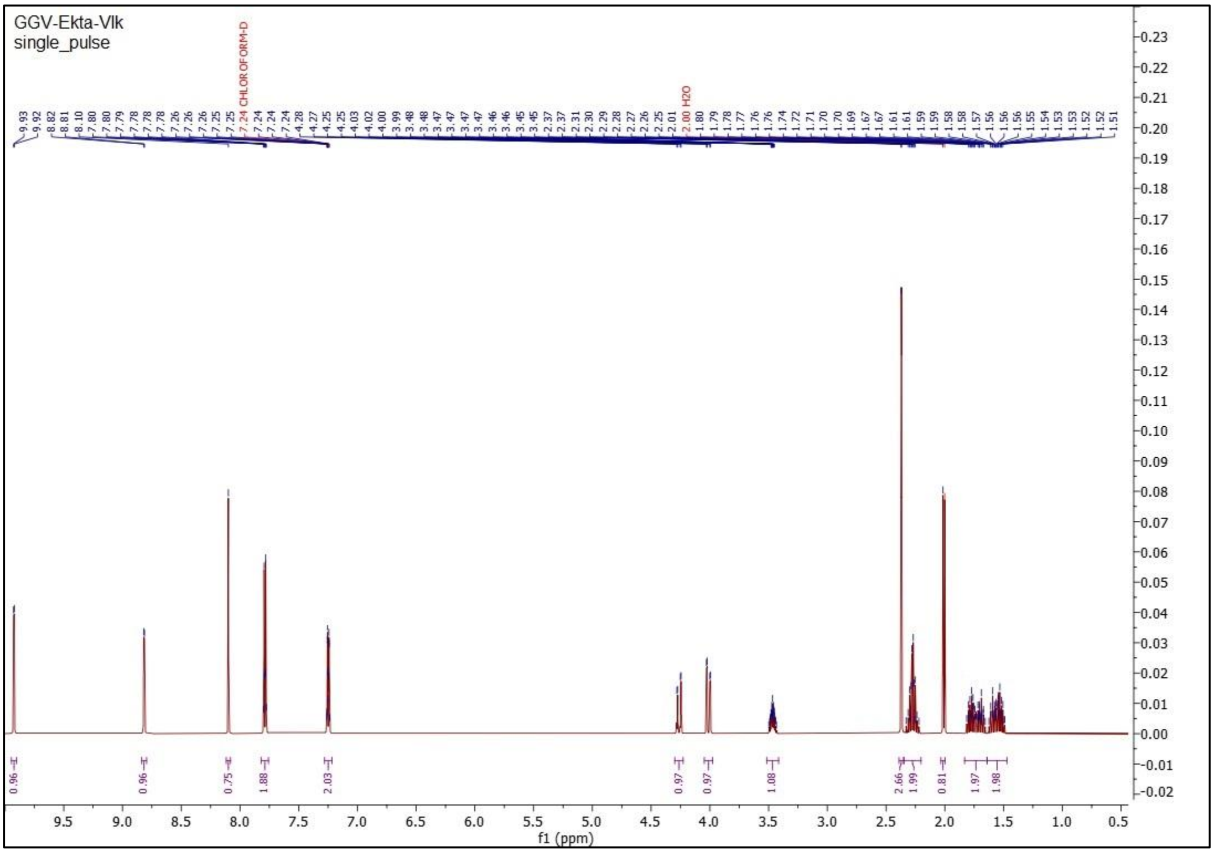

^{13C}NMR-spectra of compound VI(k)

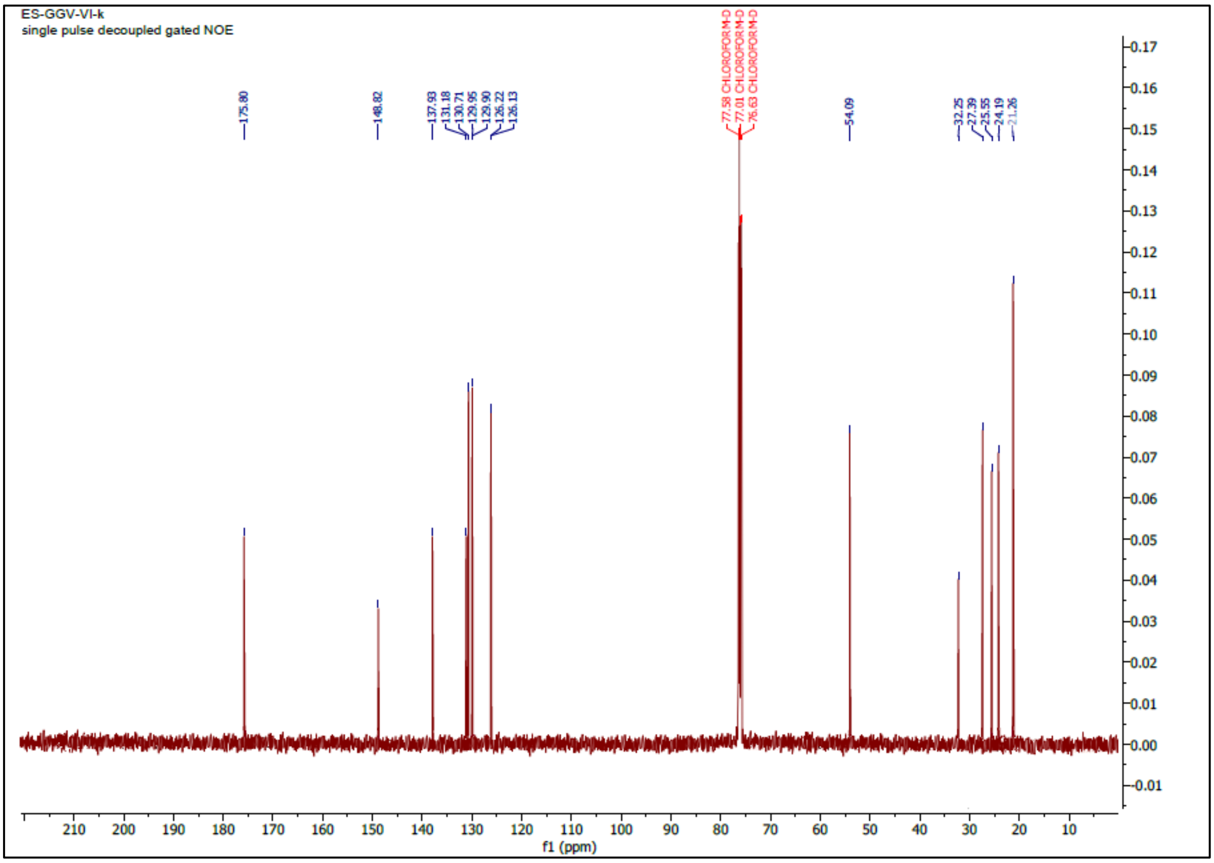

Mass-spectra of compound VI(k)

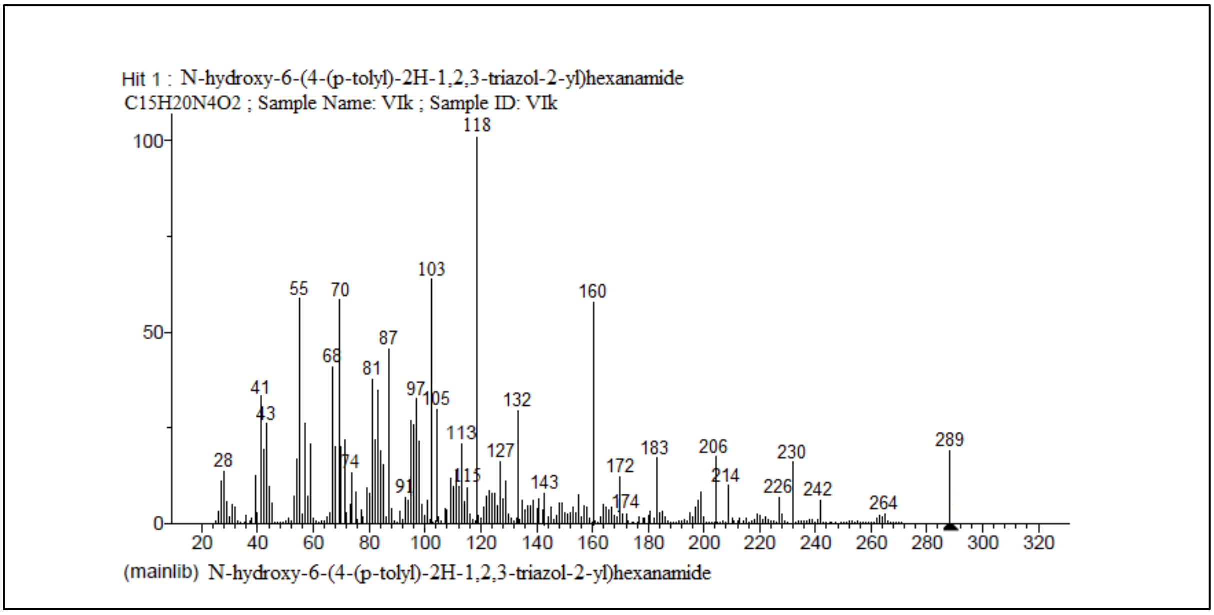

Spectral data of compound VI(l)

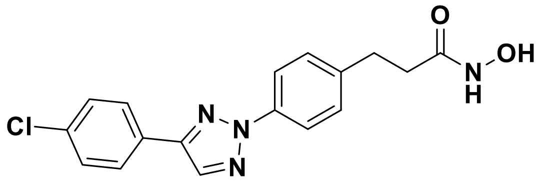

3-(4-(4-(4-chlorophenyl)-2*H*-1,2,3-triazol-2-yl)phenyl)-*N*-hydroxypropanamide
Chemical Formula: $C_{17}H_{15}ClN_4O_2$
Molecular Weight: 342.78

IR-spectra of compound VI(l)

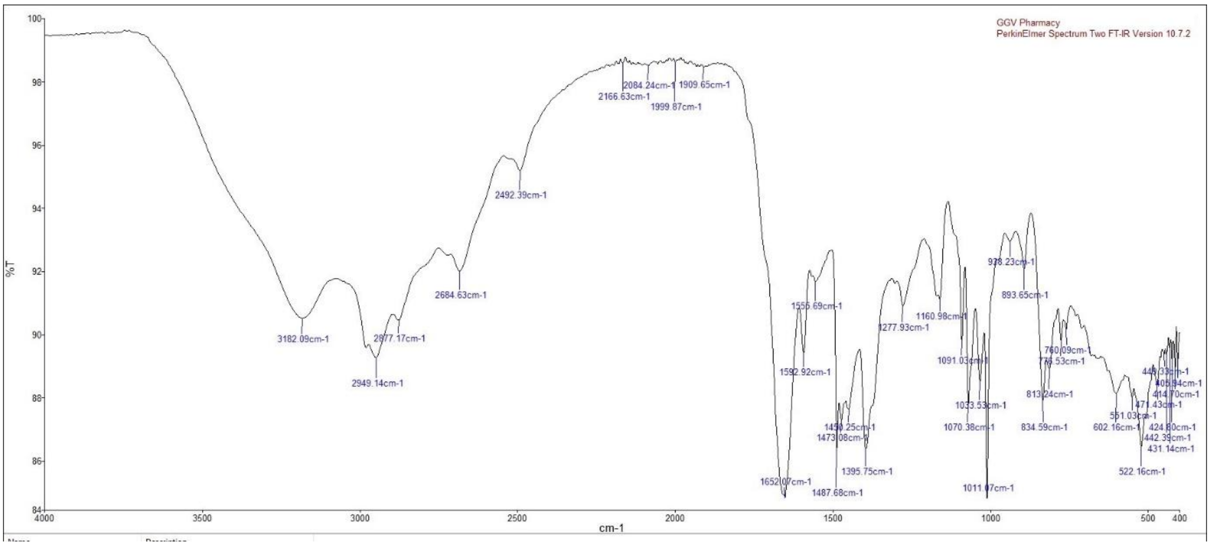

H[1]-NMR-spectra of compound VI(l)

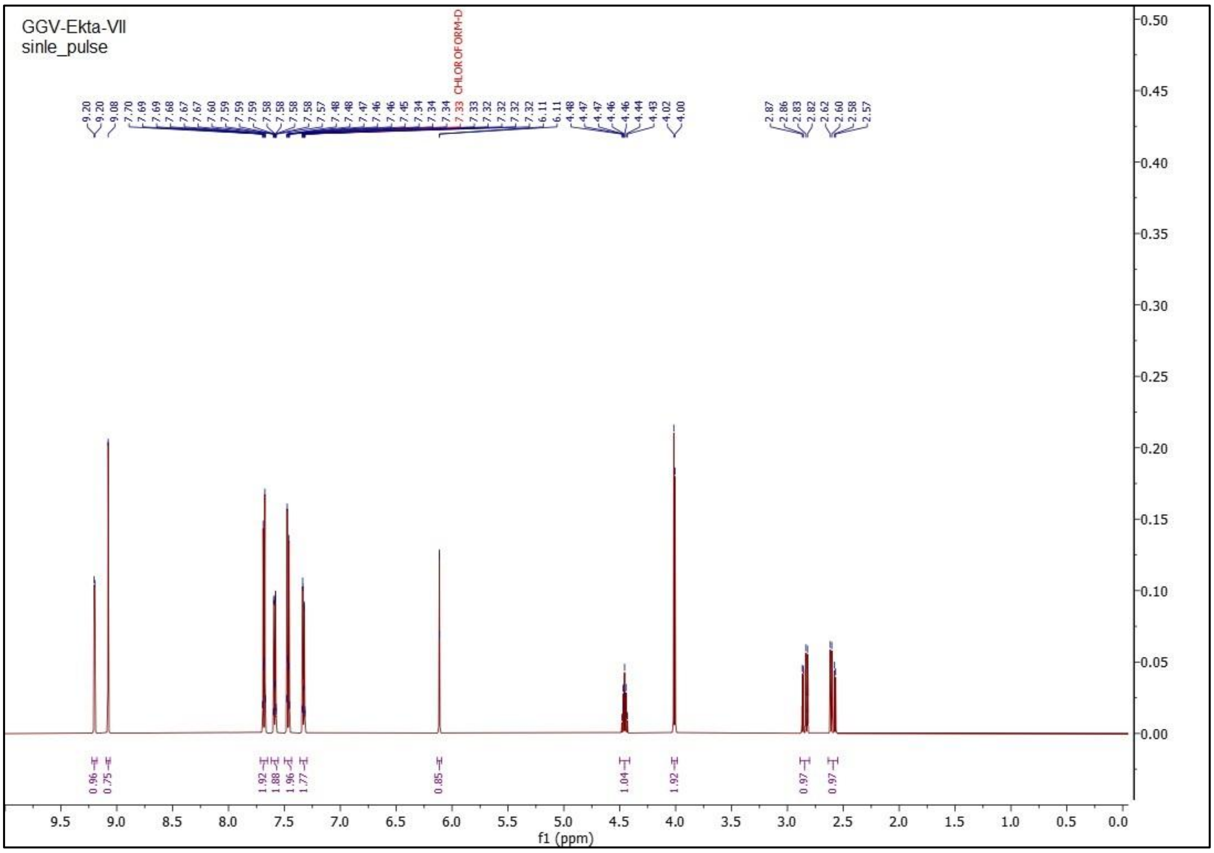

[13C]NMR-spectra of compound VI(l)

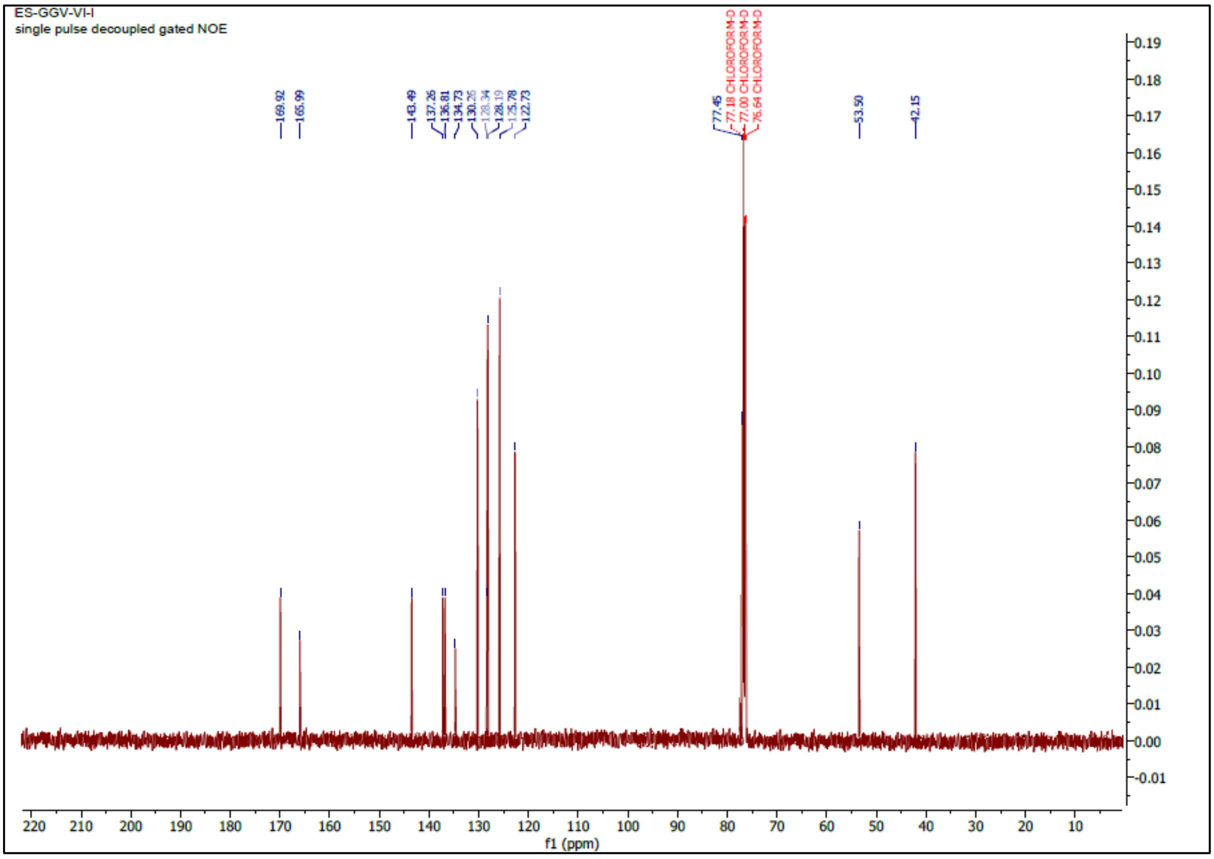

Mass-spectra of compound VI(l)

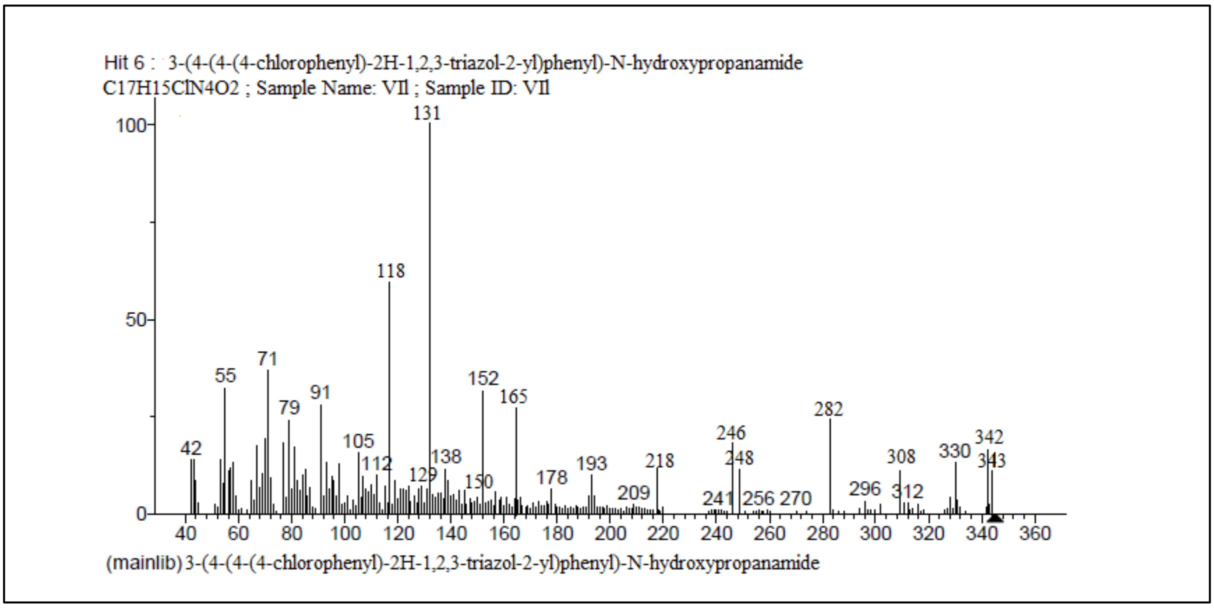

Spectral data of compound VI(m)

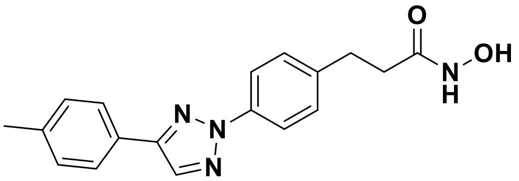

N-hydroxy-3-(4-(4-(*p*-tolyl)-2*H*-1,2,3-triazol-2-yl)phenyl)propanamide
Chemical Formula: $C_{18}H_{18}N_4O_2$
Molecular Weight: 322.37

IR-spectra of compound VI(m)

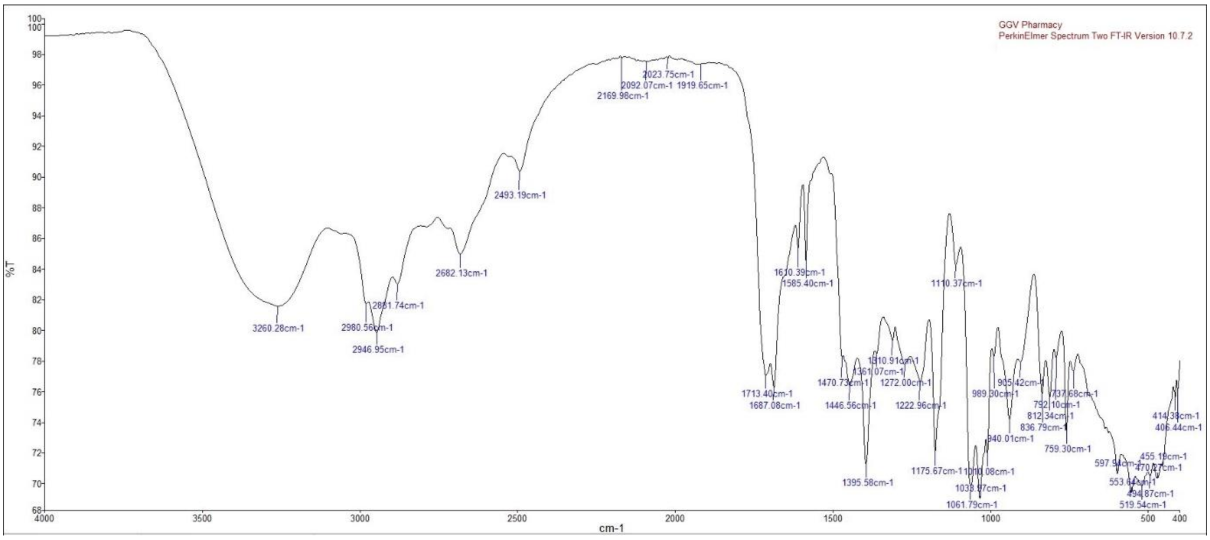

H[1]-NMR-spectra of compound VI(m)

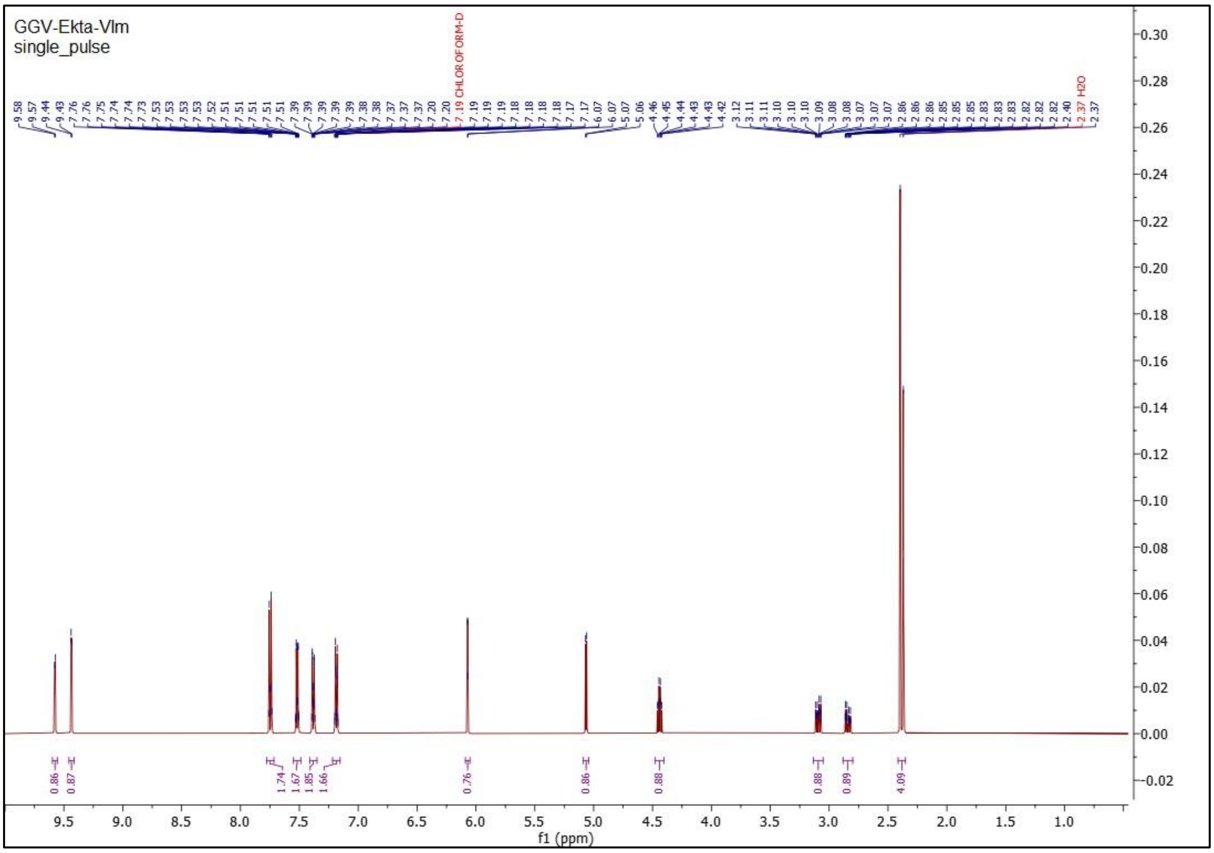

[13C]NMR-spectra of compound VI(m)

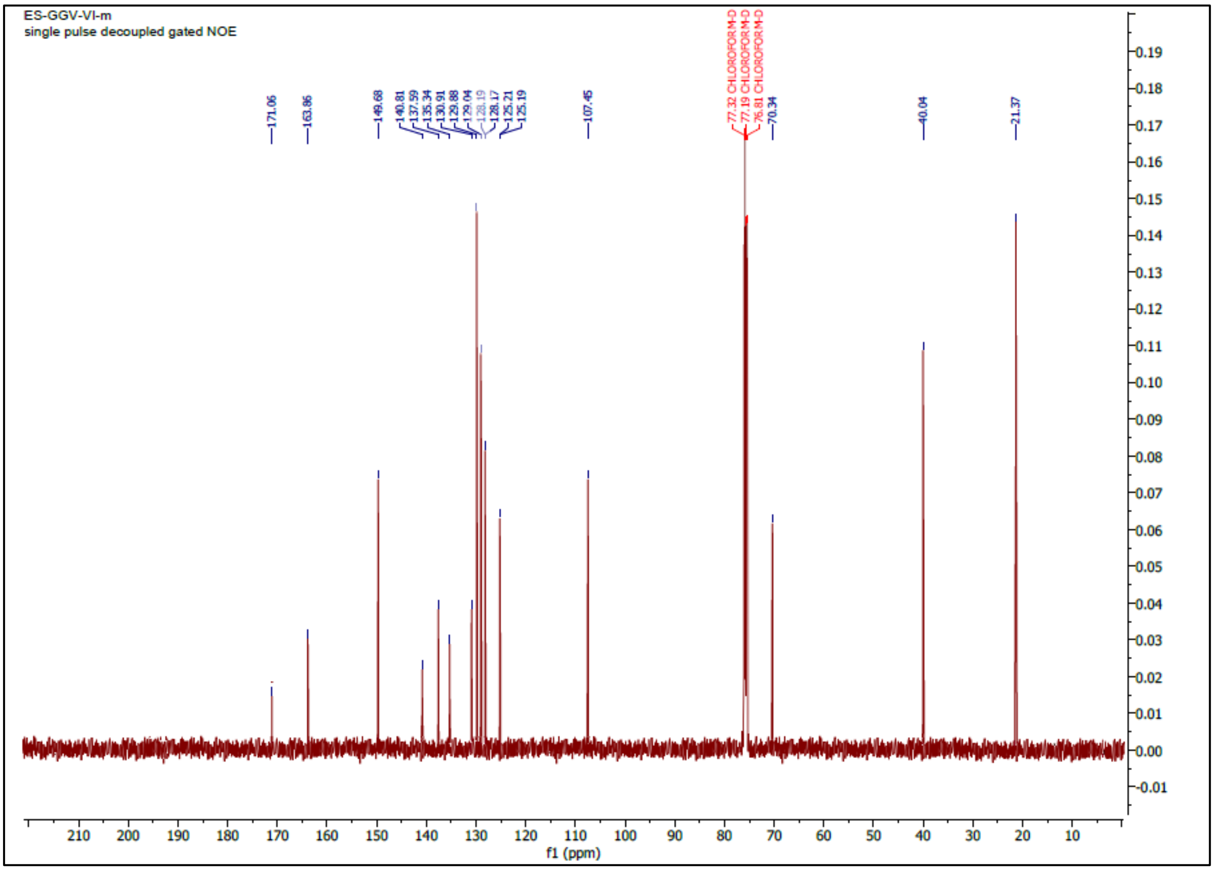

Mass-spectra of compound VI(m)

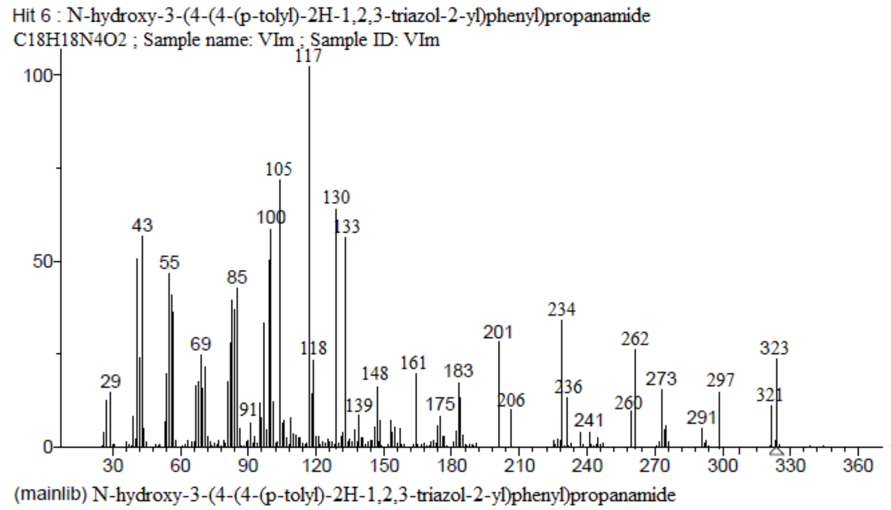

Spectral data of compound VI(n)

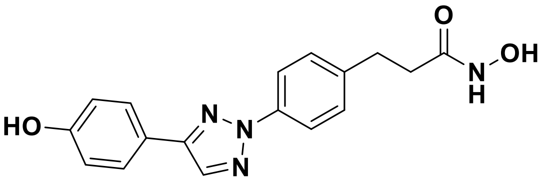

N-hydroxy-3-(4-(4-(4-hydroxyphenyl)-2*H*-1,2,3-triazol-2-yl)phenyl)propanamide
Chemical Formula: $C_{17}H_{16}N_4O_3$
Molecular Weight: 324.34

IR-spectra of compound VI(n)

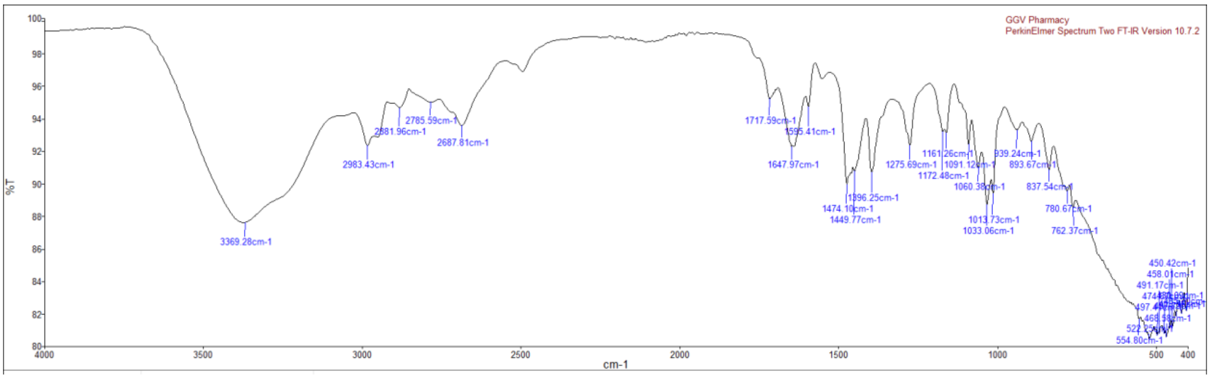

H¹-NMR-spectra of compound VI(n)

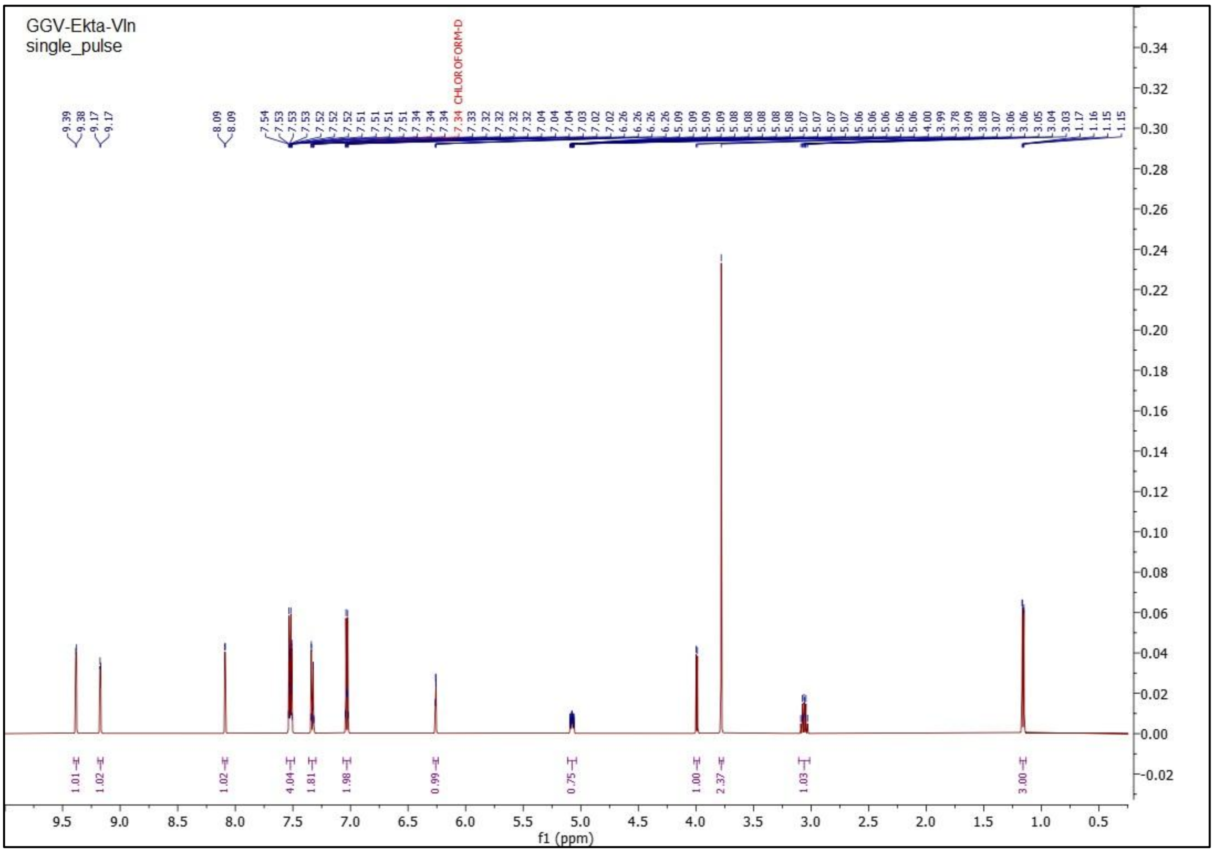

^{13C}NMR-spectra of compound VI(n)

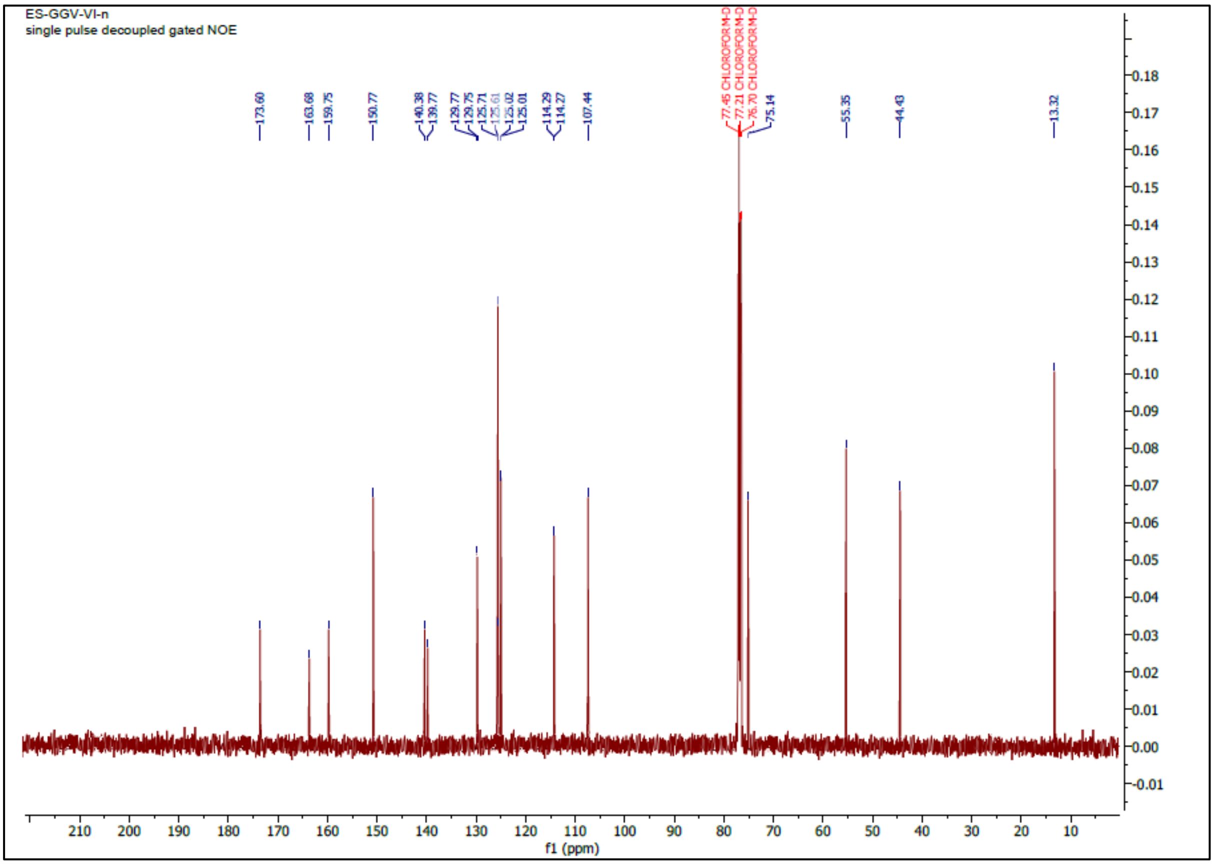

Mass-spectra of compound VI(n)

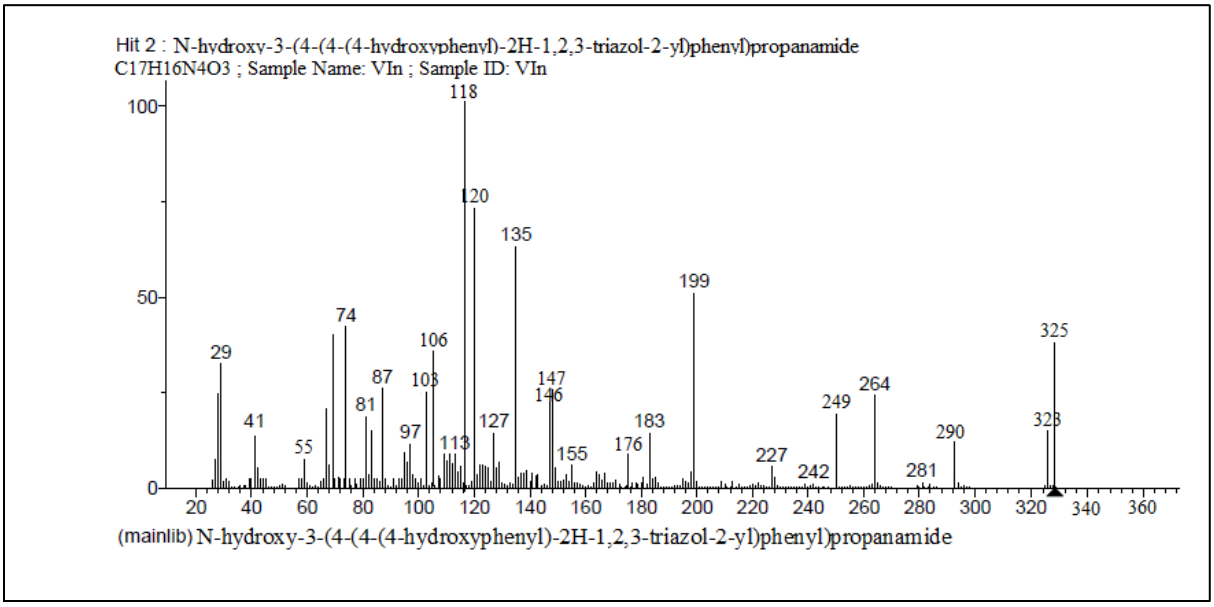

Spectral data of compound VI(o)

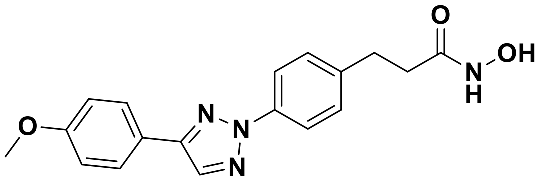

N-hydroxy-3-(4-(4-(4-methoxyphenyl)-2*H*-1,2,3-triazol-2-yl)phenyl)propanamide
Chemical Formula: $C_{18}H_{18}N_4O_3$
Molecular Weight: 338.37

IR-spectra of compound VI(o)

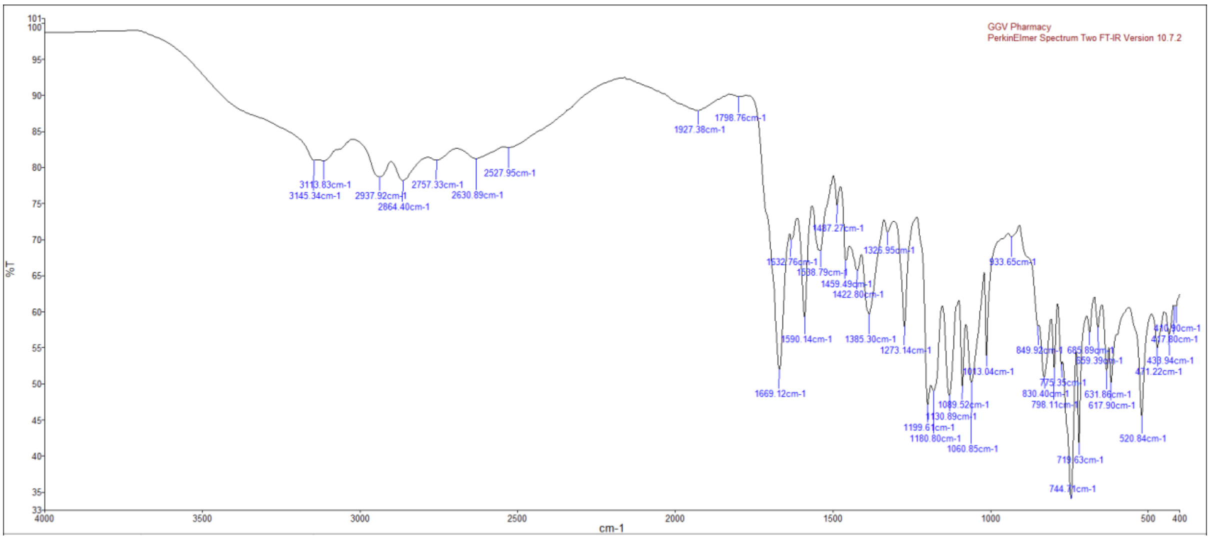

H[1]-NMR-spectra of compound VI(o)

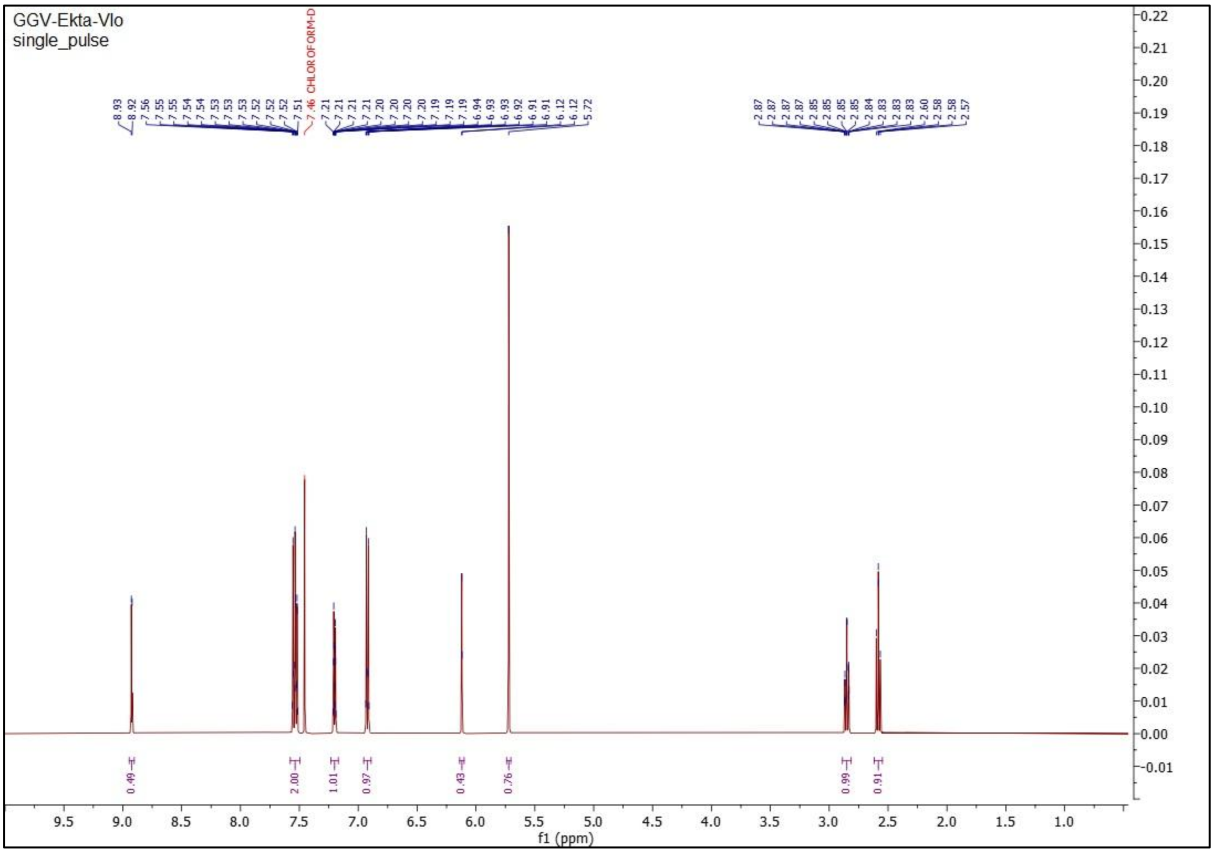

^{13C}NMR-spectra of compound VI(o)

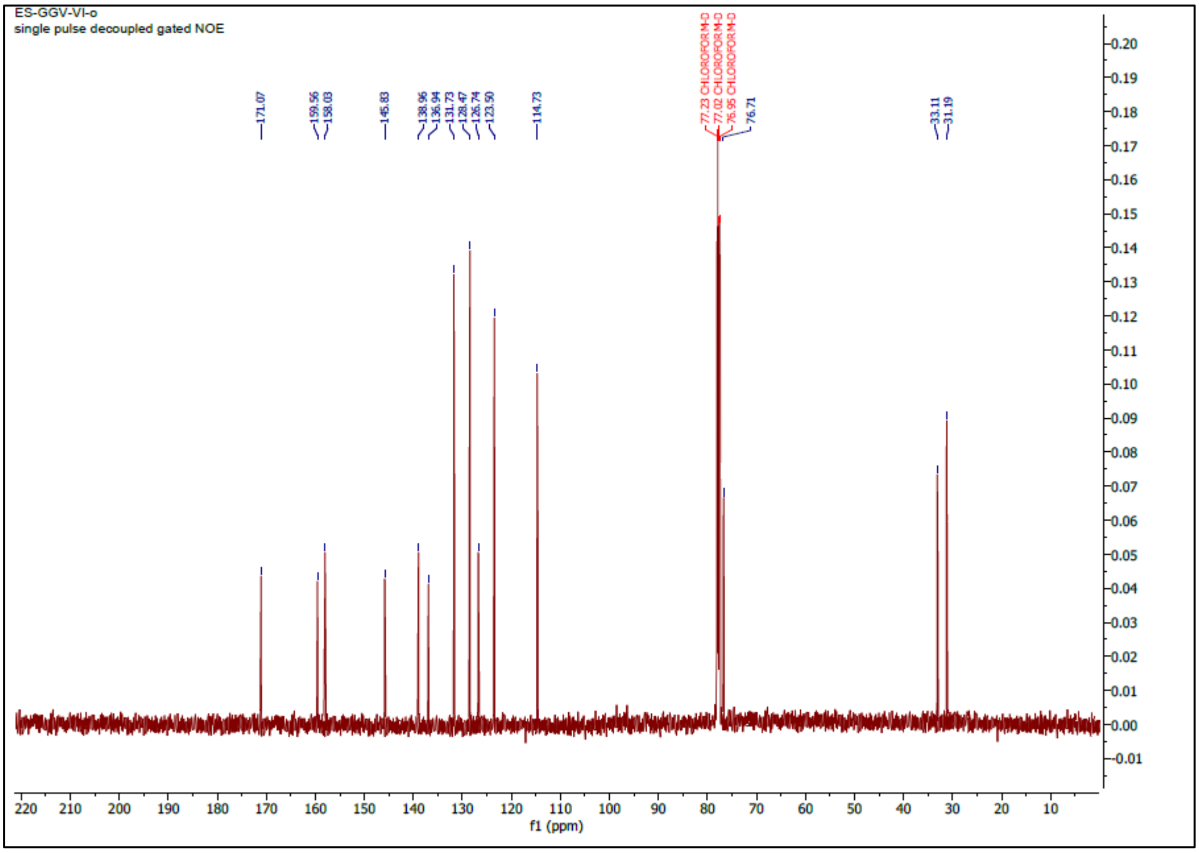

Mass-spectra of compound VI(o)

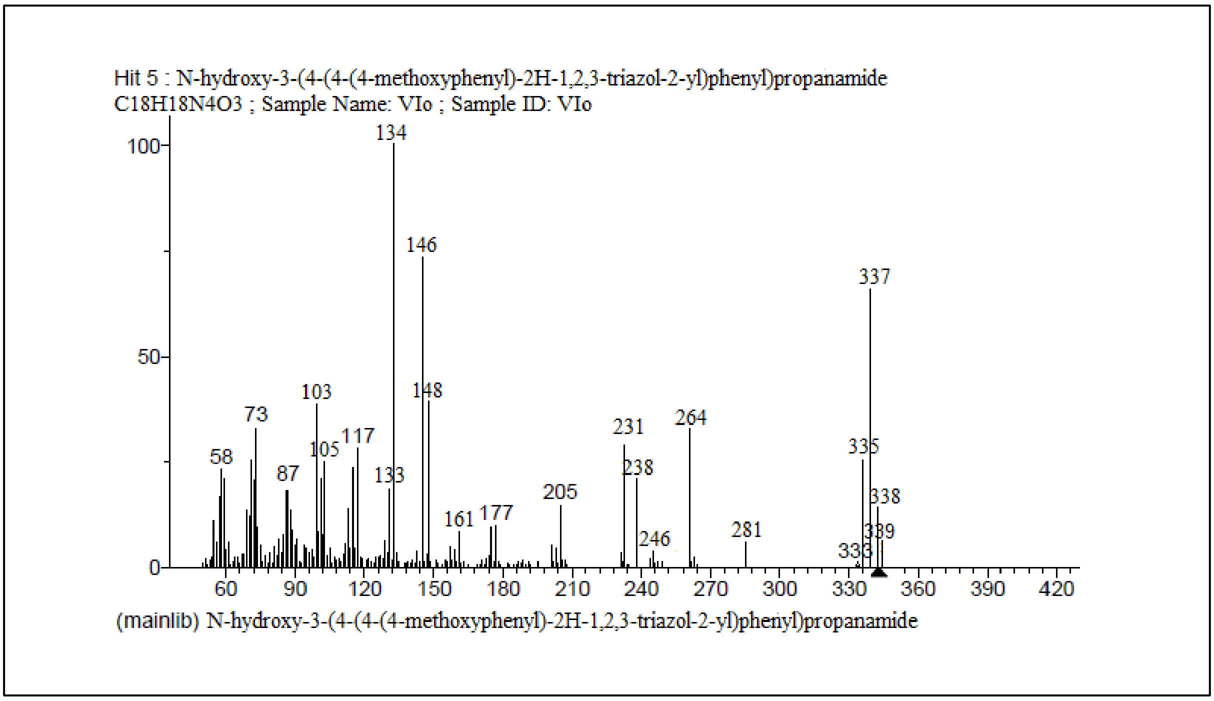

Molecular docking studies

Receptor Preparation

The crystallographic assembly of HDAC1 and HDAC6 protein was accessed form the protein data bank [PDB entry:1C3S and 5EEI respectively]. The selection technique of protein hinge on their pertinence with cancer, the structural similarity of designed compounds with the co-crystallized ligand and most importantly better resolution. The *Protein Preparation Wizard* module in Maestro interface *v* 9.3 was employed to prepare and process the protein crystal before proceeding with docking [49]. The task involves adding hydrogen atoms, nullifying the distantly sited side chain from binding sites and allocating protonation statuses and partial charges with the optimized potentials for liquid simulations 3e (OPLS3e) force field [50].

Ligand Preparation

The structures of designed compounds were drafted by ChemDraw Professional and saved in *.mol format. It was improved by *LigPrep v* 2.5 module by eliminating disordered structures, spawning stereoisomers, creating ionization state of ligands at pH 7.0 ± 2, including hydrogen atoms, confiscating counter ions and pruning the energy with OPLS3e force field [51]. The generated 3D structures of ligands possessed minimal energy though retaining the ingenuity of chirality and ionisation. OPLS3e was the applied force field, producing minimal energy structures with improved chirality [52]. SAHA, a known hydroxamate-based pan HDAC inhibitor, was considered as positive control.

Molecular Docking

Molecular docking reflects an intricate binding interaction between ligands and receptors. Glide provides options for speed as well as precise docking options oscillating from, high-throughput virtual screening (HTVS), which can screen huge libraries, to standard precision (SP), which can screen a number of compounds with good meticulousness, and finally to extra precision (XP), which can remove false positives using very accurate models [52].

Here, the extra precision (XP) mode of *Glide* v 3.4 was employed for docking studies [53]. The Glide "*Receptor Grid Generation*" panel identified the prepared proteins 1C3S and 5EEI as having a functioning catalytic socket. The protein grid was put into use using the standard grid box design of 20 Å × 20 Å × 20 Å dimensions. The Vander Waals radii, charge scaling factor and partial charge cutoff were set to their default values of 1.0, 1.0, and 0.25, respectively. The organized ligands with the lowest energy conformations were prudently chosen and docked in the grid-created protein through XP mode. A unique scoring feature in form of GScore values is provided by the Glide docking approach, which aids in locating the finest potential conformer with the utmost advantageous binding affinity.

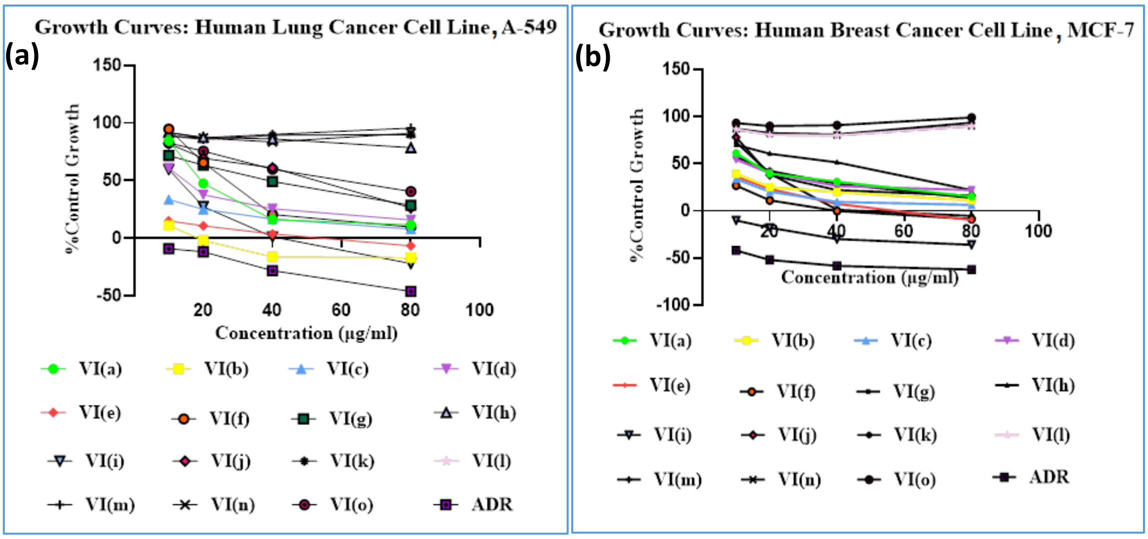

Figure_S1. Growth curve shown by compounds for (a) Human lung (A-549), and (b) Human breast (MCF-7) cancer cell lines, during SRB assay, S.D. < 10%.

Table S1. ADMET analysis of the synthesized compounds using the QikProp and Protox 3.0 tool

Compds	Different drug likeness/predicted ADME descriptors											Organ toxicity		Toxicity end points			Metabolism						Qualitative estimation	Quantitative estimation
	donorHB (0.0 to 6.0)	acceptorHB (2.0 to 20.0)	QPlogP$_{o/w}$ (-2.0 to 6.5)	QPlogBB (-3.0 to 1.2)	QPlogKp (-8.0 to -1.0)	#metab (1 to 8)	QPlogKHsa (-1.5 to 1.5)	Human Oral Absorption (1,2, or	%Human Oral Absorption	Rule of Three (max. 3 violations)	Rule of Five (max. 4 violations)	Hepato-toxicity	Nephro-Toxicity	Carcino-genicity	Muta-genicity	Cyto-toxicity	CYP1A2	CYP2C19	CYP2C9	CYP2D6	CYP3A4	CYP2E1		
**VI(a)**	2	8.95	1.076	-2.028	-3.654	3	-0.53	3	64.823	0	0	Inactive (0.51)	Active (0.56)	Active (0.55)	Active (0.65)	Inactive (0.55)	Inactive (0.84)	Inactive (0.71)	Inactive (0.56)	Inactive (0.69)	Inactive (0.63)	Inactive (0.99)	1000	4

**VI(i)**	VI(h)	VI(g)	VI(f)	VI(e)	VI(d)	VI(c)	VI(b)
2	2	2	2	3	2	2	2
7.45	6.7	9.7	8.2	8.95	8.2	8.2	9.2
1.076	1.471	1.156	1.215	0.293	1.276	1.463	0.286
-1.585	-1.432	-2.141	-1.806	-2.571	-1.963	-1.772	-3.091
-3.125	-3.262	-3.741	-3.672	-4.607	-3.738	-3.707	-5.448
2	1	4	2	3	3	2	3
-0.639	-0.535	-0.537	-0.498	-0.65	-0.396	-0.434	-0.584
3	3	3	3	2	3	3	2
72.143	73.802	65.35	65.727	51.079	66.112	67.181	43.746
0	0	0	0	1	0	0	1
0	0	0	0	0	0	0	0
Active (0.54)	Active (0.53)	Active	Active (0.50)	Inactive (0.50)	Inactiv	Inactive (0.51)	Inactive (0.51)
Active (0.55)	Active (0.63)	Active	Active (0.61)	Active (0.58)	Active	Active (0.61)	Active (0.57)
Active (0.62)	Active (0.61)	Active	Active (0.57)	Active (0.57)	Active	Active (0.55)	Active (0.76)
Active (0.59)	Active (0.62)	Active	Active (0.67)	Active (0.67)	Active	Active (0.66)	Active (0.85)
Inactive (0.62)	Inactive (0.65)	Inactive	Inactive (0.65)	Inactive (0.60)	Inactiv	Inactive (0.66)	Inactive (0.68)
Inactive (0.83)	Inactive (0.85)	Inactive	Inactive (0.80)	Inactive (0.85)	Inactiv	Inactive (0.80)	Inactive (0.83)
Inactive (0.72)	Inactive (0.68)	Inactive	Inactive (0.72)	Inactive (0.77)	Inactiv	Inactive (0.71)	Inactive (0.76)
Inactive (0.61)	Inactive (0.53)	Inactive	Inactive (0.55)	Inactive (0.61)	Inactiv	Inactive (0.52)	Inactive (0.60)
active (0.54)	active (0.56)	Inactive	Inactive (0.63)	Inactive (0.72)	Inactiv	Inactive (0.62)	Inactive (0.71)
Inactive (0.69)	Inactive (0.76)	Inactive	Inactive (0.68)	Inactive (0.70)	Inactiv	Inactive (0.68)	Inactive (0.71)
Inactive (0.99)	Inactive (0.98)	Inactive	Inactive (0.99)	Inactive (0.99)	Inactiv	Inactive (0.99)	Inactive (0.99)
1000	1000	1000	1000	1000	1000	1000	1000
4	4	4	4	4	4	4	4

VI(o)	VI(n)	VI(m)	VI(l)	VI(k)	VI(j)
2	3	2	2	2	3
6.95	6.95	6.2	6.2	6.7	7.45
1.769	0.952	1.967	2.153	1.267	0.275
-1.463	-2.001	-1.407	-1.221	-1.551	-2.132
-2.968	-3.922	-3.059	-3.039	-3.231	-4.092
3	3	3	2	2	2
-0.288	-0.444	-0.157	-0.196	-0.511	-0.774
3	3	3	3	3	2
75.968	61.979	77.123	78.261	73.282	58.158
0	0	0	0	0	0
0	0	0	0	0	0
Inactive (0.50)	Active (0.50)	Inactive (0.51)	Active	Inactive (0.51)	Inactiv
Active (0.55)	Active (0.57)	Active (0.54)	Active	Active (0.59)	Active
Active (0.59)	Active (0.63)	Active (0.73)	Active	Active (0.73)	Active
Active (0.67)	Active (0.68)	Active (0.69)	Active	Active (0.65)	Active
Inactive (0.59)	Inactive (0.65)	Inactive (0.70)	Inactiv	Inactive (0.62)	Inactiv
Inactive (0.76)	Inactive (0.77)	Inactive (0.75)	Inactiv	Inactive (0.85)	Inactiv
Inactive (0.65)	Inactive (0.71)	Inactive (0.72)	Inactiv	Inactive (0.76)	Inactiv
Inactive (0.52)	Inactive (0.57)	Inactive (0.60)	Active	Inactive (0.67)	Inactiv
Inactive (0.62)	Inactive (0.63)	Inactive (0.55)	Inactiv	active (0.51)	active
Inactive (0.60)	Inactive (0.66)	Inactive (0.63)	Inactiv	Inactive (0.76)	Inactiv
Inactive (0.99)	Inactive (0.99)	Inactive (0.99)	Inactiv	Inactive (0.98)	Inactiv
1000	1000	1000	1000	1000	1000
4	4	4	4	4	4

where; donorHB: estimated H-bonds donated by solute to water molecule in an aqueous solution, aceptHB: estimated hydrogen bonds accepted by solute from water molecule in an aqueous solution, $QPlogP_{o/w}$: predicted octanol/water partition coefficient, QPlogBB: predicted brain/blood partition coefficient, QPlogKp: predicted skin permeability, #metab: number of likely metabolic reactions, QPlogKhsa: prediction of binding to human serum albumin.
